# Semi-automated, evidence-based workflow for selection of reference chemicals for the validation of NAMs: a case study with the adipogenesis assay

**DOI:** 10.1016/j.namjnl.2026.100112

**Published:** 2026-07-08

**Authors:** Hana C.M. Farnezi, Sander B.I. Lentz, Juliette Legler, Jorke H. Kamstra

**Affiliations:** Institute for Risk Assessment Sciences, Department of Population Health Sciences, Utrecht University. Yalelaan 104, 3508 TD, Utrecht, the Netherlands

**Keywords:** New approach methodologies (NAMs), Assay validation, Large language models (LLMs), Artificial intelligence, Adipogenesis, Chemical selection, Reference chemicals

## Abstract

The validation of in vitro New Approach Methodologies (NAMs) requires the use of well-characterized reference chemicals to assess assay performance, reproducibility, and relevance. However, selecting such chemicals is often labor-intensive and lacks standardization. We developed a semi-automated, evidence-based workflow that integrates systematic literature review, AI-assisted data extraction, and quantitative evidence scoring to improve the efficiency and transparency of chemical selection. We conducted a structured EMBASE search, followed by AI-assisted abstract screening and data extraction, in a case study to select reference chemicals for the validation of an in vitro adipogenesis assay. Chemicals were evaluated for in vitro, in vivo, and human evidence of adipogenic or obesogenic effects. From 11,648 screened publications, 236 studies met the inclusion criteria, resulting in the identification of 243 candidate reference chemicals, of which 50 were prioritized based on scoring. The final selection encompassed a set of 22 chemicals with different levels of potency and diverse structures. This workflow illustrates how AI-assisted evidence synthesis can accelerate and standardize the selection of reference chemicals while maintaining expert oversight and ensuring regulatory relevance. It provides a reproducible and adaptable framework for future NAMs validation studies across diverse toxicological endpoints.

## Highlights

• AI-assisted chemical selection provides an objective, standardized, and time-efficient chemical selection methodology.
• The approach was tested using adipogenesis (in vitro) as a surrogate measure for adiposity (in vivo) as a case study.
• A scalable framework was developed that is adaptable to other New Approach Methodologies (NAMs).
• Candidate chemicals were identified as suitable for further in vitro validation of adipogenesis.

## Introduction

The effective validation of in vitro New Approach Methodologies (NAMs) relies heavily on selecting appropriate reference chemicals to ensure accurate assessment of the robustness of NAMs in terms of performance and relevance (Hartung, 2024). Assay performance is typically evaluated by sensitivity, specificity, and reproducibility. Sensitivity reflects the ability of the assay to correctly identify chemicals that are truly linked to the biological effect of interest (true positives), whereas specificity reflects its ability to correctly identify chemicals that do not cause effect (true negatives). Reproducibility encompasses intra- and inter-laboratory variability. Relevance refers to the chemical and biological applicability domains of the NAMs, as determined by the chemical diversity represented in the reference set and by concordance with in vivo or human data. Assessment of these criteria during validation calls for the need for careful chemical selection (OECD, 2005).

Assembling appropriate, reliable reference chemicals is notably time-consuming and resource-intensive (ICCVAM, 2024), yet extremely important, as assessment of assay robustness relies on a proper set of validation chemicals. Chemical selection approaches generally combine literature reviews with independent expert judgment. Teams of experts define the context of use, conduct broad multi-database searches, retrieve and assess primary studies, reconcile conflicting findings to determine positive or negative status and potency, and evaluate feasibility factors such as solubility, stability, availability, and regulatory constraints (e.g. Ozcagli et al. (2024) and Kubickova and Jacobs (2023)).

From the limited publications on chemical selection for validation, semi-automated literature-mining workflows have demonstrated potential for efficiently identifying and shortlisting candidate chemicals while maintaining methodological rigor. For instance, Judson et al. (2019) described a semi-automated approach for assembling reference chemical sets by mining large public databases (e.g., ToxCast, PubChem, ChEMBL) for bioactivity evidence, followed by automated annotation of target-mode relationships (agonist vs antagonist) and manual expert curation. This method allowed scalable generation of reference chemicals across many molecular initiating events, although endpoint specificity and direct links for evidence to human disease remained limited (Judson et al., 2019). Such methods, together with the use of large language models (LLMs), can provide a more streamlined approach for reliable chemical selection (Hartung and Kleinstreuer, 2025).

Here, we developed a structured, systematic LLM-assisted workflow to accelerate the selection of chemicals for assay validation, while maintaining a rigorous assessment of chemical properties. Our aim was to design a transparent, reproducible workflow for chemical selection that aligns with regulatory validation principles and is compatible with the integration of AI technologies. To evaluate the effectiveness of our semi-automated workflow, we used an assay which measures adipogenesis as a case study. The need to develop methods for identifying chemicals which play a role in metabolism disorders such as obesity has been recognized (e.g. ECHA, 2025), yet no validated in vitro or in vivo test guidelines for adipogenesis exist (Legler et al., 2020). The measurement of adipogenesis using human mesenchymal stem cells (hMSCs) derived from human donors provides a human-relevant in vitro model for metabolic disruption that may be predictive of increased adiposity and dysfunctional adipose in vivo (Kassotis et al., 2022). The fate of stem cells to differentiate into the lineage of adipocytes, as well as the function of adipose tissue, can be measured with this assay. Both of these processes are known key characteristics of metabolism disrupting chemicals (La Merrill et al., 2024).

**Fig. 1:**
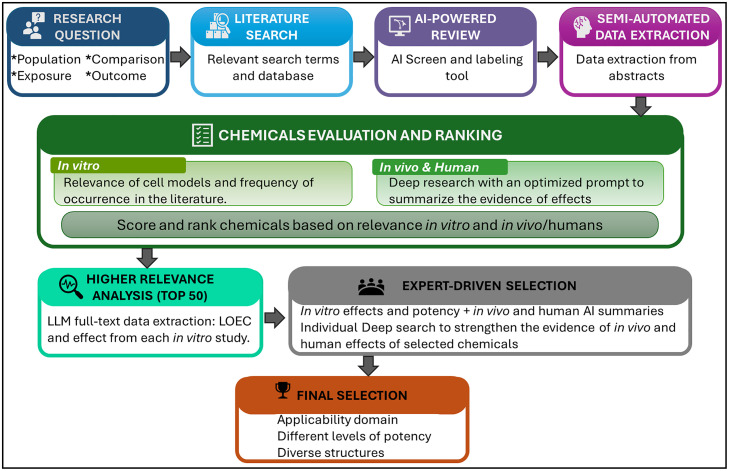
Workflow for the selection and prioritization of reference chemicals for New Approach Methodologies (NAMs). The process starts with formulating the research question using the PECO framework (Population, Exposure, Comparison, Outcome), followed by a comprehensive literature search incorporating relevant terms for each PECO element. Study screening and labeling are facilitated by AI-based tools for systematic reviews. Abstracts from selected studies are analyzed using LLMs to extract information on investigated chemicals and associated cell models. Subsequently, LLM-assisted deep research is applied to identify and synthesize in vivo and human evidence for all identified chemicals. Chemicals are ranked based on the relevance and frequency of in vitro models and the strength of in vivo/human evidence. For the top-ranked chemicals (top 50), full-text articles are further analyzed using LLMs to extract quantitative effect data, including lowest observed effect concentrations (LOECs) and associated biological outcomes. Final chemical selection is guided by expert evaluation to ensure applicability domain, potency, and structural diversity.

To this end, we have developed robust protocols for the hMSC adipogenesis assay (Flores Gomez et al., 2026; Ren et al., 2025), and have carried out pre-validation studies in three naïve labs in the context of the European GOLIATH project (Legler et al., 2020; Hoffman et al., in prep). The adipogenesis assay appears sufficiently robust to proceed to full validation, requiring a full set of reference compounds. For this purpose, we developed a systematic LLM-assisted workflow approach (Fig. 1) and compared it to a previously published expert-curated chemical selection consisting of a limited set of 12 reference compounds used in the GOLIATH pre-validation studies (Ozcagli et al., 2024). This allowed us to directly compare the performance of our workflow against an established expert-driven selection, using the same chemicals as a benchmark.

## Methods

### Search strategy

Our literature review aimed to address the following research questions:

1. Which chemicals are able to induce adipogenesis in vitro?
2. Which of those chemicals can affect adiposity or cause body weight change in vivo?

To address research question 1, a Population, Exposure, Comparison, and Outcome (PECO) framework was established to clearly identify chemicals that affect adipogenesis in animal and human cell models (Table 1). We selected EMBASE as our primary database due to its comprehensive coverage of biomedical literature and structured indexing system (Emtree). This indexing enhances the sensitivity and specificity of searches by effectively capturing relevant studies. EMBASE's commands, such as '/mj' to focus on major topics and '/exp' to include narrower terms, further improve the precision and comprehensiveness of our search.

**Table 1:** PECO statement and search strategy. Search strategy using Emtree terms and filters in Embase. /de = descriptor (indexed term), /mj/exp = major topic with explosion (includes narrower terms), /py = publication year filter, /it = publication type, /lim = language limit.

**PICO element**	**Evidence**	**Search Terms**
**Population** **(P)**	In vitro models for adipogenesis.	('animal cell'/de OR 'animal cell culture'/de OR 'cell culture'/de OR 'human cell'/de OR 'human cell culture'/de OR '*in vitro* study'/de)
**Exposure** **(E)**	Exposure to chemicals, including pharmaceuticals, environmental contaminants, and other compounds. This includes chemicals with known positive, negative, or unknown effects on the outcome.	('agents affecting metabolism'/mj/exp OR 'agents interacting with transmitter, hormone or drug receptors'/mj/exp OR 'environmental, industrial and domestic chemicals'/mj/exp OR 'general and inorganic chemicals'/mj/exp OR 'hormones and agents acting on the endocrine system'/mj/exp OR 'organic compound'/mj/exp)
**Comparison (C)**	No exposure, negative controls.	
**Outcome** **(O)**	Outcomes related to adipogenesis.	('adipo*':ti,ab,kw) AND ('obesity':ti,ab,kw OR 'weight':ti,ab,kw OR 'lipid*':ti,ab,kw OR 'metabolism':ti,ab,kw OR 'expos*':ti,ab,kw OR 'chemical*':ti,ab,kw OR 'pharma*':ti,ab,kw OR 'drug':ti,ab,kw OR 'obesogen*':ti,ab,kw OR 'endocrine disruptor':ti,ab,kw)
**Study type**		('article'/it OR 'article in press'/it OR 'preprint'/it) AND [2000–2025]/py AND [english]/lim

Chemical terms were extracted directly from the structured Emtree to represent our intervention criteria. Using the '/mj/exp' command ensured that our searches covered major terms and all related narrower terms. The inclusion of terms such as 'agents affecting metabolism' captured a wide range of compounds directly related to metabolic disruption. Incorporating 'agents interacting with transmitter, hormone, or drug receptors' specifically addresses key signaling pathways linked to adipogenesis. Additionally, including 'environmental, industrial, and domestic chemicals' expanded the scope to everyday chemical exposures, enhancing practical and regulatory relevance. Terms such as 'general and inorganic chemicals,' 'hormones and agents acting on the endocrine system,' and 'organic compounds' were also incorporated to ensure comprehensive coverage of diverse chemical groups involved in adipogenic processes.

Titles, abstracts, and keywords were used to specifically target outcomes related to in vitro adipogenesis and obesity, ensuring that identified studies were highly relevant to our predefined outcomes. Based on the PECO criteria, our population was specifically restricted to in vitro cell models. The search was limited to English-language articles published from 2000 to 2025 (Table 1). This timeframe was chosen to focus on contemporary research methods, chemical exposures, and relevant regulatory contexts. Importantly, it includes the introduction of the term 'obesogens' in 2006 and captures the subsequent growth in research investigating chemical influences on obesity (Grün and Blumberg, 2006). The EMBASE search was conducted on January 6, 2025.

Although the search was focused on chemicals that induce adipogenesis, it yielded publications that included negatives and anti-adipogenic agents. In subsequent selection procedures, we included the negatives from the publications, but did not focus on anti-adipogenic agents.

### Selection

Title and abstract screening was conducted using PICO Portal (Minion et al., 2021), an AI-enhanced web platform that prioritizes relevant studies based on keyword highlighting, synonym matching, and PECO statement integration. The system iteratively learns from reviewer decisions to display the most pertinent records first and provides screening performance metrics to signal when to conclude abstract screening.

Publications were selected based on our predefined PECO statement (Table 1). Inclusion criteria consisted of studies involving direct chemical exposure, such as pharmaceuticals, environmental contaminants, and other commercially available compounds, with reported adipogenic effects assessed using in vitro models. Exclusion criteria included studies involving mixtures, inhibitors of adipogenesis, dietary supplements, studies that exclusively focus on receptor activation, herbal products, non-English publications, publications lacking full-text accessibility, and those that describe only in vivo or ex vivo models.

### Data extraction

Following the initial screening process, selected publications were downloaded and imported into EndNote. A comprehensive table listing the authors and abstracts of the included studies was generated in EndNote (EndNote, 2013) and subsequently screened using GPT-o4 (OpenAI, 2025) to extract the chemicals and cellular models investigated in each publication. To ensure accuracy and facilitate a systematic evaluation, publications were processed in batches of 50 using a standardized prompt: "This is a table with three columns (number, author, and abstract) and 50 rows. Based on this information, create a table with three columns including reference (author), chemicals investigated, and cell models used for adipogenesis." Following the automated extraction process, the compiled table was manually curated to verify the correct identification of chemicals and cell models, fix any inconsistencies, and ensure the dataset was complete and suitable for analyses (Supplementary material 2.1).

## Selection of candidate chemicals

### In vitro evidence

All publicly available chemical data for compounds identified during abstract screening were retrieved from the EPA’s CompTox Chemicals Dashboard (EPA, 2025). The extracted information included structural identifiers (e.g., CASRN, InChIKey, SMILES), physicochemical properties, in vitro bioactivity profiles, exposure estimates, hazard annotations, and environmental fate data to support downstream analyses.

Next, the chemicals were scored based on the type of cellular model employed and their frequency of occurrence in the reviewed literature. To ensure maximum relevance and reliability, studies utilizing primary human cells received the highest priority, assigned a score of 2.0. Other human-derived cell lines were given a score of 1.5, while animal primary cells and the widely-used 3T3-L1 cell line received a score of 1.0, as being less human-relevant.

### In vivo *and human evidence*

To assess the biological relevance of the identified chemicals, the availability and strength of relevant human and in vivo evidence concerning adipogenic effects were evaluated. Chemical names and identifiers from CompTox were uploaded to Deep Research via GPTo3 (OpenAI, 2025), using a predefined prompt (Supplementary material 1: GPT PROMPT 1). GPTo3 Deep Research autonomously conducts extensive online research by synthesizing and analyzing large volumes of text, images, and PDFs, subsequently generating detailed, citation-supported reports (OpenAI, 2025).

The tool was instructed to summarize available human and animal studies assessing effects related to adiposity or obesogenic endpoints and to classify the overall weight of evidence as strong, limited, no association, negative association, or not studied (N.S.). Classifications were based on concordance across studies: strong evidence indicated a majority of positive findings across multiple studies; limited evidence reflected heterogenous findings; no effect indicated consistent null results; and negative effect indicated consistent inverse associations. Chemicals for which no relevant studies were identified were marked as N.S. Scores were then manually assigned to each classification to enable quantitative comparison. Numerical scores were then manually assigned: strong (majority of data shows a positive relationship with adiposity or weight gain) (2.0), limited (heterogenous results) (1.0), no effect (0), negative effect (−1.0), and N.S. (no score assigned).

Once the separate in vivo and in vitro scores were calculated, both scores were separately ordered from highest to lowest and ranked using the standard competition ranking in MS Excel (=RANK.EQ). Next, the in vivo and in vitro ranks were summed and sorted from lowest to highest, i.e. the lowest cumulative rank was the chemical with the most evidence in all data. From this list, the top 50 chemicals were identified and from these chemicals, the full texts of the initial EMBASE search, were uploaded to GPTo4, to extract the Lowest Observed Effect Concentration (LOEC) and related effects from each study (Supplementary material 1: GPT PROMPT 2) to determine their in vitro potency. The final selection of chemicals from the top 50 was guided by expert evaluation, based on in vitro potency, efficacy and chemical diversity (structure and use).

### *Final selection*

The final list of chemicals was defined based on expert opinion and was selected to represent diverse chemical structures, classes, and different levels of adipogenic potency and effects. They were classified based on their potency in the in vitro models extracted from included studies (Supplementary material 2.2): chemicals with an LOEC below 0.1 µM were considered strong, ranging from 0.1 to 1 µM were considered moderate to strong, 1 to 10 µM were considered weak to moderate, and concentrations higher than 10 µM were considered weak. We also identified potential negatives (Table 2).

**Table 2:** Summary of the selected chemicals based on in vitro, human and in vivo animal evidence. Chemicals were considered strong based on adipogenesis in vitro (LOEC < 0.1 µM, n = 4), moderate to strong (LOEC 0.1 - 1 µM, n = 2), weak to moderate (LOEC 1 - 10 µM, n = 7), weak (LOEC >10 µM, n = 6) or negative (no LOEC, n = 3).

Chemical	Cas No Mol. weight Log P	Structure	*in vitro*	Human	Animal
**Strong based on adipogenesis in vitro**					
Rosiglitazone (often used as positive control)	122,320-73–4 357.4 g/mol 3.1		Rosiglitazone consistently increases adipogenesis in different cell models (Beck et al., 2013; Benvenuti et al., 2007; Patel et al., 2014; van de Vyver et al., 2014)	Multiple clinical trials suggest that rosiglitazone increases body weight and/or changes in fat mass distribution in treated individuals. (Kahn et al., 2010; Punthakee et al., 2014; Ratziu et al., 2008).	Studies in animals have consistently confirmed that rosiglitazone promotes weight gain and adipose tissue accumulation (Pickavance et al., 1999; Pini et al., 2012).
Tributyltin (TBT) (Often used as positive control)	1461–22–9 325.50 g/mol 4.76		TBT increases adipogenesis at low doses in both human and animal cell models (Bastos Sales et al., 2013; Biemann et al., 2014, 2012; Carfi et al., 2008; Grün and Blumberg, 2006; Inadera and Shimomura, 2005; Li et al., 2011; Llobet et al., 2015; Lutfi et al., 2017; Ticiani et al., 2023; Watt and Schlezinger, 2015; Yanik et al., 2011)	TBT detection in the placenta is linked to infant weight gain in the first 3 months (Rantakokko et al., 2014).	Multiple rodent studies shows that TBT exposure consistently increases fat mass and causes metabolic disturbances (Grün and Blumberg, 2006; Penza et al., 2011; Zuo et al., 2011).
Triphenyltin (TPhT)	639–58–7 385.5 g/mol 4.19		TPhP increases adipogenesis at low doses in both human and animal cell models (Lutfi et al., 2017; Ticiani et al., 2023; Watt and Schlezinger, 2015; Yanik et al., 2011).	The only available evidence did not report an association for TPhT (Rantakokko et al., 2014).	No obesogenic effect of TPhT in animals (Grote et al., 2009; Ohhira and Matsui, 1996; Sarpa et al., 2010).
Dibutyltin (DBT)	1002–53–5 234.95 g/mol 3.12		DBT increases adipogenesis at low doses in both human and animal cell models (Chamorro-García et al., 2018; Milton et al., 2017; Ticiani et al., 2023; Yanik et al., 2011)	DBT was detected in placenta, but was not associated with early postnatal weight gain (Rantakokko et al., 2014)	One mouse study showed that DBT exposure can increase adiposity and metabolic disorder markers in male offspring (Chamorro-García et al., 2018)
**Moderate-to-strong based on adipogenesis in vitro**					
Butylparaben (BPB)	94–26–8 194.23 g/mol 3.6		BPB promotes adipogenesis in vitro across different cell models (Hu et al., 2017, 2013).	Evidence linking butylparaben to obesity in humans is mixed, with some prenatal studies suggesting an increase in childhood (Højsager et al., 2021) while adult data show null (Leppert et al., 2020) or inverse associations (Quirós-Alcalá et al., 2018).	Butylparaben promotes adiposity and metabolic disturbances in animals in maternal or adult exposure (Du et al., 2024; Leppert et al., 2020; Maske et al., 2020).
Glibenclamide(GLI)	10,238-21–8 494.0 g/mol 4.8		Glibenclamide induces adipogenesis in primary human cell (Mayer et al., 2011)	Glibenclamide use leads to increased weight gain (Kahn et al., 2006; UKPDS Group, 1998) or reduced weight loss in diabetic patients (Martin et al., 2003).	Glibenclamide can increase fat mass or adipocyte size (Masky et al., 2024; Mori et al., 2004).
**Weak-to-moderate based on adipogenesis in vitro**					
Triphenyl phosphate (TPP)	115–86–6 326.3 g/mol 4.6		TPP increases adipogenesis in both human and animal cell models (Cano-Sancho et al., 2017; Q. Liu et al., 2024; Sun et al., 2024; Tachachartvanich et al., 2024; Tung et al., 2017a, 2017b)	TPP's relationship to adiposity in humans is heterogeneous, with mostly weak or null associations. (Boyle et al., 2019; Chen et al., 2023; Li et al., 2024).	Multiple rodent studies show that TPP exposure increases fat mass and weight (Green et al., 2017; Tachachartvanich et al., 2024; Wang et al., 2019).
Mono-(2-ethylhexyl) phthalate (MEHP)	4376–20–9 278.34 g/mol 4		MEHP increases adipogenesis in both human and animal cell models (Chiu et al., 2018; Ellero-Simatos et al., 2011; C. J. Hao et al., 2012; Watt and Schlezinger, 2015)	Exposure to MEHP and increased adiposity in humans is mixed, with some studies showing modest associations (Buser et al., 2014; Harley et al., 2017; Peng et al., 2022).	One in vivo study showed increased adiposity after perinatal MEHP exposure (C. Hao et al., 2012).
Tetrabromobisphenol A (TBBPA)	79–94–7 543.9 g/mol 6.8		TBBPA increases adipogenesis in both human and animal cell models (Andrews et al., 2020; Chappell et al., 2018; Liu et al., 2020; Watt and Schlezinger, 2015; Woeller et al., 2017; Yu et al., 2024)	TBBPA is linked to low birth weight in males (Liang et al., 2020).	TBBPA increases adiposity in high-fat-fed mice (Ding et al., 2025)but shows no effect in standard rodent studies (Cope et al., 2015).
Pioglitazone (PIO)	111,025-46–8 356.4 g/mol 3.8		Pioglitazone increases adipogenesis in both human and animal cell models (Andrews et al., 2020; Beck et al., 2013; van de Vyver et al., 2014)	Pioglitazone consistently increases adiposity in humans (Dormandy et al., 2009; Kernan et al., 2016; Smith et al., 2005).	Pioglitazone induces dose-dependent increases in body weight and fat accumulation (De Souza et al., 2001; Kusunoki et al., 2011; Matsuura et al., 2015)
2,2,4,4-Tetrabromodiphenyl ether (BDE-47)	5436–43–1 485.79 g/mol 6.2		BDE-47 consistently increased adipogenesis in 3T3-L1 cells (Bastos Sales et al., 2013; Kamstra et al., 2014; Liu et al., 2022; Z. L. Liu et al., 2024; Tung et al., 2014; Yang et al., 2018)	Most epidemiological studies report no association (Erkin-Cakmak et al., 2015; Kupsco et al., 2022; Vuong et al., 2016), but one recent cohort study found increased gestational weight gain linked to BDE-47 (Wang et al., 2024).	Multiple rodent experiments demonstrate that perinatal exposure to low doses of BDE-47 leads to increased offspring body weight (Strunz et al., 2024; Suvorov et al., 2009; Wang et al., 2024).
Sertraline (SRT)	79,617-96–2 306.2 g/mol 4.8		Sertraline increases lipid accumulation during differentiation (Bozdag et al., 2024)	Sertraline is associated with weight gain and higher BMI during long-term treatment (Mwinyi et al., 2024; Petimar et al., 2024)	In animals, available studies show decreased weight/fat gain with sertraline treatment (Nielsen et al., 1992; Silverstein-Metzler et al., 2016)
Polychlorinated biphenyl 180 (PCB180)	35,065-29–3 395.3 g/mol 7.9		PCB180 increases adipogenesis in different cell models (Yu et al., 2021)	Longitudinal studies suggest that PCB180 exposure is associated with higher body mass index (Agay-Shay et al., 2015; Lee et al., 2011). However, cross-sectional data have shown inverse relationships (Dirinck et al., 2011).	No evidence of PCB180-induced obesity in animals (Loiola et al., 2016; Viluksela et al., 2014).
**Weak based on adipogenesis in vitro**					
Bisphenol S (BPS)	80–09–1 250.27 g/mol 1.9		BPS consistently increases adipogenesis in different cell models (Ahmed and Atlas, 2016; Boucher et al., 2016; Choi et al., 2021; Martínez et al., 2020; Ramskov Tetzlaff et al., 2020; Reina-Pérez et al., 2021).	Evidence linking BPS to increased adiposity in humans is heterogeneous, with some studies showing positive associations (Jacobson et al., 2019; Zhang et al., 2019), but others reporting no significant effects (Liu et al., 2019, 2017; Philips et al., 2018)	BPS promotes adiposity in animals, particularly with developmental exposure and high-fat diets (Ahn et al., 2020; Ivry Del Moral et al., 2016; Meng et al., 2019)
Olanzapine (OLA)	132,539-06–1 312.4 g/mol 2.9		OLA stimulates adipogenesis in both rodent and human in vitro models (Matsuo et al., 2022; Nimura et al., 2015; Yang et al., 2007).	OLA consistently increases weight gain in humans (Jain et al., 2006; Lieberman et al., 2005; Perez-Iglesias et al., 2008).	OLA induces adiposity in animals, with findings of increased fat mass and adipocyte size in rodents, independent of weight gain (Albaugh et al., 2010; Hou et al., 2018; Yang et al., 2019)
Dolutegravir (DTG)	1051,375-16–6 419.4 g/mol 2.4		In vitro evidence indicates that DTG can alter increase adipogenesis in both rodent and human adipocyte cells (Gorwood et al., 2020; Perna et al., 2023)	Clinical evidence links dolutegravir, especially with tenofovir alafenamide, to significant weight and fat gain (Bansi-Matharu et al., 2021; Sax et al., 2019; Sokhela et al., 2024).	Dolutegravir alone does not increase fat mass in rodents (Dontsova et al., 2025; Kress et al., 2024), but it does alter adipose tissue remodeling in macaques (Ayissi et al., 2022).
4-Nonylphenol (4-NP)	104–40–5 220.35 g/mol 5.9		4‐NP has shown contradictory effects on adipogenesis: in 3T3‐L1 cells, it has been reported to promote (C. J. Hao et al., 2012) and inhibit adipogenesis depending on the dose (Zhang et al., 2022). In C3H/10T1/2 MSCs, it increased adipogenic differentiation (Zhang et al., 2022).	Evidence linking 4-NP to adiposity in humans is heterogeneous, with some studies showing positive associations (Lopez-Espinosa et al., 2009; Seo et al., 2019; Sung et al., 2006) but a large adult cohort found no significant effect (Park and Kim, 2017)	Animal studies provide consistent evidence that 4-NP increases fat accumulation and body weight (C. J. Hao et al., 2012; Yu et al., 2020, 2017)
Megestrol acetate (MGA)	595–33–5 384.5 g/mol 3.1		Megestrol ng increases adipogenesis in primary human cells (Sung et al., 2015)	Megestrol acetate consistently increases weight gain in humans, with the majority of the gain as fat mass rather than lean (Eubanks et al., 2002; Loprinzi et al., 1990; Von Roenn et al., 1994).	Animal studies demonstrate consistent weight and fat gain in rodents, including the prevention of fat loss in cachectic models (Southam, 1968; Zhong et al., 2023)
Fenthion	55–38–9 278.3 g/mol 4.1		Fenthion consistently increases adipogenesis in different cell models (Andrews et al., 2020).	No direct studies on fenthion and obesity were found.	Repeated low-dose fenthion exposure in rodents suggests potential weight gain (APVMA, 2005)
**Negative based in adipogenesis in vitro**					
Dichlorodiphenyldichloroethylene (ppDDE)	72–55–9 318.0 g/mol 7		ppDDE has little to no effect in adipogenesis (Howell and Mangum, 2011; Mangum et al., 2015; Rubbo et al., 2024)	Developmental exposure to p,p′-DDE has yielded mixed results, with some studies associating it with increased childhood adiposity and (Agay-Shay et al., 2015; Delvaux et al., 2014), while others have found no significant association (Cupul-Uicab et al., 2010; Høyer et al., 2014).	p,p′-DDE moderately promotes fat accumulation and metabolic disruption, but effects vary and are most evident under obesogenic diets (Howell et al., 2015, 2014; Migliaccio et al., 2024).
Bis(2-ethylhexyl) phthalate (DEHP)	117–81–7 390.6 g/mol 7.4		DEHP has little to no effect in adipogenesis (Chiu et al., 2018; Y. Zhang et al., 2024)	Evidence linking DEHP exposure to increased adiposity in humans is mixed, some studies report positive associations (Wen et al., 2024), others find null or sex- and age-specific effects (Deodati et al., 2024), while some even suggest an inverse relationship with BMI (Desalegn et al., 2024; Zhang et al., 2014)	DEHP increases fat mass in rodents, particularly with early-life or low-dose exposure, but inconsistencies arise at very high doses (Gu et al., 2016; Hao et al., 2013; Klöting et al., 2015)
Thiacloprid	111,988-49–9 252.72 g/mol 2.2		Thiacloprid has no effect on 3T3‐L1 adipogenesis (Mesnage et al., 2018)	Evidence for thiacloprid as an obesogen in humans is mixed, with one study linking it to higher adiposity (Lu et al., 2023) but another showing a null association (Wu et al., 2024).	Thiacloprid caused a decrease in body weight in animals (Mahmoud et al., 2024)

## Deep search *in* vivo and human evidence

To strengthen the assessment of in vivo and human evidence, a separate Deep Research analysis was performed for each of the selected chemicals using a structured prompt (Supplementary material 1: GPT PROMPT 3). To evaluate the accuracy of the AI-retrieved references, we conducted a random sampling check rather than verifying all references. A minimum of two references for human studies and two for animal studies were evaluated. If the first two references yielded concordant outcomes, no further verification was performed. In cases of discordant outcomes, an additional reference was assessed. For each sampled reference, we confirmed that (i) the citation existed, (ii) it referred to the correct chemical, and (iii) the reported outcome was accurately represented in the AI-generated summary. This verification step provides an empirical estimate of the reliability of the AI-generated evidence synthesis, informing the level of confidence that can be placed in the results.

## Chemical space representation

To visualize the structural diversity of the selected compounds in relation to a broader chemical context, a principal component analysis (PCA) was performed. Canonical SMILES for 11,849 REACH-registered chemicals were retrieved, duplicates were removed, and a random subset of 3500 unique chemical structures was selected to serve as the background chemical space. The selected chemicals and reference compounds were encoded using Morgan circular fingerprints (ECFP4; radius = 2, 1024 bits) (Rogers and Hahn, 2010) computed via the RDKit cheminformatics toolkit (Landrum et al., 2025). The high-dimensional binary fingerprint matrix was then reduced to two principal components using PCA, as implemented in scikit-learn, which captures the major axes of structural variation (Pedregosa, 2011). Compounds were visualized in a 2D scatter plot.

## Results

**Fig. 2:**
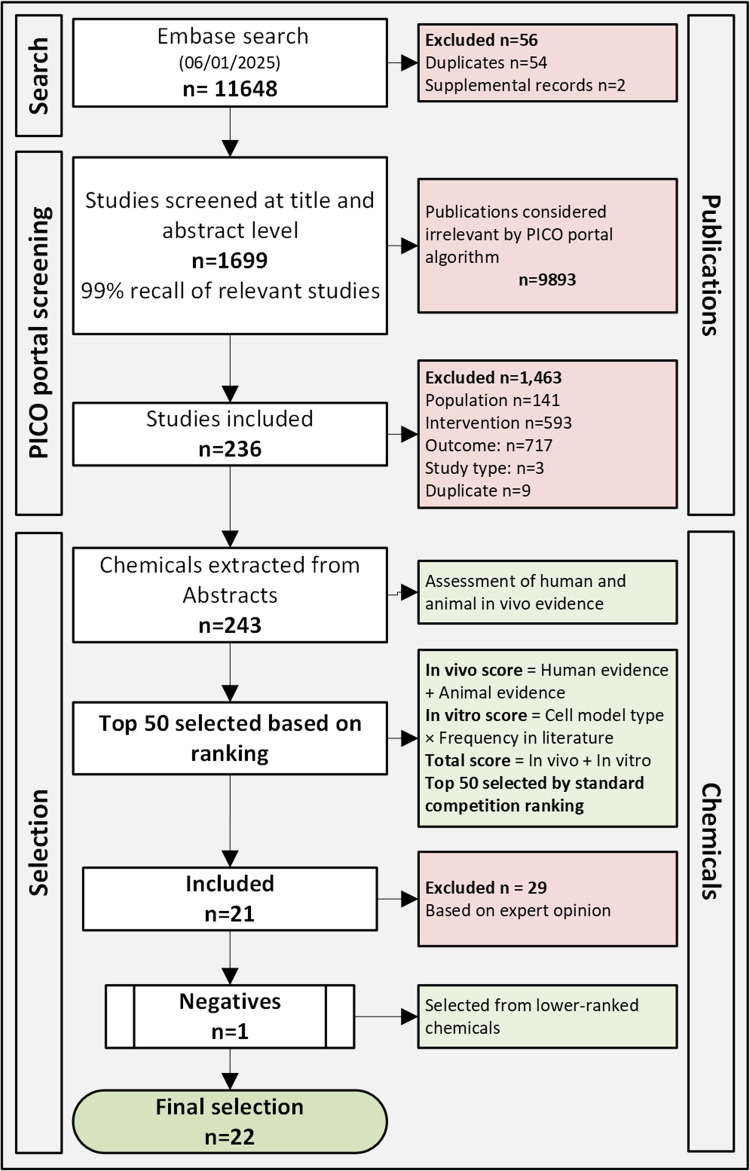
Flow diagram of the semi-automated workflow used to select chemicals for validation of adipogenesis NAMs. A literature search was conducted in Embase in January 2025. Titles and abstracts were screened using the PICO Portal systematic review tool, resulting in the inclusion of 236 studies. From these, 243 unique chemicals were identified. AI-assisted methods were applied to compile evidence on the in vivo and human effects of the identified chemicals. Chemicals were then scored based on the weight of in vitro and in vivo/human evidence, and the top 50 candidates were shortlisted. Finally, expert evaluation refined this list to a final selection of 22 chemicals. Red boxes indicate exclusion and green boxes inclusion steps.

Fig. 2 illustrates the outcome of the workflow for systematically selecting candidate chemicals associated with adipogenesis. The initial search in EMBASE yielded 11,648 publications, which were subsequently uploaded to the PICO Portal for systematic screening. Following the removal of 54 duplicate entries and two supplemental records, we screened titles and abstracts on PICO Portal and stopped screening once the machine learning algorithm reached 99% recall rate of relevant literature, i.e., 1% was predicted to still contain relevant chemicals. Ultimately, the PICO Portal algorithm deemed 9893 publications irrelevant, while 1699 were screened based on their titles and abstracts. Following this screening process, 236 studies were included based on the predefined PICO criteria, with 1463 studies being excluded based on specific criteria: population (n = 141), intervention (n = 593), outcomes (n = 717), study type (n = 3), and additional duplicates (n = 9).

From the 236 included studies, GPT identified 271 compounds from title and abstracts. After fixing duplicates (i.e., identical compounds referred to under different names) and excluding non-eligible entries such as complex or undefined mixtures, high-molecular polymers, ambiguous counter-ions, endogenous compounds, and culture-medium components, a total of 243 candidate chemicals were identified, as well as the cell model they were studied in (Supplementary material 2.1 - Literature search). These chemicals were then evaluated based on in vivo and in vitro evidence, via an integrated scoring of human and animal studies (the ‘in vivo’ score) using a unsupervised prescreen via GPT’s Deep Research, while the in vitro score considered both the type of cell model (primary human cells, human-derived lines, animal cells, or 3T3-L1) and the frequency with which the chemical appeared in the reviewed abstracts. Chemicals were ranked using the summed competition rankings of both scores, and the top 50 were selected for further consideration (Supplementary material 2.1 - Ranking). These 50 chemicals comprised various chemical classes: pharmaceuticals (n = 14), phthalates (n = 6), flame retardants (n = 6), pesticides and biocides (n = 6), phenols (n = 5), polychlorinated compounds (n = 5), parabens (n = 4), bioactive compounds (n = 3), per- and polyfluoroalkyl substances (PFASs) (n = 1).

Further selection was made based on chemical diversity in the ranked list. The chemicals included in the final selection are presented in Table 2. The in vivo and human evidence on the final list of chemicals, as shown in Table 2 and Supplementary material 3, was derived from the automatic deep research results, which were manually reviewed to ensure the quality of the evidence. The reference-checking showed that deep research generated an extensive evidence base, but with some limitations in precision. Out of 79 human and 67 animal references, 65 (82%) and 55 (82%) were checked, with 59 and 55 (90% of human, 94% of animal) verified as correct. Only verified references are included in Table 2. As Rosiglitazone (Peroxisome proliferator activated receptor γ (PPARγ) agonist) and Tributyltin (dual retinoic X receptor (RXRα) and PPARγ agonist, dysfunctional adipocytes) are often used as positive controls in adipogenesis assays, two additional chemicals in the same chemical space have been added to this list (Pioglitazone and dibutyltin).

**Fig. 3:**
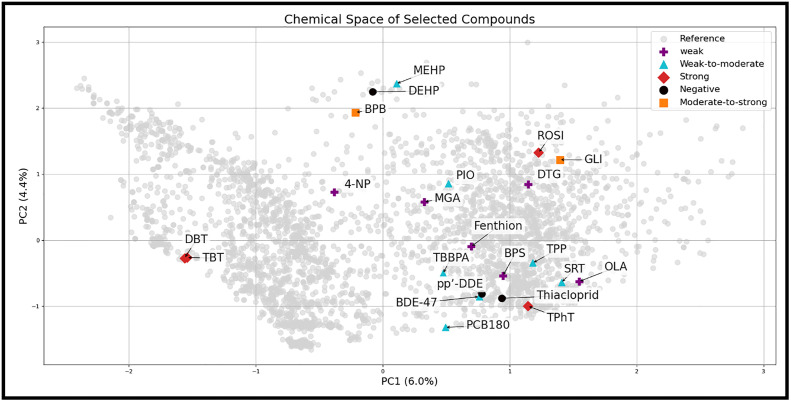
Chemical space of selected compounds based on principal component analysis (PCA) of 1024-bit radius-2 Morgan fingerprints. Grey points represent 3500 randomly selected REACH chemicals defining the background chemical structural space. Colored symbols with marker shapes indicate the selected chemicals, corresponding to evidence-based potency classifications for in vitro adipogenesis (weak, weak-to-moderate, moderate-to-strong, strong, and negative).

To evaluate the diversity of the selected compounds, we mapped their structures in a broader chemical landscape of 3500 random REACH chemicals using a PCA of Morgan fingerprints (Fig. 3). The even distribution of selected compounds across the chemical space supports the diversity of the physical chemical characteristics of the final selection and its suitability for in vitro validation (i.e., they cover a broad chemical applicability domain).

## Discussion

Here, we developed a semi-automated, evidence-based process for selecting reference chemicals for NAM validation and applied this pipeline using adipogenesis as a case study. This method combines systematic literature review, AI-assisted data extraction, scoring on both human relevance and in vitro performance, and chemical space analysis. While the ranking system generated an objective shortlist, the final chemical panel was determined through expert review of the highest-scoring candidates, ensuring biological plausibility, in vitro potency, and chemical domain.

LLMs were used for rapid abstract screening and evidence extraction, followed by a scoring system that integrates the strength of in vitro findings, cell-model relevance, and in vivo or human data. Although this process could be further automated through direct API integration (OpenAI, 2025)), our aim was to employ a cost-effective, straightforward, and transparent approach that a broad audience, including researchers without advanced expertise in data science or programming, can easily reproduce. By relying on widely accessible databases, structured literature searches, and straightforward scoring criteria, we ensure that the workflow remains practical, scalable, and adaptable for general use.

Compared to other tools, our semi-automated evidence-based pipeline falls between broad, unsupervised reference set mining and expert curation. For instance, (semi)automated workflows excel in efficiency, have objective curation, but can overlook nuances in endpoints and are often mechanistically anchored and have less focus on in vivo or human outcomes (Judson et al., 2019; Moreira-Filho et al., 2023). In contrast, expert-driven selection procedures provide strong *in vivo*/human relevance and feasibility screening, yet are labor-intensive, and subjective (Kubickova and Jacobs, 2023; Ozcagli et al., 2024). Our ranking system provides a structured balance, using both in vitro mechanistic and in vivo relevance scores to generate an unbiased shortlist. The inclusion of LLM-based Deep Research adds a broad evidence overview, improving endpoint coverage while preserving transparency. Our case study on adipogenesis allowed us to work from in vitro data first, however with other endpoints it might be more appropriate to first select chemicals based on their adverse outcome in vivo rather than in vitro.

To assess the effectiveness of our pipeline, we used a case that could be directly compared to a previously expert-oriented chemical selection, which implemented a rigorous, criteria-based chemical selection process to support the development of human PPARα and PPARγ transactivation assays, as well as white adipose tissue (WAT) adipogenesis test methods (Ozcagli et al., 2024). Our final list of 50 candidate chemicals, represents a variety of chemical classes and includes six of the eight positive compounds selected by Ozcagli et al. (2024), i.e. PFOA, TPP, TBBPA, MEHP, Rosiglitazone and TBT. Two chemicals, GW3965 and Fludioxonil, were not present in our list. GW3965, an LXR agonist primarily studied for its role in regulating lipogenic gene expression, has no direct effect on adipogenesis (Hummasti et al., 2004). Although fludioxonil has been reported to induce adipogenesis (Janesick et al., 2016), this chemical was missed as it was not mentioned in abstract/title of the initial 11,648 screened publications, which is a limitation of the current pipeline. Overall, a 75% overlap with expert judgement from the Ozcagli et al. study is similar to that reported in earlier semi-automated workflows (Judson et al., 2019).

Among the negatives proposed by Ozcagli et al. (2024), TCS, TTNPB, ppDDE and Chlorpyrifos (CPF), we included ppDDE, based on generally negative results in cell assays. Yet, human and in vivo evidence does point in the direction of weight gain, which indicating different mechanisms that might lead to weight gain. We did exclude CPF, which ranked 64th in our selection due to mixed evidence, as CPF shows an increase in adipogenesis in 3T3-L1 preadipocytes (Blanco et al., 2020). TTNPB is an inhibitor of adipogenesis (Shoucri et al., 2017), which was an exclusion criterion during the selection of studies. TCS has shown a moderate increase in adipogenesis in a recent study (J. Da Zhang et al., 2024), however, most reports indicate inhibitory effects (Guo et al., 2012; Schmid et al., 2005).

Some chemicals like BPA and PFOA proved difficult to classify due to heterogenous data. BPA was the highest-ranked, yet weakly active, chemical in our list, and it was indicated by Ozcagli et al. (2024) as a negative for the hPPARα and hPPARγ transactivation assays. There is abundant evidence on in vitro adipogenesis with BPA, although the data is inconsistent. BPA has been reported to have no effect on adipogenesis in humans or mouse MSCs at concentrations ranging from 1 nM to 100 µM (Chamorro-García et al., 2012). Meanwhile, other studies suggest it can increase adipogenesis (Dong et al., 2018; Strong et al., 2016). Inconsistency that has been demonstrated by Ren et al. (2025), who show that BPA has poor reproducibility across technical replicates and cell batches, reporting levels of lipid accumulation at 100 µM just slightly above baseline (Ren et al., 2025). Similarly, Ren et al. (2025) also reported inconsistent results for PFOA (ranked 8th in our list), which Ozcagli et al. (2024) had classified as a weak inducer of adipogenesis.

Discrepancies in assay outcomes might arise from differences in cell culture protocols, such as variations in differentiation cocktails, media composition, and cell batches. This is particularly evident with weak inducers like BPA, which we found difficult to classify due to highly heterogeneous data. Although weak inducers are an important classifier in validation studies, outcomes could be impaired by chemicals producing heterogenous data when assessing assay robustness between different laboratories. Due to these inconsistencies in the standardized hMSC model, both BPA and PFOA were excluded from our final list of candidate chemicals. Of note, our search was performed at the beginning of 2025; additional recent publications on BPA and alternatives indicate that BPA has more evidence for a positive chemical (Crosthwait et al., 2025; Flores Gomez et al., 2026).

Our selection identifies additional positive compounds compared to Ozcagli et al., 2024, including TPT, DBT (strong), Butylparaben and Glibenclamide (moderate to strong), Pioglitazone, BDE-47, Sertraline, PCB180 (weak to moderate), BPS, Olanzapine, Dolutegravir, 4-NP, Megestrol acetate, and Fenthion (weak). Thiacloprid and DEHP were included as negatives. In contrast to BPA, BPS was selected for inclusion in the final list because it shows a more robust adipogenic effect across studies. While TBT and Rosiglitazone remain well-established positive controls in adipogenesis assays, we propose DBT and Pioglitazone as mechanistic alternatives representing the organotin and thiazolidinedione classes, respectively. The inclusion of pharmaceuticals such as Dolutegravir, Sertraline, and Olanzapine broadens the chemical domain to encompass metabolic disruption linked to drug exposure. The chemical-space analysis confirmed that the final set of reference compounds achieved a wide range of structural diversity, ensuring a broad chemical applicability domain.

This chemical diversity is also exemplified by differences in mechanisms of action. Although our literature search excluded studies only focused on receptor activation, but on phenotypic selection instead, the selected compounds capture crucial mechanisms, such as PPARγ (thiazolidinediones), RXRα (organotins), and glucocorticoid receptor (megestrol acetate), all of which are associated with adipogenic effects and increased body weight or fat mass in humans and animals.

A key limitation of chemical selection remains the scarcity of confirmed negative controls. High-quality negative data are often scarce, leading to challenges in assay validation and accuracy assessment (Browne et al., 2019). In our model, thiacloprid was identified among lower-ranked candidates as a plausible negative based on weak in vitro and negative in vivo associations, while DEHP, although classified as negative, ranked higher due to frequent inclusion in phthalate studies. Additional negative controls commonly used in validation studies, such as d-mannitol, could also be considered to strengthen assay performance (Martin et al., 2022).

Several limitations of this study were identified. The first limitation is the scarcity of data from in vivo and human studies. This is because no regulatory accepted testing guidelines exist for metabolism disruption, and the measurement of adipose related endpoints is often not included in the obesity literature. Adding additional endpoints in cruuent OECD testing guidelines would enhance further chemical selection procedures (Beausoleil et al., 2026).

Despite careful search design, some studies may have been missed due to incomplete or inconsistent Emtree indexing or alternative terminology (Caponio et al., 2025). Furthermore, our initial screening was based solely on title and abstract, which might have masked adipogenic chemicals in the full text. Indeed, in our assessment, we missed Fludioxonil as an adipogenic chemical, as it was not mentioned in the abstracts but might be present in the full text. As LLM capabilities and open access policies advance future iterations of this workflow will increasingly minimize such omissions.

Additionally, AI-assisted extraction substantially improved efficiency, but it remained dependent on the clarity and quality of the reporting. GPT-4 performed best with well-structured abstracts, highlighting the ongoing need for standardized reporting. Although GPTo3 Deep Research enhanced literature retrieval, it occasionally produced citation inaccuracies or included studies on chemical mixtures when only single-compound data were relevant. Furthermore, we noticed that for some chemicals not all relevant studies were found by deep research. For instance with TPP, a recent weight of evidence approach identified 2 other studies, that were not found by deep research (Beausoleil et al., 2024). For ROSI, a large number of animal studies show increased fat or body weight, though only were included by deep research. Nevertheless, both TPP and ROSI were assessed as positive and although these limitations are recognized, the selection approach still surpasses expert judgement in terms of efficiency and accuracy. The limitation and additional minor inconsistencies such as incorrect citation metadata or reliance on abstracts and conference summaries with limited verifiability will further improve as LLM models evolve.

A further limitation was the difficulty in retrieving grey literature from regulatory agencies, such as reports and dossiers from the EPA, EFSA, ECHA, or OECD. These documents are often inconsistently indexed, difficult to access, or formatted in ways that hinder both automated text mining and manual review. Improving accessibility and standardizing metadata for such regulatory evidence would greatly strengthen future AI-assisted workflows and facilitate broader transparency in NAM validation.

## Conclusion

In conclusion, the workflow developed here provides a transparent and reproducible framework for semi-automated chemical selection to support NAM validation. By integrating AI-assisted evidence synthesis with systematic review principles, it improves efficiency and traceability while maintaining expert oversight. Deep Research outputs added in vivo and human relevance, effectively anchoring mechanistic in vitro findings to phenotypic outcomes. The final selection of reference chemicals was based on expert evaluation of the ranked list, combining algorithmic prioritization with scientific judgment to ensure relevance and feasibility. Although manual curation remains necessary, the workflow markedly reduces time and subjectivity compared with fully manual approaches. Importantly, this process should be seen as a living framework that will continue to evolve with advances in large language models, data accessibility, and regulatory harmonization, providing a scalable foundation for unbiased chemical-selection procedures across diverse toxicological endpoints.

## AI statement

This work used GTP (OpenAI) for data extraction and in vivo evidence. GPT-o3 was used for initial prescreening of in vivo data, GPT-o4 for data extraction and deep research for the final selected chemical for an in depth assessment of in vivo evidence. PICO Portal was used for systematically screen titles and abstracts during article selection.

## Funding

This project was funded by the Dutch Ministry of Infrastructure and Water Management (31202724).

## CRediT authorship contribution statement

**Hana C.M. Farnezi:** Writing – original draft, Visualization, Methodology, Formal analysis, Data curation, Conceptualization. **Sander B.I. Lentz:** Writing – review & editing, Methodology, Data curation. **Juliette Legler:** Writing – review & editing, Funding acquisition, Conceptualization. **Jorke H. Kamstra:** Writing – review & editing, Writing – original draft, Project administration, Formal analysis, Data curation, Conceptualization.

## Declaration of competing interest

The authors declare that they have no known competing financial interests or personal relationships that could have appeared to influence the work reported in this paper.

## Data Availability

No data was used for the research described in the article.

## Appendix Supplementary materials

Image, application 1

Image, application 2

Image, application 3

Image, application 4
