# Supplementary material for: Semi-automated, evidence-based workflow for selection of reference chemicals for the validation of NAMs: a case study with the adipogenesis assay
Source: NAM J. 2026 Jul 8;2:100112. doi: 10.1016/j.namjnl.2026.100112 (PMC13380221; doi:10.1016/j.namjnl.2026.100112)
Supplement: Supplementary file 1 [file mmc1.docx]

**Supplementary Material**

**Word document**

Supplementary material 1: GPT PROMPT 1: Deep research prompt for *in vivo* evidence for all chemicals

Supplementary material 1: GPT PROMPT 2: GPT prompt for *in vitro* data extraction from PDFs

Supplementary material 1: GPT PROMPT 3: Deep Research prompt for *in vivo* evidence of selected chemicals

**Excel document 1**

Supplementary material 2.1: Full list of chemicals and cell models extracted from abstracts.

Supplementary material 2.2: *In vivo* and *in vitro* ranking

**Excel document 2**

Supplementary material 2.3: *In vitro* data from included studies

**PDF**

Supplementary material 3.1: Deep Research results for *in vivo* evidence of all identified chemicals

Supplementary material 3.2: Deep Research results for *in vivo* evidence of selected chemicals

**GPT PROMPTS**

**GPT PROMPT 1**

1. ROLE

You are an expert systematic review assistant.

2. RESEARCH QUESTION

Which chemicals in the uploaded table can induce obesity or adiposity in vivo?

3. ELIGIBILITY & SCOPE

Population: Humans: observational or clinical studies; Laboratory animals: single-generation, direct-exposure experiments.

Exposure: Each chemical listed under “PREFERRED_NAME” (see table)

Comparator: Any

Outcomes: BMI, body weight, body-fat %, fat mass, waist/hip measures, fat-pad weights, adipocyte size/number

• Sources: PubMed, Embase, Scopus, plus grey literature from EPA IRIS, EFSA, ECHA, ATSDR, WHO, OECD, FDA dockets

• Language: English

• Exclude: in-vitro work, mechanistic-only papers, multigenerational animal studies, reviews, editorials.

4. METHOD

1. Draft search strings.

2. Retrieve and de-duplicate records.

3. Extract the following fields:

- Citation (author + year) - Species & design - Sample size

- Exposure details (dose, route, duration) - Relevant outcomes & effect size/direction

- Study limitations

5. For **each chemical**:

a. Present a results table

b. Provide a concise evidence conclusion (“Strong / Limited / Inadequate / No evidence”).

6. Create one **summary table** (chemical | human evidence | animal evidence).

5. OUTPUT FORMATS

Per-chemical table (markdown):

| Study ID | Species | Design | N | Exposure | Outcome(s) | Effect |

Summary table (markdown):

| Chemical | Human evidence (Strong/Limited/None) | Animal evidence (Strong/Limited/None) |

**GPT PROMPT 2**

Extract from these PDFs all relevant data on the effects of "CHEMICAL NAME" on adipogenesis. Create a structured table with the following columns:

1. Reference (author, year (Smith et al. 2025))

2. Cell model (e.g., MSC, 3T3-L1, etc.)

3. Effect (increase or decrease in adipogenesis)

4. Lowest effective dose (dose with a statistically significant effect, p < 0.05)

5. Effect size at the lowest dose (fold change of CHEMICAL vs. control, if not reported, check available charts)

6. Conclusion (brief summary of effects at other tested doses)

Make sure to: Check charts, figures, and tables for quantitative data. Include all relevant references. If multiple doses or cell types are used, list each entry separately. Always follow the same table structure.

**GPT PROMPT 3**

Conduct a comprehensive deep search to determine if exposure to “CHEMICAL NAME (CAS nr)” increases adiposity or obesity endpoints.

RESEARCH QUESTION: Which chemicals are able to induce adipogenesis, adiposity, or obesity in vivo?

ELIGIBLE EVIDENCE

• Humans: observational or clinical studies.

• Laboratory animals: direct-exposure experiments.

• Grey literature from major agencies (EPA IRIS, EFSA, ECHA, ATSDR, WHO, OECD, FDA dockets).

• Only include documents in English

• EXCLUDE in-vitro studies, mechanistic-only reports, multigenerational animal studies, reviews, and editorials.

OUTPUT

HUMAN RESULTS

**Table 1**: Reference | Design | N | Exposure metric | Adiposity effect | Important Notes.

ANIMAL RESULTS

**Table 2**: Reference | Species | Design | Dose(s) | Adiposity effect | Important Notes.

CONCLUSION

Strength of evidence (strong / moderate / limited / inconsistent / none) for humans and animals separately.
