# Supplementary material for: Semi-automated, evidence-based workflow for selection of reference chemicals for the validation of NAMs: a case study with the adipogenesis assay
Source: NAM J. 2026 Jul 8;2:100112. doi: 10.1016/j.namjnl.2026.100112 (PMC13380221; doi:10.1016/j.namjnl.2026.100112)
Supplement: Supplementary file 4 [file mmc4.pdf]

Supplementary material 3.2

4-Nonylphenol

Human Results

Table 1: Epidemiological studies on 4-nonylphenol exposure and adiposity

Reference	Design	N	Exposure Metric	Adiposity Effect	p/CI	Notes
Lopez-Espinosa et al. (2009)	Cross-sectional (Spain)	20	NP in adipose tissue (ng/g)	Positive correlation: higher NP levels in fat with higher BMI	*p* = 0.041	Non-occupational adult women; NP detected in 100% of samples.
Seo et al. (2019)	Cross-sectional (Korean children)	204 (105 obese, 99 control)	Serum NP (ng/mL)	Higher NP associated with greater weight, BMI percentile, and fat mass (trend significant); top exposure quartile had higher odds of obesity	OR = 2.47 (95% CI 1.03–5.92) (highest vs lowest quartile); *P*<0.05 (trend)	Ages 6–14; association particularly observed in girls.
Sung et al. (2006)	Cross-sectional (Taiwan couples)	90 (45 couples)	Urinary NP (µg/g creatinine)	Obesity associated with elevated NP: highest BMI tertile had greatly increased odds of high NP levels	OR = 13.4 (95% CI 1.11–161)	Infertility clinic patients; spouses showed similar NP exposures.
Park et al. (2017)	Cross-sectional (Korean adults)	1865	Urinary NP (ng/mL)	No significant association between NP levels and BMI category (no trend in NP by BMI)	*p* = 0.84 (overall)	National biomonitoring survey of adults; NP detected in ~83%.

Animal Results

Table 2: Experimental studies on 4-nonylphenol exposure and adiposity

Reference	Species	Design	Dose(s)	Adiposity Effect	p/CI	Notes
Hao et al. (2012)	Mouse	Perinatal exposure (gestation day 12 to lactation day 7)	0.05, 0.25, 0.5 mg/kg/day (oral)	Increased offspring body weight and fat mass at postnatal day 60	*p*<0.05 (dose groups vs. control)	Dams treated by gavage; offspring also had higher cholesterol and glucose.
Yu et al. (2017)	Rat	90-day oral exposure	180 mg/kg/day (gavage)	Increased body weight, fat weight,	*p*<0.05 (NP vs. control)	Normal diet group; NP

Reference	Species	Design	Dose(s)	Adiposity Effect	p/CI	Notes
		(young males)		and fat mass index vs. controls		exposure also induced fatty liver changes.
Yu et al. (2020)	Rat	180-day chronic exposure (adults)	0.02, 0.2, 2.0 μg/kg/day (gavage)	Dose-dependent increase in body fat weight and fat/body weight ratio ("fat coefficient"); significant body weight gain by week 26	p<0.001 (fat weight/fat coefficient ANOVA); p = 0.023 (body weight at 26 weeks)	Low-dose, long-term exposure; elevated serum lipids and adipogenic markers observed.

Conclusion

In humans, the evidence linking 4-nonylphenol exposure to increased adiposity is **inconsistent/limited**. A few cross-sectional studies report positive associations with obesity (especially in children and specific subgroups)\, but at least one large adult study found no significant relationship. In contrast, animal studies provide **strong** evidence that 4-nonylphenol can induce adiposity: multiple rodent experiments consistently show increased body weight and fat accumulation after exposure.

Strength of evidence: Humans – *limited/inconsistent*; Animals – *strong*.

References

- Hao, C.-J., Cheng, X.-J., Xia, H.-F., & Ma, X. (2012). *The endocrine disruptor 4-nonylphenol promotes adipocyte differentiation and induces obesity in mice*. Cell Physiology and Biochemistry, 30(2), 382–394[10].

- Lopez-Espinosa, M. J., Freire, C., Arrebola, J. P., Navea, N., Taoufiki, J., Fernández, M. F., … & Olea, N. (2009). *Nonylphenol and octylphenol in adipose tissue of women in Southern Spain*. Chemosphere, 76(6), 847–852[1].

- Park, H., Lee, J. H., Kim, K., et al. (2017). *Urinary levels of 4-nonylphenol and 4-t-octylphenol in a representative sample of the Korean adult population*. International Journal of Environmental Research and Public Health, 14(8), 932[8].

- Seo, M. Y., Kim, S.-H., & Park, M. J. (2019). *Serum nonylphenol and obesity in children and adolescents*. **ESPE Abstracts**, 92, P1-203[2].

- Sung, F. C., Lu, C.-Y., Chang, C.-H., Lin, R.-S., Torng, P.-L., Mao, I.-F., & Chen, M.-L. (2006). *Influence of life and diet styles on nonylphenol exposure*. Epidemiology, 17(6) (Supplement), S466–S467[6].

- Yu, J., Yang, X., Luo, Y., Yang, X., Yang, M., Yang, J., … & Xu, J. (2017). *Adverse effects of chronic exposure to nonylphenol on non-alcoholic fatty liver disease in male rats*. PLoS ONE, 12(7), e0180218[13].

- Yu, J., Li, W., Tang, L., Luo, Y., & Xu, J. (2020). *In vivo and in vitro effects of chronic exposure to nonylphenol on lipid metabolism*. Environmental Sciences Europe, 32, 87[15][17].

BDE-47 Exposure and Adiposity Outcomes

Human Results

Table 1. Studies of BDE-47 exposure and adiposity outcomes in humans. (N = sample size; GWG = gestational weight gain; BMIz = body mass index z-score.)

Reference (Year)	Design (Population)	N	Exposure Metric	Adiposity Outcome Effect	p / 95% CI	Notes
Vuong *et al.* (2016)	Prospective birth cohort (USA)	318	Maternal serum PBDEs (prenatal)	Higher BDE-153 (and ΣPBDE) → *lower* child BMI & waist (ages 2–8)	$p < 0.05$	Inverse association (no positive link to obesity).
Agay-Shay *et al.* (2015)	Prospective birth cohort (Spain)	391	Maternal serum PBDEs (prenatal)	No significant association with child BMI at 7 years (β < 0)	$p > 0.1$	Multi-pollutant analysis; PBDE–BMI link was null.
Erkin-Cakmak *et al.* (2015)	Prospective birth cohort (USA)	272	Maternal & child serum PBDEs	No overall association with BMI at 7; in girls, higher BDE-153 → *lower* BMIz	$p < 0.05$ (girls)	CHAMACOS study; sex-specific inverse effect noted.
Hoepner *et al.* (2022)	Prospective birth cohort (USA)	289	Cord plasma BDE-47, -99, -100, -153	No association with childhood BMI trajectories (ages 5–14)	$p > 0.05$	High vs. low prenatal BDE-47 showed no BMIz difference.
Wang *et al.* (2024)	Prospective pregnancy cohort (USA)	2,449	1st trimester plasma BDE-47	Among obese mothers: 1 SD ↑ BDE-47 → +1.87 kg total GWG (95% CI 0.39–3.35)	$p = 0.01$	No GWG effect in non-obese women; BDE-47 consistently ↑ GWG in obese group.

Animal Results

Table 2. Experimental animal studies of BDE-47 exposure and adiposity outcomes. (GD = gestational day; PND = postnatal day; HFD = high-fat diet; WAT = white adipose tissue.)

Reference (Year)	Species (Sex)	Design (Exposure Timing)	Dose(s)	Adiposity Effect	p / 95% CI	Notes
Suvorov *et al.* (2009)	Rat (M/F)	Perinatal (GD15–PND20, in	0, 0.002, 0.2 mg/kg iv every 5d	↑ Offspring body weight & length (both sexes) vs. control	$p < 0.05$	Low-dose BDE-47 increased pup growth; elevated

Reference (Year)	Species (Sex)	Design (Exposure Timing)	Dose(s)	Adiposity Effect	p / 95% CI	Notes
		utero & lactational)				IGF-1 and glucose uptake in males.
Strunz *et al.* (2024)	Mouse (M/F)	Perinatal (gestation & lactation exposure)	Low dose (~human TDI estimate)	↑ Adult body weight and fat mass in female offspring vs. control; no weight change in males	$p < 0.01$ (females)	Maternal low-dose BDE-47 → female offspring overweight; both sexes had impaired glucose tolerance.
Wang *et al.* (2018)	Mouse (M)	Perinatal + post-weaning HFD (in utero, lactational → adult HFD)	0, 0.002, 0.2 mg/kg gavage (GD6–PND21)	Exacerbated HFD-induced obesity in offspring: ↑ body weight, adiposity, hepatic steatosis vs. HFD only	$p < 0.05$	Early-life BDE-47 exposure **promoted obesity** under high-fat diet; altered gut microbiome and lipid metabolism.
Yang *et al.* (2022)	Mouse (M)	Adult exposure + diet (continuous dosing during diet)	0 or BDE-47 (dose not stated, 12 wks)	Under HFD: ↑ WAT weight gain, adipocyte hypertrophy, lipid accumulation vs. HFD control (*obesity*); under low-fat diet: no effect	$p < 0.05$ (HFD group)	BDE-47 + HFD caused WAT dysfunction & inflammation, leading to **severe obesity**. No obesogenic effect on low-fat diet.
Kenerson *et al.* (2015)	Mouse (M)	Early postnatal (weaning to young adult)	1 mg/kg/day gavage ×6 weeks	No change in body, liver, or fat weight vs. vehicle control (no adiposity increase)	$p > 0.05$	No obesity effect in wild-type or transgenic mice; BDE-47 induced insulin resistance only in a susceptible genotype.

Conclusion

In **human studies**, the overall evidence that BDE-47 exposure increases adiposity is *limited and inconsistent*. Most epidemiological studies report no association or even inverse correlations between prenatal BDE-47 (or PBDE mixture) levels and child adiposity measures. Only one study found a positive association (higher gestational weight gain in already-obese pregnant women with higher BDE-47), and no clear link to obesity has been observed in the general population. In **animal studies**, there is **strong evidence** that BDE-47 can act as an obesogen under certain conditions. Multiple rodent experiments demonstrate that perinatal exposure to low doses of BDE-47 leads to increased offspring body weight, fat accumulation, and worsened diet-induced obesity. These effects are especially pronounced with developmental exposure and high-fat diet, though one postnatal study showed no weight change under normal diet. Overall, BDE-47 shows *obesogenic* effects in animals

(strong evidence), whereas in humans the evidence of increased adiposity is *limited/inconsistent*.

References:

1. Vuong, A. M., Braun, J. M., Sjödin, A., Webster, G. M., Yolton, K., Lanphear, B. P., & Chen, A. (2016). Prenatal Polybrominated Diphenyl Ether Exposure and Body Mass Index in Children up to 8 Years of Age. *Environmental Health Perspectives, 124*(11), 1891–1897. DOI: 10.1289/EHP205[1]

2. Agay-Shay, K., Martinez, D., Valvi, D., García-Esteban, R., Basagaña, X., Robinson, O., … & Vrijheid, M. (2015). Exposure to endocrine-disrupting chemicals during pregnancy and weight at 7 years of age: a multi-pollutant approach. *Environmental Health Perspectives, 123*(10), 1030–1037. DOI: 10.1289/ehp.1409049[1]

3. Erkin-Cakmak, A., Harley, K. G., Chevrier, J., Bradman, A., Kogut, K., Huen, K., … & Eskenazi, B. (2015). In utero and childhood polybrominated diphenyl ether exposures and body mass at age 7 years: The CHAMACOS study. *Environmental Health Perspectives, 123*(7), 636–642. DOI: 10.1289/ehp.1408417.

4. Hoepner, L. A., Gallagher, D., Baccarelli, A. A., Zhang, B., Goldsmith, J., Peterson, M., … & Herbstman, J. B. (2022). Prenatal exposure to polybrominated diphenyl ethers and BMI z-scores from 5 to 14 years. *Environmental Health, 21*(1), 82. DOI: 10.1186/s12940-022-00893-5[2]

5. Wang, Z., Williams, P. L., Bellavia, A., Wylie, B. J., Kannan, K., Bloom, M. S., … & James-Todd, T. (2024). Polybrominated diphenyl ethers and gestational weight gain: a multi-center prospective cohort study. *BJOG, 131*(11), 1484–1494. DOI: 10.1111/1471-0528.17860[3]

6. Strunz, S., Strachan, R., Bauer, M., Zenclussen, A. C., Leppert, B., Junge, K. M., & Polte, T. (2024). Maternal exposure to low-dose BDE-47 induced weight gain and impaired insulin sensitivity in the offspring. *International Journal of Molecular Sciences, 25*(16), 8620. DOI: 10.3390/ijms25168620[6]

7. Wang, D., Yan, J., Teng, M., Yan, S., Zhou, Z., & Zhu, W. (2018). In utero and lactational exposure to BDE-47 promotes obesity development in mouse offspring fed a high-fat diet: impaired lipid metabolism and intestinal dysbiosis. *Archives of Toxicology, 92*(5), 1847–1860. DOI: 10.1007/s00204-018-2177-0[8]

8. Yang, C., Wei, J., Cao, G., & Cai, Z. (2022). Lipid metabolism dysfunction and toxicity of BDE-47 exposure in white adipose tissue revealed by integration of lipidomics and metabolomics. *Science of the Total Environment, 806*, 150350. DOI: 10.1016/j.scitotenv.2021.150350[9]

9. Kenerson, H. L., Subramanian, S., Wang, S. A., Kazami, M., Stapleton, H. M., & Yeung, R. S. (2015). Polybrominated diphenyl ether congener BDE-47 impairs insulin sensitivity in mice with liver-specific Pten deficiency. *BMC Obesity, 2*(1), 3. DOI: 10.1186/s40608-014-0031-3[12]

10. Suvorov, A., Battista, M. C., & Takser, L. (2009). Perinatal exposure to low-dose 2,2′,4,4′-tetrabromodiphenyl ether (BDE-47) affects growth in rat offspring: what is the role of IGF-1?. *Toxicology, 260*(1–3), 126–131. DOI: 10.1016/j.tox.2009.03.018[5]

Bis(2-ethylhexyl) phthalate (DEHP)

Human Results

Table 1: Summary of human studies on DEHP exposure and adiposity

Reference (APA)	Design	N	Exposure Metric	Adiposity Effect	p / 95% CI	Notes
Deodati et al. (2024)	Case-control (children, obese vs normal weight)	122	Urinary DEHP metabolite levels (MEHP, MEHHP, MEOHP)	Obese girls had higher DEHP metabolic ratio (MEHP fraction) vs controls; DEHP metabolites correlated with higher leptin in obese girls	$p < 0.05$ (ratio); $p < 0.03$ (leptin)	Effect observed in girls only; no significant differences in boys (BPA showed stronger associations)
Wen et al. (2024)	Prospective birth cohort (18-year follow-up)	208	Maternal prenatal urinary ∑DEHP (sum of metabolites)	Higher prenatal DEHP associated with children being in a **stable-high BMI trajectory** through adolescence	OR for high-BMI trajectory increased (significant)	Prenatal DEHP exposure doubled increased obesity risk up to age 18 (Taiwan cohort)
Desalegn et al. (2024)	Cross-sectional (European multi-country HBM4EU study)	2,045†	Urinary ∑DEHP metabolite concentration	**No positive association** overall; in adolescent females, higher ∑DEHP was **inversely** associated with BMI z-score (lower BMI at higher DEHP)	$\beta = -0.08$ (–0.14, –0.02) in girls	Age- and sex-specific effects: DEHP tied to slightly lower adolescent BMI in girls; no adiposity increase observed
Zhang et al. (2014)	Cross-sectional (Chinese children 8–13y)	493	Urinary phthalate monoesters (incl. DEHP metabolites)	**Sex-specific**: In boys, non-DEHP phthalates (MBP) positively associated with obesity; in girls, higher DEHP metabolite levels were **negatively** associated with obesity prevalence	$p < 0.05$ (sex-interactions)	Observed inverse relationship between DEHP (MEHP, MEHHP, ∑DEHP) and obesity in girls; suggests potential sex difference in effect
Buser et al. (2014)	Cross-sectional (NHANES	4,369	Urinary DEHP metabolites (MEHP,	In **adults**, higher DEHP metabolite levels associated	$p < 0.05$ (adults)	Among adults, DEHP metabolites were

Reference (APA)	Design	N	Exposure Metric	Adiposity Effect	p / 95% CI	Notes
	2007–2010, USA)		MEHHP, MEOHP, MECPP)	with greater obesity odds (both sexes) ; in **children**, DEHP showed no significant effect on obesity (LMW phthalates affected boys)		linked to higher obesity prevalence (esp. in females and older males); no clear DEHP–obesity link in youth (only other phthalates in subgroups)
Trasande et al. (2013)	Cross-sectional (NHANES 2003–2008, children 6–19y)	2,884	Urinary DEHP metabolite sum vs BMI z and obesity	**Null overall** – No significant association between DEHP metabolites and BMI or obesity in any race/ethnic subgroup after adjustment; (by contrast, LMW phthalates associated with obesity in Black children)	–	No evidence that DEHP exposure increased odds of childhood obesity in this nationally representative sample (only certain non-DEHP phthalates showed associations)
Yang et al. (2017)	Prospective cohort (Mexico; prenatal & childhood exposure)	249	Maternal 3rd trimester and child urinary DEHP metabolites (MEHP, etc.)	**No obesogenic effect** – Higher childhood DEHP metabolite (MEHP) was linked to *lower* adiposity (smaller waist circumference and skinfold thickness in prepubertal children)	$\beta = -1.85$ cm waist per ln-MEHP, 95% CI –3.36, –0.35	Identified inverse associations (and no positive links) for DEHP; suggests potential confounding or reverse causation (e.g. obese children excrete less MEHP)

Animal Results

Table 2: Summary of in vivo animal studies on DEHP exposure and adiposity

Reference (APA)	Species	Design (Exposure)	Dose(s)	Adiposity Effect	p / 95% CI	Notes
Klöting et al. (2015)	Mouse (129S6 strain)	10-week oral exposure in	0.05 mg/kg/day	**Increased adiposity:** Treated mice	$p < 0.05$ (vs control)	Low-dose, chronic exposure; effect significant in

Reference (APA)	Species	Design (Exposure)	Dose(s)	Adiposity Effect	p / 95% CI	Notes
		adulthood (dietary)		(females) gained weight more rapidly and had higher body fat percentage than controls		females only (obesity-resistant strain)
Hao et al. (2013)	Mouse	Perinatal exposure (gestation & lactation)	*Not reported* (environmentally relevant range)	**Increased adiposity:** Offspring exhibited higher body weight and fat accumulation in later life ("induced obesity")	$p < 0.05$ (reported)	Exposure during development led to increased adult adiposity in mice (supporting obesogen hypothesis)
Gu et al. (2016)	Mouse (C57BL/6J)	Prenatal exposure (maternal gavage GD 1–19)	0.05 mg/kg/day	**Increased visceral fat:** Offspring had significantly heavier visceral (gonadal) fat pads than controls at 9 weeks of age; no change in overall body weight	$p < 0.05$	Low-dose in utero exposure (below toxicity threshold) increased fat depot mass and metabolic markers (↑ leptin, insulin) in offspring
Schmidt et al. (2012)	Mouse (C3H/N)	8-week dietary exposure (young females)	**Low:** 0.05% diet; **High:** 0.5% diet	**Dose-dependent:** *Low* dose DEHP led to ↑ **body weight and visceral fat** in adult female mice, whereas *high* dose impaired fertility but **did not increase fat**	$p < 0.05$ (low-dose vs control)	"Environmental" low dose caused weight gain and fat depot enlargement; high dose had toxic effects (reproductive) without obesogenic effect (non-monotonic response)
Martinelli et al. (2010)	Rat	4-week dietary exposure	2% w/w in feed (very high dose)	**Decreased adiposity:** Treated rats showed	$p < 0.05$ (vs control)	High-dose DEHP (PPARα activator levels) increased fat metabolism,

Reference (APA)	Species	Design (Exposure)	Dose(s)	Adiposity Effect	p / 95% CI	Notes
		(adult males)		**reduced** epididymal fat weight and smaller adipocytes compared to controls		leading to fat loss (opposite effect at pharmacological dose)

Conclusion

In **humans**, the evidence linking DEHP exposure to increased adiposity is **inconsistent**. Some observational studies (especially prenatal exposure cohorts) suggest a positive association between DEHP and higher childhood BMI, but many cross-sectional studies show null or even inverse relationships, often varying by sex and age. Overall, human data provide only **limited and inconsistent** support for DEHP as an obesogen.

In **animals**, there is **moderate evidence** that DEHP can increase adiposity under certain conditions. Multiple rodent studies, particularly with perinatal or chronic low-dose exposures, have demonstrated increased body weight or fat mass in exposed animals. Meta-analysis confirms a small but significant increase in fat weight with early-life DEHP exposure in rodents. However, effects are not uniform across doses and sexes – e.g., very high doses can produce opposite effects (weight/fat loss) and some studies report sex-specific or no effects. Taken together, animal findings support an **adipogenic effect of DEHP** (especially at low environmentally relevant doses), bolstering the biological plausibility, but with **some inconsistencies** at extreme exposures.

References (APA):

- Buser, M. C., Murray, H. E., & Scinicariello, F. (2014). Age and sex differences in childhood and adulthood obesity association with phthalates: analyses of NHANES 2007–2010. *International Journal of Hygiene and Environmental Health, 217*(6), 687–694. DOI: 10.1016/j.ijheh.2014.02.005

- Deodati, A., Bottaro, G., Germani, D., Carli, F., Tait, S., Busani, L., … Cianfarani, S. (2024). Urinary Bisphenol A and Bis(2-ethylhexyl) phthalate metabolite concentrations in children with obesity: a case-control study. *Hormone Research in Paediatrics, 97*(4), 388–396. DOI: 10.1159/000535305

- Gu, H., Liu, Y., Wang, W., Ding, L., Teng, W., & Liu, L. (2016). In utero exposure to di-(2-ethylhexyl) phthalate induces metabolic disorder and increases fat accumulation in visceral depots of C57BL/6J mice offspring. *Experimental and Therapeutic Medicine, 12*(6), 3806–3812. DOI: 10.3892/etm.2016.3820

- Hao, C., Cheng, X., Guo, J., Xia, H., & Ma, X. (2013). Perinatal exposure to diethyl-hexyl phthalate induces obesity in mice. *Frontiers in Bioscience (Elite Edition), 5*(2), 725–733. DOI: 10.2741/E653

- Klöting, N., Hesselbarth, N., Gericke, M., Kunath, A., Biemann, R., Chakaroun, R., … Blüher, M. (2015). Di-(2-ethylhexyl)-phthalate (DEHP) causes impaired adipocyte function and alters

serum metabolites in mice. *PLoS ONE, 10*(12), e0143190. DOI: 10.1371/journal.pone.0143190

- Martinelli, M. I., Mocchiutti, N. O., & Bernal, C. A. (2010). Effect of di(2-ethylhexyl) phthalate on lipolysis and lipoprotein lipase activities in adipose tissue of rats. *Human & Experimental Toxicology, 29*(9), 739–745. DOI: 10.1177/0960327110361750

- Schmidt, J. S., Schaedlich, K., Fiandanese, N., Pocar, P., & Fischer, B. (2012). Effects of di(2-ethylhexyl) phthalate on female fertility and adipogenesis in C3H/N mice. *Environmental Health Perspectives, 120*(8), 1123–1129. DOI: 10.1289/ehp.1104013

- Trasande, L., Attina, T. M., Sathyanarayana, S., Spanier, A. J., Blustein, J., & others. (2013). Race/Ethnicity-specific associations of urinary phthalates with childhood body mass in a nationally representative sample. *Environmental Health Perspectives, 121*(4), 501–506. DOI: 10.1289/ehp.1205526

- Wen, H. J., Su, P. H., Sun, C. W., Tsai, S. F., & Wang, S. L. (2024). Maternal phthalate exposure and BMI trajectory in children – an 18-year birth cohort follow-up study. *Journal of Exposure Science & Environmental Epidemiology, 34*(4), 601–609. DOI: 10.1038/s41370-024-00696-5

- Yang, T. C., Peterson, K. E., Meeker, J. D., Sánchez, B. N., Zhang, Z., Cantoral, A., … Téllez-Rojo, M. M. (2017). Bisphenol A and phthalates in utero and in childhood: association with child BMI z-score and adiposity. *Environmental Research, 156*, 326–333. DOI: 10.1016/j.envres.2017.03.038

Bisphenol S

Human Results

Several epidemiological studies have examined BPS exposure in relation to adiposity with mixed findings. Cross-sectional analyses of U.S. adults (NHANES 2013–2014, N≈1521) initially observed higher urinary BPS in obese vs. non-obese adults, but BPS was not significantly associated with obesity after adjustment (only BPA showed a significant association). In a mixture analysis of NHANES 2013–2014 adults (N=1269), Zhang et al. (2019) found BPS to be one of the chemicals most consistently linked with higher odds of obesity across multiple models. Among pregnant women in a Dutch cohort (N=1396), BPS was frequently detected but showed no correlation with pre-pregnancy obesity. In children and adolescents, BPS findings are likewise variable. A cross-sectional study of U.S. youth (NHANES 2013–2014, N=745, ages 6–17) reported no significant association of urinary BPS with general or abdominal obesity. However, an analysis of NHANES 2013–2016 (1831 participants, ages 6–19) found that higher BPS levels were associated with increased prevalence of both general and abdominal obesity. In the HOME longitudinal birth cohort (Cincinnati, USA), childhood urinary BPS concentrations (measured at age 8) were not associated with adiposity outcomes at ages 8 or 12. In contrast, a recent prospective study in Korean children (N=561) showed that those with higher BPS exposure (upper 50th percentile) had greater adiposity: e.g. higher BMI z-scores and fat mass (β for fat mass = 0.104, P<0.001) alongside higher leptin and lower adiponectin levels compared to BPS non-detected children.

Table 1. Human studies on BPS exposure and adiposity outcomes.

Reference	Design	N	Exposure metric	Adiposity effect	*p*/CI	Notes
Liu et al. (2017)	Cross-sectional (NHANES 2013–14 adults)	1521	Urinary BPS (spot)	No significant association with obesity (BMI ≥30)	– (ns)	Obese had higher median BPS than non-obese, but adjusted OR for obesity was not significant.
Zhang et al. (2019)	Cross-sectional (NHANES 2013–14 adults)	1269	Urinary BPS (spot)	Positive association with obesity	$p<0.05$	BPS among top contributors to obesity risk in multi-chemical models.
Philips et al. (2018)	Cohort (pregnant women, Netherlands)	1396	Urinary BPS (spot)	No correlation with pre-pregnancy obesity	– (ns)	Widespread BPS detection, but no link to maternal BMI status.
Liu et al. (2019)	Cross-sectional (NHANES 2013–14 youth)	745	Urinary BPS (spot)	No association with obesity (general or abdominal)	– (ns)	BPS null; in same study BPA/BPF showed positive associations.
Jacobson et al. (2019)	Cross-sectional (NHANES 2013–16 youth)	1831	Urinary BPS (spot)	↑ General and abdominal obesity prevalence	$p<0.05$	Higher BPS tertile linked to greater odds of obesity (including central obesity).
Gajjar et al. (2022)	Prospective cohort (HOME study, USA)	212	Urinary BPS (age 8)	No association with adiposity at ages 8 or 12	– (ns)	Percent body fat and BMI not significantly related to childhood BPS exposure.
Lee et al. (2024)	Child cohort (Seoul, Korea)	561	Urinary BPS (6–8 y/o)	↑ BMI *z*-score, fat mass ($\beta=0.104$) in higher exposure group	$P<0.05$	High BPS exposure also associated with higher leptin, lower adiponectin (adverse adipokine profile).

Animal Results

Multiple in vivo studies report that BPS exposure can induce obesity-related outcomes in laboratory animals. Ivry Del Moral et al. (2016) exposed pregnant mice to BPS (0.2, 1.5, or 50 µg/kg bw/day in drinking water) from gestation through adulthood of offspring. Adult male offspring on a high-fat diet (HFD) showed significantly increased body weight ("overweight") at the two higher BPS doses, with increased fat mass, despite no change in food intake or energy expenditure. These BPS-exposed HFD mice also exhibited hyperinsulinemia, hyperleptinemia, and upregulation of adipogenic genes in adipose tissue. Meng et al. (2019) observed that perinatal BPS exposure (100 µg/kg bw/day) in mice led to adult male offspring with increased body weight, enlarged epididymal white adipose tissue, fatty liver changes, and elevated hepatic triglyceride and cholesterol content. Histologically, BPS caused significant lipid accumulation in adipose and liver, accompanied by dysregulation of genes involved in lipid and glucose metabolism. Similarly, Ahn et al. (2020) reported that prenatal BPS exposure in mice predisposed male offspring to exacerbated diet-induced obesity: when challenged with an HFD, BPS-exposed males had greater

gonadal fat pad hypertrophy and higher expression of adipogenesis markers (e.g. PPARγ, C/EBPα, aP2) compared to controls on HFD. In adult female mice, BPS can also aggravate adiposity in an estrogen-dependent manner. Wen et al. (2023) found that giving female mice an environmentally relevant BPS dose (≈125 μg/kg/day) increased susceptibility to HFD-induced obesity – BPS-treated females gained more weight on HFD and their brown adipose tissue (BAT) exhibited a "whitened" phenotype (lipid accumulation with reduced thermogenic characteristics). Notably, this effect was abolished in ovariectomized females and restored with estrogen, indicating the obesogenic action of BPS in females is mediated by estrogen signaling. While most animal studies show obesogenic effects of BPS, one short-term high-dose study in rats (50 mg/kg/day for 28 days) did not observe increased adiposity – instead, body weight gain was suppressed, likely due to general toxicity at that high dose. Overall, animal evidence largely demonstrates that BPS exposure (particularly during developmental periods or in combination with a high-fat diet) can promote greater adiposity, weight gain, and metabolic disturbances in offspring.

Table 2. Key animal studies of BPS exposure on adiposity outcomes.

Reference	Species	Design	Dose(s) & regimen	Adiposity effect	*p*/CI	Notes
Ivry Del Moral et al. (2016)	Mouse (C57BL/6)	Perinatal + chronic exposure (GD0 through 23 weeks); HFD challenge in adulthood	0.2, 1.5, 50 μg/kg bw/day (maternal drinking water)	↑ Body weight and fat mass in adult male offspring on HFD (at 1.5 & 50 μg/kg)	$p<0.05$	BPS potentiated diet-induced obesity in males; induced hyperinsulinemia, hyperleptinemia; no hyperphagia or ↓activity observed.
Meng et al. (2019)	Mouse (strain not specified)	Perinatal exposure (gestation through lactation); offspring evaluated in adulthood (normal diet)	100 μg/kg bw/day (gavage)	↑ Body weight, visceral fat (epiWAT), fatty liver in male offspring	$p<0.05$	BPS-exposed males had larger epididymal fat pads; hepatic TG and cholesterol ↑; adipose and liver showed significant lipid accumulation and inflammatory gene expression.
Ahn et al. (2020)	Mouse (ICR)	Prenatal exposure (gestation); adult offspring on HFD vs. control diet	*Dose not reported* (gestational exposure via dams)	↑ Visceral fat hypertrophy in BPS group males on HFD	$p<0.05$	Prenatal BPS predisposed males to greater gonadal WAT mass under HFD; upregulated adipogenic genes (PPARγ, aP2, etc.) in fat.
Wen et al. (2023)	Mouse (female)	Adult exposure + HFD (ovary-intact vs. ovariectomized)	125 μg/kg bw/day (administration method based	↑ Weight gain on HFD; BAT "whitening"	$p<0.01$	BPS increased adiposity and body weight in females with intact ovaries

Reference	Species	Design	Dose(s) & regimen	Adiposity effect	*p*/CI	Notes
			on human equiv. dose)	(fat accrual, loss of brown function)		on HFD; effect absent without estrogen (ovx), confirming estrogen-dependent mechanism.
Sharma & Mandal (2021)	Rat (albino)	Adult subacute exposure (28 days)	50 mg/kg bw/day (oral gavage)	**No obesogenic effect** (body weight gain ↓ vs. controls)	$p<0.05$	High-dose BPS caused weight suppression and organ toxicity rather than fat gain (indicating general toxicity at this dose).

Conclusion

Human evidence: *Inconsistent/limited.* Epidemiological findings on BPS and adiposity are mixed. Some cross-sectional studies (including U.S. NHANES analyses) and a recent child cohort in Korea suggest a positive association between BPS exposure and obesity-related measures. However, other studies – including cross-sectional and prospective cohorts – found no significant link between BPS and BMI or fat outcomes. The variability in results, often depending on age group and study design, means current human evidence for BPS increasing adiposity is limited and not yet consistent.

Animal evidence: *Moderate.* Multiple controlled animal experiments demonstrate that BPS can act as an obesogen. Developmental exposure to low-dose BPS in mice reliably increased adiposity and weight gain in offspring, especially when coupled with a high-fat diet. BPS's obesogenic effects have been observed across different labs and scenarios (perinatal and adult exposure), and involve mechanistic changes in adipose tissue (e.g. enhanced adipogenesis, lipid accumulation). While one high-dose study in rats showed no weight gain (likely due to toxicity at that dose), the overall animal data consistently indicate that BPS exposure can promote increased fat deposition and obesity-related endpoints under experimental conditions. Thus, in animals, there is moderate evidence of BPS's adiposity-increasing effects.

References (APA style):

Braun, J. M., Gajjar, P., … & Yolton, K. (2022). *Associations of mid-childhood bisphenol A and bisphenol S exposure with mid-childhood and adolescent obesity*. Environmental Epidemiology, 6(1), e196[6].

Ivry Del Moral, L., Le Corre, L., … & Chagnon, M. C. (2016). *Obesogen effects after perinatal exposure of 4,4′-sulfonyldiphenol (Bisphenol S) in C57BL/6 mice*. Toxicology, 357-358, 11–20[9][10].

Jacobson, M. H., Woodward, M., Bao, W., Liu, B., & Trasande, L. (2019). *Urinary bisphenols and obesity prevalence among U.S. children and adolescents*. Journal of the Endocrine Society, 3(9), 1715–1726[5].

Lee, H. J., Lee, Y. J., … & Lee, Y. A. (2024). *Relationship of bisphenol A substitutes bisphenol F and bisphenol S with adiponectin/leptin ratio among children from the Environment and Development of Children cohort*. Environment International, 185, 108564[7][8].

Liu, B., Lehmler, H. J., Sun, Y., … & Wallace, R. B. (2017). *Bisphenol A substitutes and obesity in US adults: analysis of a population-based, cross-sectional study*. The Lancet Planetary Health, 1(3), e114–e122[1].

Liu, B., Lehmler, H. J., Sun, Y., … & Snetselaar, L. G. (2019). *Association of bisphenol A and its substitutes, bisphenol F and bisphenol S, with obesity in US children and adolescents*. Diabetes & Metabolism Journal, 43(1), 59–75[4].

Meng, Z., Wang, D., … & Zhu, W. (2019). *Perinatal exposure to bisphenol S (BPS) promotes obesity development by interfering with lipid and glucose metabolism in male mouse offspring*. Environmental Research, 173, 189–198[11][14].

Philips, E. M., Jaddoe, V. W., … & Tiemeier, H. (2018). *Bisphenol and phthalate concentrations and its determinants among pregnant women in a population-based cohort in the Netherlands, 2004–5*. Environmental Research, 161, 562–572[2].

Sharma, P., & Mandal, M. B. (2021). *A comparative study of effects of 28-day exposure of bisphenol A and bisphenol S on body weight changes, organ histology, and relative organ weight in rats*. International Journal of Applied & Basic Medical Research, 11(4), 214–220[19].

Wen, X., Xiao, Y., … & Li, T. (2023). *Bisphenol S induces brown adipose tissue whitening and aggravates diet-induced obesity in an estrogen-dependent manner*. Cell Reports, 42(12), 113504[16][17].

Butylparaben

HUMAN RESULTS

Several epidemiological studies have examined whether butylparaben exposure relates to obesity or adiposity outcomes. Prospective mother–child cohort studies indicate that prenatal butylparaben exposure is associated with higher body fat or increased risk of childhood . In contrast, some cross-sectional studies of the general population have reported **inverse** associations between urinary parabens (including butylparaben) and adiposity measures. Key human study findings are summarized in **Table 1**.

Table 1. Human epidemiological studies on butylparaben exposure and adiposity outcomes.

Reference	Design	N	Exposure Metric	Adiposity Effect	p / 95% CI	Notes
Leppert *et al.* (2020)	Prospective cohort (maternal-child) – Germany (LINA)	223 mother–child pairs	Maternal urinary BuP (3rd vs 1st tertile)	↑ **risk of child overweight** by age 8 (OR=2.17 vs low exposure); effect stronger in girls	OR 2.17 (1.06–4.47) in children; girls had larger BMI increase	Maternal use of paraben-containing cosmetics linked to higher BuP levels. No effect on birth weight, but higher BMI trajectory in exposed (especially girls).
Højsager *et al.* (2021)	Prospective cohort (Odense Child Cohort) – Denmark	201 mother–son pairs (boys)	Maternal urine BuP detection (vs none)	↑ **childhood body fat% (boys)** – at 7 years old, sons had **17% higher total fat** and **23% higher abdominal fat** if mothers had BuP exposure	17% increase (3.0–32% CI); 23% increase (5.1–43% CI); $p<0.05$	No significant fat effect in daughters or with other parabens. Suggests prenatal BuP may predispose to higher adiposity in males.
Wen *et al.* (2020)	Prospective pregnancy cohort – China	518 pregnancies	Urinary parabens (∑5 parabens molar sum) by trimester	↑ **Gestational weight gain (GWG)** – Higher paraben exposure, especially in 1st trimester, linked to **greater GWG rate**	$p<0.05$ (higher GWG in high exposure vs low)	Association strongest in overweight/obese mothers. Paraben mixture in early pregnancy increased weekly weight gain, a predictor of later obesity risk.
Quirós-Alcalá *et al.* (2018)	Cross-sectional (NHANES 2007–14) – USA	4,730 adults; 1,324 children	Urinary BuP (spot biomonitoring)	↓ **Obesity prevalence** – Higher BuP levels associated with **lower odds of obesity** in adults (no increase in children)	Adults: adjusted OR for obesity per 10× BuP *<*1 (inverse) (NS for children)	Inverse dose–response trends observed for methyl, propyl, *and* butylparaben in adults. Authors caution this could reflect reverse causation or sequestration.

ANIMAL RESULTS

Multiple controlled animal studies have investigated adiposity-related endpoints following butylparaben exposure. Experiments in mice and rats generally support that butylparaben can induce weight gain or metabolic changes under certain conditions. For example, prenatal or perinatal butylparaben exposure led to increased body weight in offspring in several studies. Table 2 highlights key findings from animal studies.

Table 2. Animal studies on butylparaben exposure and adiposity/obesity-related outcomes.

Reference	Species	Design (Exposure)	Dose(s)	Adiposity Effect	p / 95% CI	Notes
Leppert *et al.* (2020)	Mouse (C57BL/6)	Perinatal exposure (dams during gestation & lactation) – s.c. injections	1.75 µg twice/week (≈ low dose)	↑ **Offspring weight (females)** – Female pups were ~20–45% heavier than controls by adulthood (no effect in male pups)	+3 g body weight vs controls (SE 0.5 g, *p*≈5×10^–9)	Maternal BuP exposure increased female offspring's food intake and weight gain. Mechanism linked to epigenetic POMC gene silencing and elevated appetite in females.
Maske *et al.* (2020)	Rat (Wistar)	Perinatal exposure (GD6–PND21) – s.c. dosing (F0 dams)	10, 100, 1000 mg/kg/day	↑ **Offspring weight (males)** – **Low dose (10 mg/kg)** led to **higher body weights** in F1 male pups (significantly above controls from birth to PND75). No weight gain at highest dose (1000 mg/kg)	*p*<0.05 at 10 mg/kg (male pup weight vs control); n.s. at 1000 mg/kg	Low-dose BuP had developmental obesogenic effect in male progeny. High-dose may have caused toxicity blunting growth (no weight difference at 1000 mg/kg). Both male and female offspring showed reproductive endocrine disruption in this study.

Reference	Species	Design (Exposure)	Dose(s)	Adiposity Effect	p / 95% CI	Notes
Du *et al.* (2024)	Mouse (strain NR)	Adult exposure (10-week oral feeding)	0, 0.5, 5, 50 mg/kg/day (est.)*	**Metabolic dysfunction** – BuP-exposed mice developed **glucose intolerance** and **hyperlipidemia** (elevated blood lipids). Also showed increased hepatic lipogenesis and insulin resistance via FXR signaling disruption.	$p<0.05$ (impaired glucose tolerance vs control)	Demonstrates butylparaben's potential to induce obesity-related metabolic disorders. No marked change in body weight reported in abstract (focus on metabolic endpoints). Gut microbiota dysbiosis observed, contributing to metabolic effects.

CONCLUSION

Human Evidence: The epidemiological evidence linking butylparaben to adiposity is **inconsistent**. Some longitudinal cohorts provide **limited evidence** of a positive association between prenatal butylparaben exposure and increased childhood adiposity. However, cross-sectional studies have found null or even inverse correlations in adults. Overall, human data suggest at most a **limited** obesogenic effect of butylparaben, with sex-specific differences noted (possible greater impact in female offspring).

Animal Evidence: In experimental animals, there is **moderate evidence** that butylparaben can act as an obesogen under certain exposure scenarios. Maternal exposure in rodents has been shown to increase offspring body weight and fat accumulation, and adult exposures induce metabolic disturbances relevant to obesity (e.g. insulin resistance, dyslipidemia). While high doses can cause toxicity without weight gain, the overall animal findings support a potential causal link between butylparaben exposure and increased adiposity or related metabolic endpoints.

References (APA style)

11. Leppert, B., Strunz, S., Seiwert, B., et al. (2020). *Maternal paraben exposure triggers childhood overweight development*. **Nature Communications, 11(1)**, 561. DOI: 10.1038/s41467-019-14202-1.

12. Højsager, F. D., Kyhl, H. B., Frederiksen, H., et al. (2021). *Prenatal exposure to butyl paraben is associated with fat percentage in 7-year-old boys*. **Journal of Clinical Endocrinology & Metabolism, 106(7)**, e2633–e2638. DOI: 10.1210/clinem/dgab167.

13. Wen, Q., Zhou, Y., Wang, Y., et al. (2020). *Association between urinary paraben concentrations and gestational weight gain during pregnancy*. **Journal of Exposure Science & Environmental Epidemiology, 30(5)**, 845–855. DOI: 10.1038/s41370-020-0205-7.

14. Quirós-Alcalá, L., Buckley, J. P., & Boyle, M. (2018). *Parabens and measures of adiposity among adults and children from the U.S. general population: NHANES 2007–2014*. **International Journal of Hygiene and Environmental Health, 221(4)**, 652–660. DOI: 10.1016/j.ijheh.2018.03.006.

15. Kim, J., & Chevrier, J. (2020). *Exposure to parabens and prevalence of obesity and metabolic syndrome: An analysis of the Canadian Health Measures Survey*. **Science of the Total Environment, 713**, 135116. DOI: 10.1016/j.scitotenv.2019.135116.

16. Du, H., Cui, L., Zhao, X., et al. (2024). *Butylparaben induces glycolipid metabolic disorders in mice via disruption of gut microbiota and FXR signaling*. **Journal of Hazardous Materials, 474**, 134821. DOI: 10.1016/j.jhazmat.2024.134821.

17. Maske, P., Dighe, V., Mote, C., & Vanage, G. (2020). *n-Butylparaben exposure through gestation and lactation impairs spermatogenesis and steroidogenesis causing reduced fertility in the F1 generation male rats*. **Environmental Pollution, 256**, 112957. DOI: 10.1016/j.envpol.2019.112957.

18. Garcia, T., Schreiber, E., Kumar, V., et al. (2017). *Effects on the reproductive system of young male rats of subcutaneous exposure to n-butylparaben*. **Food and Chemical Toxicology, 106**, 47–57. DOI: 10.1016/j.fct.2017.05.031.

Dibutyltin (DBT)

Human Results

Table 1. Human observational studies on DBT exposure and adiposity outcomes.

Reference (Year)	Design (Population)	N	Exposure metric	Adiposity effect	p/CI	Notes
Rantakokko *et al.* (2014)	Prospective birth cohort (Finland)	110 boys	Placental DBT (ng/g)	No significant association with infant weight gain or ponderal index up to 18 months	$p > 0.05$	DBT was detected in ~65% of placentas; TBT (but not DBT) showed a positive association with early postnatal weight gain.

Animal Results

Table 2. Laboratory animal studies (direct DBT exposure) evaluating adiposity endpoints.

Reference (Year)	Species	Design (Exposure Window)	Dose(s)	Adiposity effect	p/CI	Notes
Chamorro-García *et al.* (2018)	Mouse (C57BL/6J)	Perinatal exposure (gestation & lactation via drinking water); offspring evaluated in adulthood (with high-fat diet challenge)	0.5, 5, 50 µg/kg/day (5, 50, 500 nM) DBT; plus vehicle or TBT control	↑ Body fat in male offspring at highest dose (50 µg/kg/day); no adiposity increase in females. Male DBT offspring also showed elevated plasma leptin and impaired glucose tolerance	$p < 0.05$	Increased fat mass in DBT-exposed males became significant by 11 weeks of age under high-fat diet; no effect under standard diet. No significant fat or weight gain in females.

Conclusion

Human evidence: There is no epidemiological or clinical evidence that DBT exposure increases adiposity in humans (one small cohort study found no association). **Animal evidence:** There is limited evidence from animal studies (one mouse study) that perinatal DBT exposure can increase adiposity and metabolic disorder markers in offspring, with effects observed in male mice. Overall, the strength of evidence that DBT acts as an obesogen is **none to limited in humans** and **limited in animals** at present.

References:

19. Rantakokko, P., Main, K. M., Wohlfart-Veje, C., Kiviranta, H., Airaksinen, R., Vartiainen, T., … Virtanen, H. E. (2014). *Association of placenta organotin concentrations with growth and ponderal index in 110 newborn boys from Finland during the first 18 months of life: a cohort study*. **Environmental Health, 13**(45), 1–9[1].

20. Chamorro-García, R., Shoucri, B. M., Willner, S., Käch, H., Janesick, A., & Blumberg, B. (2018). *Effects of perinatal exposure to dibutyltin chloride on fat and glucose metabolism in mice, and molecular mechanisms, in vitro*. **Environmental Health Perspectives, 126**(5), 057006[2][3].

Dichlorodiphenyldichloroethylene

Human Results

Table 1: Epidemiological studies on p,p′-DDE exposure and adiposity outcomes in humans

Reference	Design	N	Exposure (timing)	Adiposity outcome effect	p/CI	Notes
Agay-Shay et al. 2015	Birth cohort (Spain)	470	Maternal serum DDE (pregnancy)	Higher BMI z-score and overweight risk at 7 y	RR (organochlorines tertile 3 vs 1) = 2.59 (95% CI 1.19–5.63)	Positive association for prenatal DDE with childhood overweight (multi-pollutant analysis).
Cupul-Uicab et al. 2010	Birth cohort (Mexico)	788	Maternal serum DDE (at birth)	No difference in child BMI (SDS) up to 2 y	High vs low exposure: ΔBMI SDS ≈ 0 (NS)	Boys followed to 18 months; prenatal DDE did not affect infant growth (height or BMI).
Delvaux et al. 2014	Birth cohort (Belgium)	114	Cord plasma DDE (birth)	↑ Abdominal fat in girls at 7–9 y	Waist circ. ↑ (girls, $p<0.05$)	In girls, higher prenatal DDE associated with greater waist circumference and waist/height ratio; no effect in boys.
Høyer et al. 2014	Multi-cohort (GL, PL, UA)	1109	Maternal serum DDE (pregnancy)	No clear association with BMI at 5–9 y	ΔBMI z (top vs bottom tertile) = –0.10 (95% CI –0.30, 0.10)	Large study in Greenland, Poland, Ukraine: prenatal (and lactational) DDE showed no significant effect on childhood BMI.
Warner et al. 2014	Birth cohort (USA, CHAMACOS)	261	Maternal serum DDE (pregnancy)	↑ Obesity odds in boys at 9 y	OR (10-fold DDE in utero) = 1.97 (0.94, 4.13) in boys (NS trend)	Prenatal DDE exposure associated with higher BMI, obesity measures in 9-year-old boys (not girls).
Tang-Péronard et al. 2015	Child cohort (Denmark)	509	Serum DDE at 8–10 y (childhood)	No weight gain difference up to 12 years later	–	Low-exposure population; childhood DDE levels did not predict overweight status in adolescence/young adulthood.
Vafeiadi et al. 2015	Birth cohort (Greece, Rhea)	689	Maternal serum DDE (1st trimester)	↑ BMI z-score and obesity at 4 y	β (BMI z) = 0.27 (95% CI 0.04, 0.50); RR_abdominal obesity = 3.76 (1.70, 8.30)	Prenatal DDE associated with higher child adiposity (BMI z) and 3.8-fold higher risk of abdominal obesity at age 4.

Notes: Prenatal exposure to p,p′-DDE showed positive associations with increased childhood adiposity in several prospective studies. Meta-analysis of these cohorts found a small but significant increase in BMI z-score (≈0.13 per log DDE) or in girls), while others found no significant association. Overall, the human evidence moderately supports a link between prenatal p,p′-DDE exposure and higher adiposity in offspring.

Animal Results

Table 2: In vivo studies of p,p′-DDE exposure and adiposity outcomes in animals

Reference	Species	Design (exposure)	Dose	Adiposity effect	p/CI	Notes
Howell et al. 2014	Mouse (C57BL/6H, M)	Adult acute exposure (gavage 5 d)	2.0 mg/kg d (oral)	No weight gain; *metabolic* dysfunction	–	Caused fasting hyperglycemia without insulin resistance. No significant change in body weight reported (short-term study).
Howell et al. 2015	Mouse (C57BL/6H, M)	Chronic + High-Fat Diet (13 w)	2.0 mg/kg weekly	*Early:* ↑ fasting glucose; *Late:* no obesity	–	DDE exacerbated HFD-induced hyperglycemia at 4–8 weeks, but by 13 weeks DDE-treated mice showed normalized glucose and reduced fatty liver. No persistent increase in body weight relative to HFD controls.
Pestana et al. 2017	Mouse (C57BL/6J, M)	Chronic 12 wks ± High-Fat Diet	0.5 mg/kg d (oral)	**Impaired adipose function**; + obesity traits with HFD	↑ adipocyte size (HF+DDE vs HF)	DDE alone did not increase weight on standard diet, but it **mimicked** HFD effects on adipose tissue and **aggravated fat storage** under HFD. Induced adipose inflammation and metabolic dysregulation ("dysmetabolic obesity") without significantly altering total body weight.
Migliaccio et al. 2023	Rat (Wistar, M)	Chronic 12 wks ± High-Fat Diet	1.0 mg/kg d (oral)	*BAT whitening:* ↑ lipid storage in BAT	$p<0.0001$ (diet × DDE)	DDE exposure, especially with HFD, led to **larger lipid droplets in brown adipose** (reduced thermogenesis), indicating promotion of an obesity-prone

Reference	Species	Design (exposure)	Dose	Adiposity effect	p/CI	Notes
						phenotype ("brown fat whitening").

Notes: In vivo experiments show that p,p′-DDE can disrupt metabolic homeostasis and adipose tissue function even without large changes in body weight. Short-term DDE exposure in adult mice caused persistent fasting hyperglycemia. In longer studies, chronic low-dose DDE exposure interacted with diet: by itself DDE did not markedly increase adiposity, but under high-fat diet it exacerbated adipose tissue enlargement and inflammation. DDE-treated rodents on high-fat diets showed evidence of **greater fat accumulation in adipocytes** and impaired thermogenic response, despite similar weight gain to controls. These findings suggest DDE acts as a metabolic disruptor ("obesogen") by **promoting adipocyte lipid storage and dysfunction** rather than by dramatically increasing overall body mass.

Conclusion

In summary, there is **moderate evidence** in humans that developmental exposure to p,p′-DDE is associated with modest increases in childhood adiposity (higher BMI or fat measures), with some inconsistencies across studies. In animal models, the evidence is also **moderate**: p,p′-DDE alone induces metabolic disturbances and enhances fat accumulation under obesogenic diet conditions, supporting its role as an obesogen. Taken together, the findings from epidemiological cohorts and direct animal exposures **presume p,p′-DDE to be obesogenic** (favoring increased adiposity), albeit with relatively small effect sizes and dependent on contextual factors (e.g. sex, diet). The overall strength of evidence is therefore **moderate** for both humans and animals that p,p′-DDE exposure can contribute to increased adiposity or obesity-related endpoints.

References:

Agay-Shay, K., Martinez, D., Valvi, D., Garcia-Esteban, R., Basagaña, X., Robinson, O., et al. (2015). Exposure to endocrine-disrupting chemicals during pregnancy and weight at 7 years of age: a multi-pollutant approach. *Environmental Health Perspectives*, 123(10), 1030–1037.[26][2]

Cupul-Uicab, L. A., Hernández-Avila, M., Terrazas-Medina, E. A., Pennell, M. L., & Longnecker, M. P. (2010). Prenatal exposure to the major DDT metabolite 1,1-dichloro-2,2-bis(p-chlorophenyl)ethylene (DDE) and growth in boys from Mexico. *Environmental Research*, 110(6), 595–603.[4]

Delvaux, I., Van Cauwenberghe, J., Den Hond, E., Schoeters, G., Govarts, E., Nelen, V., et al. (2014). Prenatal exposure to environmental contaminants and body composition at age 7–9 years. *Environmental Research*, 132, 24–32.[5]

Høyer, B. B., Ramlau-Hansen, C. H., Henriksen, T. B., Pedersen, H. S., Góralczyk, K., Zviezdai, V., et al. (2014). Body mass index in young school-age children in relation to organochlorine compounds in early life: a prospective study. *International Journal of Obesity*, 38(7), 919–925.[6]

Howell, G. E. III, Meek, E. A., Kilic, J., Mohns, M. S., Mulligan, C., & Chambers, J. E. (2014). Exposure to p,p′-DDE induces fasting hyperglycemia without insulin resistance in male C57BL/6H mice. *Toxicology*, 320, 6–14.[15]

Howell, G. E. III, Mulligan, C., Meek, E., & Chambers, J. E. (2015). Effect of chronic p,p′-DDE exposure on high fat diet-induced alterations in glucose and lipid metabolism in male C57BL/6H mice. *Toxicology*, 328, 112–122.[17]

Migliaccio, V., Di Gregorio, I., Penna, S., Panico, G., Lombardi, A., & Lionetti, L. (2023). Adaptation of brown adipose tissue in response to chronic exposure to the environmental pollutant p,p′-DDE and/or a high-fat diet in male Wistar rats. *Nutrients*, 16(16), 2616.[19][23]

Pestana, D., Teixeira, D., Meireles, M., Marques, C., Norberto, S., Sá, C., et al. (2017). Adipose tissue dysfunction as a central mechanism leading to dysmetabolic obesity triggered by chronic exposure to p,p′-DDE. *Scientific Reports*, 7(1), 2738.[22][19]

Tang-Péronard, J. L., Jensen, T. K., Andersen, H. R., Ried-Larsen, M., Grøntved, A., Andersen, L. B., et al. (2015). Associations between exposure to persistent organic pollutants in childhood and overweight up to 12 years later in a low exposed Danish population. *Obesity Facts*, 8(4), 282–292.[9]

Vafeiadi, M., Georgiou, V., Chalkiadaki, G., Rantakokko, P., Kiviranta, H., Karachaliou, M., et al. (2015). Association of prenatal exposure to persistent organic pollutants with obesity and cardiometabolic traits in early childhood: the Rhea mother–child cohort (Crete, Greece). *Environmental Health Perspectives*, 123(10), 1015–1021.[12][11]

Warner, M., Wesselink, A., Harley, K. G., Bradman, A., Kogut, K., & Eskenazi, B. (2014). Prenatal exposure to dichlorodiphenyltrichloroethane and obesity at 9 years of age in the CHAMACOS study cohort. *American Journal of Epidemiology*, 179(11), 1312–1322.[8][7]

Dolutegravir

Human Results

Table 1: Summary of human studies on dolutegravir and adiposity outcomes.

Reference	Design	N	Exposure (comparison)	Adiposity effect	p / 95% CI	Notes
Venter *et al.* (2019)	RCT (ADVANCE trial, S. Africa)	1,053	DTG+TAF vs DTG+TDF vs EFV+TDF (48 weeks first-line ART)	48-week weight gain: **+6 kg** in DTG+TAF arm vs **+3 kg** in DTG+TDF vs **+1 kg** in EFV arm; new obesity in	p<0.001 (DTG+TAF vs EFV)	Women gained significantly more than men on DTG regimens. Weight gain not explained by

Reference	Design	N	Exposure (comparison)	Adiposity effect	p / 95% CI	Notes
				**14%**, 7%, 6% respectively. Greater gains with DTG (especially DTG+TAF) than EFV.		"return to health" alone.
Sokhela *et al.* (2024)	RCT (ADVANCE trial 192-week follow-up)	622 (at 192 w)	DTG+TAF vs DTG+TDF vs EFV+TDF (4 years)	192-week weight gain: **+8.9 kg** (DTG+TAF) vs **+5.9 kg** (DTG+TDF) vs **+3.2 kg** (EFV). New obesity in **29%** (DTG+TAF) vs 21% (DTG+TDF) vs 15% (EFV). Most excess gain occurred in first 96 weeks.	$p<0.001$ (higher obesity incidence on DTG+TAF)	Weight gain was greatest in women, with DTG+TAF causing 43% of women to become obese. No differences in hypertension by 192 w.
Sax *et al.* (2020)	Pooled analysis of 8 RCTs	5,680	INSTI vs NNRTI vs PI (ART initiation trials)	Integrase inhibitor (INSTI) regimens caused **greater weight gain** than NNRTI or PI-based regimens. **Dolutegravir** and bictegravir led to more weight gain than older INSTIs (e.g. raltegravir). TAF-based NRTI backbones also added more weight than TDF or other NRTIs.	–	Weight gain was multifactorial: more pronounced with low baseline CD4, high viral load, female sex, and Black race. Suggests a class effect of DTG on weight gain beyond "return to health."
Bansi-Matharu *et al.* (2021)	Prospective cohort (RESPOND)	14,703	Various ART exposures (cohort observation)	**Dolutegravir exposure** was independently associated with significant BMI increase: OR≈**1.27** for >7% BMI gain vs reference. **DTG+TAF combined** had higher odds	OR 1.27, 95%CI 1.17–1.38 (DTG vs ref); OR 1.79, 95%CI 1.52–2.11 (DTG+TAF vs ref).	Weight gain risk was greater in those with low pre-ART BMI and in Black patients. Both **dolutegravir** and **tenofovir alafenamide** had **additive effects**

Reference	Design	N	Exposure (comparison)	Adiposity effect	p / 95% CI	Notes
				(OR≈**1.79**) of substantial BMI gain. Raltegravir and TAF similarly associated with weight gain.		on increasing adiposity.

Animal Results

Table 2: Summary of animal studies on dolutegravir exposure and adiposity outcomes.

Reference	Species	Design	Dose(s)	Adiposity effect	p / CI	Notes
Acharya *et al.* (2025) (Sci. Reports)	Mouse (C57BL/6J, ♀)	8-week DTG treatment vs control (daily oral; 1× or 5× human-equivalent dose)	~50 mg/kg/day (1×); 5× higher in high-dose group	**No significant weight gain** or food intake difference in DTG-treated mice vs controls over 8 weeks. All groups gained weight at similar rates; DTG did not increase adiposity.	n.s. (no group differences)	Mild transient hyperglycemia observed in DTG groups (elevated glucose weeks 1–4), along with lower leptin levels in white fat (non-significant). No changes in adipose gene markers like adiponectin or Ucp1.
Kress *et al.* (2024) (Vasc. Pharmacol)	Mouse (WT and Tg26 HIV-transgenic, ♂)	12-week DTG exposure via diet vs vehicle (with and without HIV viral protein expression)	7 mg/kg/day in chow	**No increase in body weight or fat mass** attributable to DTG in either wild-type or HIV-Tg mice. DTG-treated mice showed a slight rise in brown fat mass and food intake but **no exacerbation of weight gain** vs controls.	n.s. (DTG vs control)	DTG alone caused some metabolic changes (↑BAT thermogenesis, ↑food intake) but **failed to induce obesity** in healthy or HIV-protein mice. Authors suggest **dolutegravir by itself is insufficient** to drive weight gain; other ART drugs or illness ("return

Reference	Species	Design	Dose(s)	Adiposity effect	p / CI	Notes
						to health") likely needed.
Ngono Ayissi *et al.* (2022) (*Cells*)	Macaque (Cynomolgus)	SIV-infected macaques on DTG-based ART vs untreated controls (≈2 years therapy)	~20 mg/kg DTG daily (with TDF+FTC)	**Adipose tissue remodeling** observed with chronic DTG: treated macaques had **adipocyte hypertrophy** and elevated fat **fibrosis**, along with **lower "beige" fat gene expression** (thermogenic markers) vs controls. Integrase inhibitors (DTG, BIC) inhibited the browning of white fat in vitro and induced an **insulin-resistant, pro-fibrotic adipocyte phenotype**.	–	Despite adipose changes, **no gross obesity** was reported in DTG-treated macaques (body weights similar to controls). Findings imply that dolutegravir may promote **qualitative fat changes** (reduced energy expenditure capacity of fat) that could predispose to adiposity.

Conclusion

In **humans**, **strong evidence** indicates that dolutegravir (especially in combination with tenofovir alafenamide) causes increased weight gain and higher rates of obesity compared to alternative therapies. Multiple randomized trials and large cohorts consistently link dolutegravir-based ART with significant gains in body weight and fat (particularly among women and those with other risk factors). In **animals**, evidence is **limited**. Short-term mouse studies have not shown dolutegravir alone to increase body weight or fat mass, although mechanistic findings in treated SIV-macaques reveal adipose tissue changes (hypertrophy, fibrosis, impaired "beige" fat thermogenesis) that could contribute to obesity. Overall, dolutegravir's propensity to induce adiposity is well-supported in clinical settings, whereas

animal models so far show **no clear obesity outcome** without additional factors, suggesting the weight gain may result from a combination of drug effects and host/contextual factors.

References:

21. Venter, W. D. F., et al. (2019). Dolutegravir plus Two Different Prodrugs of Tenofovir to Treat HIV. *New England Journal of Medicine*, **381**(9), 803-815[1][33].

22. Sokhela, S., et al. (2024). Final 192-Week Efficacy and Safety Results of the ADVANCE Trial, Comparing 3 First-line Antiretroviral Regimens. *Open Forum Infectious Diseases*, **11**(3): ofae007[5][6].

23. Sax, P. E., et al. (2020). Weight gain following initiation of antiretroviral therapy: risk factors in randomized comparative trials. *Clinical Infectious Diseases*, **71**(6), 1379-1389[10].

24. Bansi-Matharu, L., et al. (2021). Contemporary antiretrovirals and body-mass index: a prospective study of the RESPOND cohort consortium. *Lancet HIV*, **8**(11), e711–e722[13][14].

25. Acharya, P., et al. (2025). Effect of dolutegravir-based antiretroviral therapy on glycemic control in female mice. *Scientific Reports*, **15**, Article 22130[16][19].

26. Kress, T. C., et al. (2024). 12-week dolutegravir treatment marginally reduces energy expenditure but does not increase body weight or alter vascular function in a murine model of HIV infection. *Vascular Pharmacology*, **155**, 107288[21][22].

27. Ngono Ayissi, K., et al. (2022). Inhibition of adipose tissue beiging by HIV integrase inhibitors, dolutegravir and bictegravir, is associated with adipocyte hypertrophy, hypoxia, elevated fibrosis, and insulin resistance in simian adipose tissue and human adipocytes. *Cells*, **11**(11), 1841[29].

Fenthion

HUMAN RESULTS

A large cross-sectional study of Thai farmers (Noppakun & Juntarawijit, 2022) reported that long-term use of organophosphate insecticides (including multiple agents in the same class as fenthion) was significantly associated with higher obesity prevalence. In contrast, a cross-sectional analysis of U.S. adults (Cai *et al.*, 2024) found that higher urinary organophosphate metabolite levels (which reflect exposure to fenthion and related pesticides) were associated with *lower* body mass index (BMI) and waist circumference, and reduced odds of obesity. No human clinical trials or cohort studies specifically isolating fenthion exposure and adiposity were identified.

Table 1: Human epidemiological studies on fenthion (or organophosphate) exposure and adiposity outcomes

Reference	Design	N	Exposure metric	Adiposity effect	p/CI	Notes
Noppakun & Juntarawijit (2022)	Cross-sectional (farmers, Thailand)	20,295	Self-reported long-term use of organophosphate insecticides (including fenthion class)	Higher obesity prevalence in pesticide users (adjusted OR for insecticide use ~2.10; several	$p<0.05$ (for 22 of 35 pesticides)	Multiple OP insecticides (grouped) significantly associated with increased odds

Reference	Design	N	Exposure metric	Adiposity effect	p/CI	Notes
				individual OP insecticides OR range 1.8–8.3)		of obesity; specific fenthion contribution not isolated.
Cai *et al.* (2024)	Cross-sectional (NHANES adult cohort, USA)	9,505	Urinary OPP metabolites (DMP, DEP, DMTP, DETP) as proxy for organophosphate pesticide exposure	**Inverse** association: higher metabolite levels correlated with lower BMI and waist circumference; lower obesity odds (e.g. DETP OR=0.85, 95% CI 0.80–0.90)	p<0.01 for trend (all four metabolites)	Higher fenthion-related metabolite levels were linked to **decreased** obesity prevalence in this general population sample.

ANIMAL RESULTS

In laboratory animals, several studies have observed body-weight increases after fenthion exposure at sub-toxic doses. In chronic dietary studies, fenthion-treated rodents showed significantly higher body weights than controls at moderate dose levels. For example, in a 2-year mouse study, the high fenthion dose (25 ppm in feed, ~9–11 mg/kg/day) led to >10% greater final body weight compared to controls. Similarly, in rats, a mid-range dose (5 ppm, ~2 mg/kg/day) caused a small (~5%) but significant weight gain, and 25 ppm (~9–10 mg/kg/day) caused ~10% weight increase. However, at higher, overtly toxic doses, fenthion exposure inhibited weight gain or caused weight loss (e.g. in rats at 100 ppm feed, body weights were significantly lower than controls due to cholinergic toxicity). No animal studies specifically measuring fat mass or adiposity (e.g. fat pad weights) were found in the eligible literature.

Table 2: Animal studies of fenthion exposure and adiposity-related outcomes

Reference	Species	Design	Dose(s) & duration	Adiposity effect	p/CI	Notes
Leser & Suberg (1990, 1992) (via WHO/IPCS 1996)	**Mouse** ($B6C3F_1$)	Chronic feeding study (oncogenicity assay) – 2 years	0, 0.1, 1, 5, 25 ppm in diet (♂ ~0–9.4 mg/kg/day; ♀ ~0–10.6 mg/kg/day)	Increased terminal body weight at high dose: ~+10% vs controls at 25 ppm (significant weight gain)	p<0.05 (trend)	High-dose mice had elevated body weight accompanied by enlarged liver and kidneys. No obesity noted at lower doses; no increase in tumors.

Reference	Species	Design	Dose(s) & duration	Adiposity effect	p/CI	Notes
Van Goethem & Leser (1993) (via WHO/IPCS 1996)	**Rat** (Wistar)	Chronic feeding study – 2 years	0, 0.1, 1, 5, 25 ppm in diet; separate high-dose group 100 ppm (♂ ~0–5.2 mg/kg; ♀ ~0–7.3 mg/kg/day)	**Biphasic effect**: At 5 ppm, slight ↑ body weight (~4–5%); at 25 ppm, ↑ body weight (~10%) in both sexes. Highest dose (100 ppm) caused ↓ body weight (−8% by mid-study in males) due to toxicity.	$p<0.05$ (5 & 25 ppm vs control); $p<0.01$ (100 ppm vs control)	Weight gain considered treatment-related at 5–25 ppm (no overt toxicity). Cholinergic signs (e.g. tremors, anorexia) at 100 ppm accompanied reduced weight gain. No significant fat pad analyses reported.

CONCLUSION

Human evidence: *Inconsistent.* No direct clinical studies on fenthion and obesity were found. Epidemiological data are limited and mixed: one large cross-sectional study suggests organophosphate pesticide use (possibly including fenthion) correlates with higher obesity rates, while an analysis of U.S. biomonitoring data found higher organophosphate exposure associated with *lower* obesity prevalence. Overall, there is limited and inconsistent evidence in humans regarding fenthion and adiposity.

Animal evidence: *Limited-to-moderate.* Repeated fenthion exposure in rodents can lead to increased body weight at sub-toxic doses, suggesting a potential obesogenic effect. However, these findings come primarily from toxicity studies (not designed to assess obesity), and severe cholinergic toxicity at high doses reverses this trend (causing weight loss). No dedicated animal studies of fenthion on adipose tissue or metabolic endpoints were identified. Hence, the animal evidence for fenthion increasing adiposity is suggestive but remains limited in scope.

References:

28. **APVMA (2014).** *Preliminary Review Findings: Fenthion – Volume 2 (Technical Reports).* Australian Pesticides and Veterinary Medicines Authority, Canberra. (Includes chronic toxicity studies in rodents)[5][6].

29. **Cai, Y.**, Wang, S., Xu, H., *et al.* (2024). Association between organophosphorus pesticides and obesity among American adults. *Environmental Health*, **23**(1): 31. DOI: 10.1186/s12940-024-01104-z [2][3].

30. **Noppakun, K.**, & Juntarawijit, C. (2022). Association between pesticide exposure and obesity: A cross-sectional study of 20,295 farmers in Thailand. *F1000Research*, **10**: 445. DOI: 10.12688/f1000research.51253.3 [1].

Glibenclamide

Human Results

Studies in diabetic patients indicate that glibenclamide (glyburide) therapy is associated with modest weight gain relative to other treatments or placebo. For example, in the UKPDS trial, patients on glibenclamide gained about 1.7 kg more over 10 years than those on diet alone (p<0.001). Similarly, a 5-year trial (ADOPT) reported a net weight increase (~1.6 kg) with glyburide therapy, whereas an alternative drug (metformin) led to weight loss. A retrospective cohort study found that glibenclamide users lost less weight over 1 year than those on glimepiride (−0.58 kg vs −2.04 kg, p<0.001) despite similar glycemic control. **Table 1** summarizes human evidence from clinical and observational studies.

Table 1: Human studies on glibenclamide exposure and adiposity outcomes.

Reference (APA)	Design	N (subjects)	Exposure metric	Adiposity effect	p-value / 95% CI	Notes
UKPDS Group (1998)	Randomized trial (10-year intensive vs conventional therapy)	3,867	Sulfonylurea (glibenclamide) vs diet control	+1.7 kg greater weight gain vs diet control	p<0.001	Weight gain with glibenclamide (~1.7 kg) over 10 years, vs minimal gain on diet
Kahn et al. (2006)	Randomized trial (ADOPT, 5-year follow-up)	4,360	Glyburide monotherapy (vs rosiglitazone or metformin)	~+1.6 kg weight increase from baseline (5-year)	–	Glyburide caused modest weight gain (rosiglitazone arm had +4.8 kg; metformin arm had weight loss)
Martin et al. (2003)	Retrolective cohort (12-month therapy in T2DM outpatients)	520	Glibenclamide vs glimepiride (initial therapy)	–0.58 kg vs –2.04 kg weight change (glib vs glime)	p<0.001	Glibenclamide group had significantly less weight reduction than glimepiride group (i.e. more weight retention)

Animal Results

In animal experiments, glibenclamide exposure has also been linked to increased adiposity endpoints. In obese diabetic rats (OLETF strain), 12-week glibenclamide treatment led to adipocyte hypertrophy in visceral fat – the mean fat-cell area in retroperitoneal fat increased by ~28% compared to untreated controls (p<0.05). Glibenclamide-treated rats showed fewer small adipocytes and a higher proportion of large adipocytes than controls. In another study using streptozotocin (STZ)-diabetic rats, daily glibenclamide (3 mg/kg) for 3 weeks significantly increased body weight compared to untreated diabetic rats (p<0.001), concurrent with higher insulin levels. **Table 2** summarizes the animal evidence.

Table 2: Animal studies on glibenclamide exposure and adiposity outcomes.

Reference (APA)	Species (model)	Design	Dose(s)	Adiposity effect	p-value / 95% CI	Notes
Mori et al. (2004)	OLETF rats (obese T2D model)	12-week drug treatment vs control (randomized)	Glibenclamide (therapeutic dose) in feed	Larger adipocytes in visceral fat (13,764 µm^2 vs ~10,800 µm^2 in controls)	p<0.05	Increased fat-cell size and elevated adipose TNF-α expression with glibenclamide (greater than with glimepiride)
Boutou et al. (2024)	Wistar rats (STZ-diabetic)	3-week treatment (diabetic vs treated groups)	Glibenclamide 3 mg/kg/day (oral)	Body weight increased vs untreated diabetics	p<0.001	Glibenclamide improved glycemia but prevented weight loss seen in untreated diabetic rats (weight gain ~30% vs baseline)

Conclusion

Humans: Strong evidence indicates that glibenclamide use can increase adiposity (weight gain) in diabetic patients, based on multiple clinical trials and cohort studies. **Animals:** Moderate evidence (from rodent models of diabetes) supports an association between glibenclamide exposure and increased adiposity (enlarged fat cells or weight gain).

References:

- UK Prospective Diabetes Study (UKPDS) Group. (1998). *Intensive blood-glucose control with sulphonylureas or insulin compared with conventional treatment and risk of complications in type 2 diabetes (UKPDS 33)*. **The Lancet, 352**(9131), 837–853.[1]
- Kahn, S. E., Haffner, S. M., Heise, M. A., Herman, W. H., Holman, R. R., Jones, N. P., … Viberti, G. (2006). *Glycemic durability of rosiglitazone, metformin, or glyburide monotherapy*. **New England Journal of Medicine, 355**(23), 2427–2443.[2][3]
- Martin, S., Kolb, H., Beuth, J., van Leendert, R., Schneider, B., & Scherbaum, W. A. (2003). *Change in patients' body weight after 12 months of treatment with glimepiride or glibenclamide in Type 2 diabetes: a multicentre retrolective cohort study*. **Diabetologia, 46**(11), 1611–1617.[4]
- Mori, Y., Komiya, H., Kurokawa, N., & Tajima, N. (2004). *Comparison of the effects of glimepiride and glibenclamide on adipose tissue TNF-α mRNA expression and cellularity*. **Diabetes, Obesity and Metabolism, 6**(1), 28–34.[5][6]
- Boutou Masky, H., Adjia, H., Miaffo, D., Oumarou, B. F. A., Foyet, H. S., Maguirgue, K., … Ntchapda, F. (2024). *Antidiabetic activity of the aqueous extract of Erigeron floribundus leaves in streptozotocin-induced type 1 diabetes model in Wistar rats*. **Metabolism Open, 22**, 100288.[7][8]

Megestrol Acetate (CAS 595-33-5)

Human Results

Table 1: Summary of human studies evaluating megestrol acetate exposure and adiposity/obesity endpoints.

Reference	Design	N	Exposure Metric	Adiposity Effect	p / 95% CI	Notes
Loprinzi *et al.*, 1990 (JNCI)	RCT (cancer cachexia, MA vs placebo)	133	800 mg/day oral, 8–12 weeks	16% of MA group gained >6.8 kg vs 2% of placebo (weight gain >15 lb)	$p = 0.003$	Advanced cancer patients with anorexia; MA significantly stimulated appetite and weight gain (primarily non-fluid weight).
Von Roenn *et al.*, 1994 (Ann Intern Med)	RCT (AIDS cachexia, dose-ranging)	270	100/400/800 mg/day, 12 weeks	At 800 mg: mean +3.54 kg vs –0.73 kg in placebo; lean mass +1.14 kg vs –0.77 kg (placebo)	$p < 0.001$ (weight & LBM)	AIDS patients with weight loss; MA (800 mg) induced significant weight gain (mostly fat mass) and modest lean increase; improved appetite.
Eubanks *et al.*, 2002 (J Pediatr)	RCT (cystic fibrosis, malnourished)	17	~7.5 mg/kg/day (mean 800 mg) for 3 months	Weight-for-age Z-score increased (reached 100% ideal weight); weight gain comprised both fat and lean mass	$p < 0.04$ (vs placebo)	CF children with pancreatic insufficiency; MA group gained weight (fat and fat-free mass) and improved lung function. Reversible adrenal suppression noted.
Lambert *et al.*, 2002 (J Clin Endocrinol Metab)	RCT (older men, all on MA ± adjuncts)	30	800 mg/day for 12 weeks (all groups)	Mean +3.8 kg weight (no between-group diff); weight gain **primarily fat** – MA alone led to muscle loss unless	$p <$ 0.0001 (weight gain); muscle CSA ↓5–6% with MA	Frail elderly men (cachectic); MA stimulated significant weight gain predominantly as adipose tissue. Co-administered

Reference	Design	N	Exposure Metric	Adiposity Effect	p / 95% CI	Notes
				resistance exercise added	alone (*p*<0.05)	testosterone did not prevent fat gain or muscle loss; exercise needed to preserve muscle.
Yeh *et al.*, 2010 (J Ren Nutr)	RCT (elderly dialysis patients, cachectic)	50	800 mg/day + resistance training, 16 weeks	Increased dry weight and BMI; **fat mass ↑ significantly more than lean mass** in MA group (bioimpedance analysis)	$p < 0.05$ (weight, BMI)	Hemodialysis patients with cachexia; MA improved appetite and nutritional status. Fat mass showed a "favorable increase" relative to lean mass (adipose gain).

Animal Results

Table 2: Summary of animal studies (direct exposure) evaluating megestrol acetate and adiposity-related endpoints.

Reference	Species	Design	Dose(s)	Adiposity Effect	p / 95% CI	Notes
Southam, 1968	Rat (female)	SC implant of MA in mature females; observed during and after treatment	**~6–10 mg** per implant (estimated release over weeks)	**Body weight ↑ during MA treatment** and remained elevated after stopping; many rats became obese post-treatment	(qualitative; weight ↑ observed)	First report of MA's anabolic effect in normal rats. Treated females showed persistent weight (fat) gain and reduced activity, with obesity noted even after treatment ended.
Beck & Tisdale, 1990	Mouse	Experimental cachexia models (TNF infusion; MAC16 tumor) with MA vs control	**25 mg/kg** and **100 mg/kg** oral, 7–10 days	MA **prevented weight loss** from cachexia; weight gain mainly due to increased **water** content, but high-dose MA also **increased carcass fat** (in tumor-bearing mice)	$p < 0.01$ (vs vehicle)	NMRI mice. MA stimulated food/water intake and preserved body weight during cachexia. Body composition: at 100 mg/kg, some fat mass increase observed despite cachexia.

Reference	Species	Design	Dose(s)	Adiposity Effect	p / 95% CI	Notes
Zhong *et al.*, 2023	Rat (male)	Cisplatin-induced cachexia model; MA and nomegestrol vs vehicle	**10 mg/kg** MA oral, 21 days	MA (10 mg/kg) **significantly attenuated weight loss** and **reversed white adipose atrophy** (adipocyte size restored) in cachectic rats	$p < 0.05$ (vs cachexia control)	Male Wistar rats. MA and an analog (NOMAc) improved body weight and adipose tissue morphology under cachexia. MA 10 mg/kg restored adipocyte volume in inguinal fat; aided fat maintenance.

Conclusion

In **humans**, there is **strong evidence** that megestrol acetate exposure causes increased adiposity. Multiple placebo-controlled trials (in cancer, HIV/AIDS, geriatric, and other populations) consistently show significant weight gain with megestrol, with the **majority of the gain as fat mass** rather than lean tissue (Loprinzi *et al.*, 1990; Von Roenn *et al.*, 1994; Lambert *et al.*, 2002). This effect is observed across diverse patient groups and doses, supporting a robust adipogenic outcome in humans.

In **animals**, the evidence is also **strong**. Controlled experiments in rodents demonstrate that megestrol reliably **increases body weight and fat deposition** or prevents fat loss under cachectic conditions (Southam, 1968; Beck & Tisdale, 1990). Both historical and recent studies confirm megestrol's hyperphagic and adiposity-promoting effects in vivo, indicating a consistent pro-obesity endpoint in animal models. Overall, megestrol acetate has a pronounced propensity to induce weight and fat gains in both human and animal exposures.

References:

- Beck, S. A., & Tisdale, M. J. (1990). *Effect of megestrol acetate on weight loss induced by tumour necrosis factor alpha and a cachexia-inducing tumour (MAC16) in NMRI mice*. British Journal of Cancer, 62(3), 420–424. DOI: 10.1038/bjc.1990.310
- Eubanks, V., Koppersmith, N., Wooldridge, N., Clancy, J. P., Lyrene, R., Arani, R. B., … Makris, C. M. (2002). *Effects of megestrol acetate on weight gain, body composition, and pulmonary function in patients with cystic fibrosis*. The Journal of Pediatrics, 140(4), 439–444. DOI: 10.1067/mpd.2002.121936
- Lambert, C. P., Sullivan, D. H., Freeling, S. A., Lindquist, D. M., & Evans, W. J. (2002). *Effects of testosterone replacement and/or resistance exercise on the composition of megestrol acetate-stimulated weight gain in elderly men: a randomized controlled trial*. Journal of Clinical Endocrinology & Metabolism, 87(5), 2100–2106. DOI: 10.1210/jcem.87.5.8505
- Loprinzi, C. L., Ellison, N. M., Schaid, D. J., Krook, J. E., Athmann, L. M., Mailliard, J. A., … Gerstner, J. B. (1990). *Controlled trial of megestrol acetate for the treatment of cancer anorexia and cachexia*. Journal of the National Cancer Institute, 82(13), 1127–1132. DOI: 10.1093/jnci/82.13.1127

- Southam, L. (1968). *Effects of megestrol acetate, a progestin, on female rats*. Proceedings of the Iowa Academy of Science, 75(1), 296–300.
- Von Roenn, J. H., Armstrong, D., Kotler, D. P., Cohn, D. L., Klimas, N. G., Tchekmedyian, N. S., … Weitzman, S. A. (1994). *Megestrol acetate in patients with AIDS-related cachexia*. Annals of Internal Medicine, 121(6), 393–399. DOI: 10.7326/0003-4819-121-6-199409150-00001
- Yeh, S. S., Marandi, M., Thode, H. C., Chen, K. M., & Schuster, M. W. (2010). *Report of a pilot, double-blind, placebo-controlled study of megestrol acetate in elderly dialysis patients with cachexia*. Journal of Renal Nutrition, 20(1), 52–62. DOI: 10.1053/j.jrn.2009.05.006
- Zhong, R., Yang, W., Li, G., Xie, S., Guo, X., Zhou, J., Ren, B., & Zhu, Y. (2023). *Nomegestrol acetate ameliorated adipose atrophy in a rat model of cisplatin-induced cachexia*. Experimental and Therapeutic Medicine, 25(1), Article 24. DOI: 10.3892/etm.2022.11723

Mono-(2-ethylhexyl) Phthalate (MEHP)

Human Results

Table 1. Summary of human observational studies on MEHP exposure and adiposity endpoints.

Reference	Design & Population	N	Exposure metric	Adiposity effect	p / CI	Notes
Buser et al., 2014	Cross-sectional (NHANES 2007–2010, U.S. adults)	~5,585 adults	Urinary phthalate metabolites (incl. MEHP)	Higher MEHP levels (as part of high-molecular-weight phthalates) associated with increased odds of obesity in adults	$p < 0.05$	Association observed in all adult men and women (cross-sectional study).
Harley et al., 2017	Prospective birth cohort (CHAMACOS, California)	345 mother–child pairs	Prenatal maternal urinary DEHP metabolites (incl. MEHP)	Higher prenatal DEHP metabolite levels associated with higher childhood BMI z-score, waist circumference, and % body fat at multiple ages. At age 12, each doubling of prenatal DEHP metabolites was associated with ~30% higher odds of overweight/obesity in the child.	OR=1.3 (95% CI: 1.0–1.6)	Association observed in both boys and girls (no significant sex interaction).
Peng et al., 2023	Longitudinal cohort (SWAN	1,369 women	Urinary phthalate	Higher baseline MEHP (and other phthalate	β ~ +0.05%	Effect strongest in

Reference	Design & Population	N	Exposure metric	Adiposity effect	p / CI	Notes
	midlife women, USA)		metabolites at baseline (incl. MEHP)	metabolites) predicted greater increases in DXA-measured body fat percentage and fat mass over ~18 years of follow-up. Magnitude was modest (on the order of 0.05–0.09 percentage point greater 5-year body fat gain per doubling of metabolite level). Weight change associations were mostly null.	BF/5yr per doubling (95% CI up to +0.16%)	women who were non-obese at baseline; associations attenuated in sensitivity analyses using a later exposure measure.

Animal Results

Table 2. Summary of animal studies of MEHP exposure and adiposity endpoints.

Reference	Species	Design (Exposure Timing)	Dose(s)	Adiposity effect	p / CI	Notes
Hao et al., 2012	Mouse (♂ offspring of C57BL/6 dams)	Perinatal (in utero exposure during gestation)	*Low dose* MEHP (exact dose not given in abstract)	Increased body weight and white fat pad weight in male offspring at postnatal day 60 compared to controls. Also elevated serum cholesterol, triacylglycerol, and glucose in exposed males.	$p < 0.05$	Sex-dependent effect: significant obesity outcome in male offspring only (no effect reported in females).

Conclusion

In humans, the evidence for MEHP exposure increasing adiposity is **inconsistent/limited**. Several observational studies (cross-sectional and prospective) report positive associations between MEHP (a DEHP metabolite) and higher body mass or fat measures, including modest but significant increases in body fat gain over time. However, results are not uniform across all populations or study designs, and potential confounding and reverse causation cannot be ruled out. In laboratory animals, **limited** evidence (from direct MEHP exposure studies) suggests that MEHP can promote adiposity: a single in vivo mouse study showed increased adipose tissue weight and body weight following perinatal MEHP exposure. Overall, current data indicate limited support for an obesogenic effect of MEHP in humans and animals, with more research needed for definitive conclusions.

References:

31. Buser, M. C., Murray, H. E., & Scinicariello, F. (2014). *Age and sex differences in childhood and adulthood obesity association with phthalates: analyses of NHANES 2007–2010*.

International Journal of Hygiene and Environmental Health, 217(6), 687–694. DOI: 10.1016/j.ijheh.2014.02.005

32. Hao, C., Cheng, X., Xia, H., & Ma, X. (2012). *The endocrine disruptor mono-(2-ethylhexyl) phthalate promotes adipocyte differentiation and induces obesity in mice*. Bioscience Reports, 32(6), 619–629. DOI: 10.1042/BSR20120042

33. Harley, K. G., Berger, K. P., Rauch, S., Kogut, K., Claus Henn, B., Calafat, A. M., Huen, K., Eskenazi, B., & Holland, N. (2017). *Association of prenatal urinary phthalate metabolite concentrations and childhood BMI and obesity*. Pediatric Research, 82(3), 405–415. DOI: 10.1038/pr.2017.112

34. Peng, M. Q., Karvonen-Gutierrez, C. A., Herman, W. H., Mukherjee, B., & Park, S. K. (2023). *Phthalate exposure is associated with more rapid body fat gain in midlife women: The SWAN Multi-Pollutant Study*. Environmental Research, 216, 114685. DOI: 10.1016/j.envres.2022.114685

Olanzapine

Human Results

Table 1: Summary of human studies on olanzapine exposure and adiposity outcomes.

Reference	Design	N	Exposure metric	Adiposity effect	p/CI	Notes
Jain *et al.*, 2006	Prospective (4-week, open-label)	80	Olanzapine 5–15 mg/day (flexible dose)	66.6% of patients gained 1–5 kg in 4 weeks	– (descriptive)	Weight gain occurred in 2/3 of patients; not dose-dependent; more pronounced in women ≥40 yrs.
Lieberman *et al.*, 2005 (CATIE trial)	RCT, 18-month, multi-arm	1,432 (330 OLA)	Olanzapine 7.5–30 mg/day (median ~6 mo on drug)	Mean +2.0 lb/month weight gain on olanzapine (≈0.9 kg/mo) – highest among antipsychotics; 30% of olanzapine patients gained >7% body weight	$p < 0.001$	Olanzapine caused significantly greater weight gain than risperidone, quetiapine, perphenazine, or ziprasidone. Elevated rates of metabolic effects (↑HbA1c, lipids) also observed.
Perez-Iglesias *et al.*, 2008	RCT, 12-month (first-episode)	164	Olanzapine vs. risperidone vs. haloperidol	**3 mo:** Olanzapine +8.4 kg; risperidone +5.9 kg; haloperidol +3.8 kg (olanzapine highest). **12 mo:** Olanzapine +10.9 kg; risperidone +8.9 kg; haloperidol +9.7 kg	$p = 0.002$ (3 mo); $p = 0.445$ (12 mo)	All treatments caused substantial weight gain (~9–11 kg in 1 yr) in drug-naïve patients. Olanzapine produced the most

Reference	Design	N	Exposure metric	Adiposity effect	p/CI	Notes
				(no significant difference at 1 yr).		rapid early weight gain (within 3 mo).

Animal Results

Table 2: Summary of animal studies on olanzapine exposure and adiposity outcomes.

Reference	Species	Design (duration)	Dose(s)	Adiposity effect	p/CI	Notes
Albaugh *et al.*, 2011	Rat (male)	Chronic oral gavage (5 weeks)	Escalating to 12 mg/kg/day	↑ Adiposity: body fat percentage increased significantly by ~2–3% (absolute) vs. controls, despite no total weight gain.	$P < 0.01$	Olanzapine increased fat mass (detected by week 1 and sustained) without increasing overall body weight. Mechanisms include reduced activity and altered energy partitioning.
Hou (Mao) *et al.*, 2018	Mouse (female)	Chronic oral gavage (7 weeks)	6 mg/kg/day	↑ Visceral adiposity: abdominal fat mass 0.20 g vs 0.13 g in controls (+54%); enlarged adipocytes in abdominal fat tissue.	$P < 0.05$	Olanzapine caused significant increases in intra-abdominal (visceral) fat and adipocyte size, along with mild weight gain (+0.94 g vs controls). Elevated leptin and triglycerides observed.
Yang *et al.*, 2019	Rat (female)	Subchronic (14 days)	2 mg/kg, twice daily (4 mg/kg/day)	↑ Body weight: significant weight gain evident by day 5–7 of olanzapine treatment (continuing through day 14). ↑ Adiposity: increased white adipose tissue mass (combined fat pads weight) in treated rats vs controls.	$P < 0.05$ (weight, fat)	Olanzapine-treated rats developed obesity-like metabolic syndrome: increased adiposity, hyperphagia, insulin resistance, hyperlipidemia, and inflammation. Dietary chromium supplementation partially mitigated these effects.

Conclusion

There is **strong evidence in humans** that olanzapine exposure increases adiposity. Multiple clinical trials and cohort studies consistently report significant weight gain in olanzapine-treated patients, with increases in BMI and fat mass (e.g. ~4–11 kg gain within weeks to a year). Notably, the weight gained is predominantly due to increased body fat rather than lean mass. In laboratory **animal models**, the evidence is also **strong** that olanzapine induces adiposity: numerous studies in rodents demonstrate increased body weight, elevated adipose tissue mass, and enlarged fat cells following olanzapine treatment. These effects have been observed across sexes and species (rats, mice), and even at doses or durations that do not always produce overall weight gain (as in male rats, where "hidden" fat accumulation still occurs). Overall, olanzapine consistently promotes obesity-related endpoints in both humans and animals, indicating a strong link between olanzapine exposure and increased adiposity.

References:

35. **Jain, S.**, Bhargava, M., & Gautam, S. (2006). *Weight gain with olanzapine: drug, gender or age?* Indian Journal of Psychiatry, 48(1), 39–42. PMID: 20703413 [1]

36. **Lieberman, J. A.**, Stroup, T. S., McEvoy, J. P., et al. (2005). *Effectiveness of antipsychotic drugs in patients with chronic schizophrenia* (CATIE trial). New England Journal of Medicine, 353(12), 1209–1223. DOI: 10.1056/NEJMoa051688 [2][3]

37. **Perez-Iglesias, R.**, Crespo-Facorro, B., Martinez-Garcia, O., et al. (2008). *Weight gain induced by haloperidol, risperidone and olanzapine after 1 year: findings of a randomized trial in a drug-naïve population.* Schizophrenia Research, 99(1–3), 13–22. DOI: 10.1016/j.schres.2007.10.022 [4]

38. **Eder, U.**, Mangweth, B., Ebenbichler, C., et al. (2001). *Association of olanzapine-induced weight gain with an increase in body fat.* American Journal of Psychiatry, 158(10), 1719–1722. DOI: 10.1176/appi.ajp.158.10.1719 [23]

39. **Albaugh, V. L.**, Judson, J. G., She, P., et al. (2011). *Olanzapine promotes fat accumulation in male rats by decreasing physical activity and increasing adipose tissue lipogenesis.* Molecular Psychiatry, 16(5), 569–581. DOI: 10.1038/mp.2010.33 [7][8]

40. **Hou, P.-H.**, Chang, G.-R., Chen, C.-P., et al. (2018). *Long-term administration of olanzapine induces adiposity and increases hepatic fatty acid desaturation protein in female C57BL/6J mice.* Iranian Journal of Basic Medical Sciences, 21(5), 495–501. DOI: 10.22038/IJBMS.2018.22759.5780 [11]

41. **Yang, C.-P.**, Wang, Y.-Y., Lin, S.-Y., et al. (2019). *Olanzapine-induced dysmetabolic changes involving tissue chromium mobilization in female rats.* International Journal of Molecular Sciences, 20(3), 640. DOI: 10.3390/ijms20030640 [15][16]

Pioglitazone

Human Results

Table 1: Human studies on pioglitazone exposure and adiposity

Reference	Design (Population)	N (subjects)	Exposure metric	Adiposity effect	p / 95% CI	Notes
**Smith et al., 2005**	RCT (T2DM patients, double-blind placebo-controlled)	48 (24 Pio, 24 placebo)	45 mg/day for 24 weeks	+3.9 kg body weight gain vs –0.8 kg in placebo; increased subcutaneous fat, no change in visceral fat	$p < 0.005$ (vs placebo)	Improved glycemic control (HbA1c –0.96% vs –0.11%) while inducing weight/fat gain.
**Dormandy et al., 2009** (PROactive)	RCT (T2DM with CVD, 3-year trial)	5,238 (pioglitazone vs placebo)	Up to 45 mg/day (median ~30 months)	+3.8 kg mean weight gain vs –0.6 kg in placebo group (≈4.4 kg net increase with pioglitazone)	$p < 0.001$ (vs placebo)	Notable dose-related weight gain; edema more frequent on pioglitazone (26% vs 15%).
**Kernan et al., 2016** (IRIS)	RCT (non-diabetic, insulin-resistant post-stroke/TIA patients)	3,876 (pioglitazone vs placebo)	Titrated to 45 mg/day, median 4.8 years	52.2% of pioglitazone group gained >4.5 kg vs 33.7% of placebo (significant increase in incidence of major weight gain)	$p < 0.001$	Also higher edema with pioglitazone (35.6% vs 24.9%); lowered progression to diabetes despite weight gain.
**Grossman et al., 2009**	Observational cohort (T2DM patients in 2-year surveillance)	1,527 (PIO) + 291 (non-TZD comparison)	Median 30 mg/day for 2 years	49.6% of pioglitazone users had weight gain vs 36.8% of comparators; mean weight change +2.19 kg (PIO) vs +0.34 kg (non-TZD)	OR 1.70, 95% CI 1.29–2.22	Pioglitazone associated with greater long-term weight gain and edema (adjusted OR for edema 1.92, 95% CI 1.32–2.79).

Animal Results

Table 2: Animal studies on pioglitazone exposure and adiposity

Reference	Species (Model)	Design	Dose(s)	Adiposity effect	p / 95% CI	Notes
**de Souza et al., 2001**	Rat (Zucker	28-day treatment	*Dose NR* (oral, daily)	Increased whole-body adiposity (↑ total fat mass by	$p < 0.05$ (reported)	Fat gain occurred in multiple depots

Reference	Species (Model)	Design	Dose(s)	Adiposity effect	p / 95% CI	Notes
	**fa/fa** obese)	vs controls		MRI; fat pad weights higher vs control); induced many new small adipocytes (adipocyte hyperplasia)		(especially visceral fat) despite improved insulin sensitivity.
**Kusunoki et al., 2011**	Rat (High-fat diet–induced IR)	7-week treatment (weeks 9–16 of HFD)	30 mg/kg/day (oral)	Pioglitazone alone caused large weight gain (Δ+127.8 g over 7 weeks, vs ~+76 g with co-treatment); significantly greater than in control or combination-treated rats	$p < 0.05$	High-fat diet controls developed obesity; co-administration of an LPL activator prevented ~40% of pioglitazone-induced weight gain.
**Matsuura et al., 2015**	Rat (Dahl **DS/obese** hybrid)	4-week treatment vs lean controls	2.5 mg/kg/day (oral)	Treated metabolic syndrome rats had increased body weight and fat mass (visceral and subcutaneous) vs baseline/controls, exacerbating obesity; adipocytes were smaller despite greater fat mass (indicative of adipose remodeling)	$p < 0.01$ (vs baseline)	Pioglitazone-treated obese rats showed "exacerbated obesity" but improved cardiac function and insulin resistance (PPARγ-mediated) in this model.
**Yu et al., 2023**	Mouse (Diet-induced obese)	16-week HFD + drug vs HFD control	*Approx.* 20 mg/kg/day (oral)	Greater body weight gain and increased total fat mass in pioglitazone-treated DIO mice vs untreated obese controls; white adipose depot weights unchanged, but brown adipose tissue weight	$p < 0.05$	Weight gain attributed partly to "brown fat whitening" (reduced thermogenesis) under pioglitazone, alongside improved glucose homeostasis in treated mice.

Reference	Species (Model)	Design	Dose(s)	Adiposity effect	p / 95% CI	Notes
				increased (browning to "whitening" with lipid)		

Conclusion

In **humans**, there is **strong evidence** that pioglitazone exposure increases adiposity: multiple clinical trials (in diabetics and non-diabetics) consistently report significant weight gain and increased fat mass in pioglitazone-treated groups compared to controls. In **animals**, the evidence is also **strong** – diverse rodent models (genetic obese rats, diet-induced obese rats and mice) demonstrate increased body weight and adipose tissue accumulation after pioglitazone treatment. The weight/fat gains are dose-dependent and reproducible, indicating a clear adipogenic effect of pioglitazone in both humans and animals.

References

- Smith, S. R., De Jonge, L., Volaufova, J., Li, Y., Xie, H., & Bray, G. A. (2005). *Effect of pioglitazone on body composition and energy expenditure: a randomized controlled trial*. Metabolism, 54(1), 24–32. DOI: 10.1016/j.metabol.2004.07.008.

- Dormandy, J. A., Bhattacharya, M., van Troostenburg de Bruyn, A. R., & PROactive Investigators. (2009). *Safety and tolerability of pioglitazone in high-risk patients with type 2 diabetes: an overview of data from PROactive*. Drug Safety, 32(3), 187–202. DOI: 10.2165/00002018-200932030-00002.

- Kernan, W. N., Viscoli, C. M., Furie, K. L., et al. (2016). *Pioglitazone after ischemic stroke or transient ischemic attack*. New England Journal of Medicine, 374(14), 1321–1331. DOI: 10.1056/NEJMoa1506930.

- Grossman, L. D., Parlin, G., Bailey, A. L., Yee, G., Yu, M., & Chan, J. Y. (2009). *Tolerability outcomes of a 2-year observational study of patients with type 2 diabetes treated with pioglitazone*. Clinical Therapeutics, 31(1), 74–88. DOI: 10.1016/j.clinthera.2009.01.004.

- de Souza, C. J., Eckhardt, M., Gagen, K., Dong, M., Chen, W., Laurent, D., & Burkey, B. F. (2001). *Effects of pioglitazone on adipose tissue remodeling in obesity and insulin resistance*. Diabetes, 50(8), 1863–1871. DOI: 10.2337/diabetes.50.8.1863.

- Kusunoki, M., Tsutsumi, K., Sato, D., Nakamura, A., Habu, S., Mori, Y., ... & Nakamura, T. (2011). *Pioglitazone-induced body weight gain is prevented by the lipoprotein lipase activator NO-1886 in high-fat fed rats*. European Journal of Pharmacology, 668(3), 486–491. DOI: 10.1016/j.ejphar.2011.07.030.

- Matsuura, N., Asano, C., Nagasawa, K., Ito, S., Sano, Y., Minagawa, Y., ... & Nagata, K. (2015). *Effects of pioglitazone on cardiac and adipose tissue pathology in rats with metabolic syndrome*. International Journal of Cardiology, 179, 360–369. DOI: 10.1016/j.ijcard.2014.11.099.

- Yu, P., Wang, W., Guo, W., Cheng, L., Wan, Z., Cheng, Y., Shen, Y., & Xu, F. (2023). *Pioglitazone-enhanced brown fat whitening contributes to weight gain in diet-induced obese mice*.

Experimental and Clinical Endocrinology & Diabetes, 131(11), 595–604. DOI: 10.1055/a-2178-9113.

Polychlorinated biphenyl 180 (PCB180)

HUMAN RESULTS

Table 1: Human Studies on PCB180 Exposure and Adiposity Outcomes

Reference (Year)	Design (Population)	N	Exposure Metric	Adiposity Effect	p / 95% CI	Notes
Dirinck et al. (2011)	Cross-sectional (obese vs. lean adults)	145	Serum PCB 180 (ng/mL)	**Inverse correlation:** higher BMI associated with *lower* PCB 180 levels in serum (obese had lower PCB 180)	$p < 0.05$ (correlation)	Persistent organic pollutants diluted in larger adipose mass; βHCH showed opposite (positive) correlation.
Lee et al. (2011)	Prospective cohort (young adults, 18-year follow-up)	~90	Serum PCB 180 (quartiles at baseline)	**Non-monotonic:** moderate PCB 180 levels predicted greater BMI increase over 18 years, but highest quartile had lesser BMI gain (inverted U-shaped association)	p (quadratic) < 0.05	PCB 180 (and other high-chlorinated PCBs) showed strongest BMI increase in mid exposure range, suggesting low-dose obesogenic effect.
Agay-Shay et al. (2015)	Birth cohort (Spain; prenatal exposure)	470	Maternal PCB 180 (serum in pregnancy)	**Positive association:** higher prenatal PCB 180 linked to higher BMI z-score at 7 years; increased overweight risk in children of highest-exposed mothers	RR = 2.59 (1.19–5.63)	Organochlorine factor (HCB, βHCH, PCB-138, PCB-180) in mixture associated with child overweight at 7, robust to adjustment for other EDCs.
Cohn et al. (2025)	Three-generation cohort (CHDS, USA)	258 families	Grandmaternal PCB "ratio" (sum of PCB 138 + 180 to PCB 153 in F0 serum)	**Positive association:** higher grandmaternal PCB 180/138 relative to 153 associated with higher odds of	OR = 1.73 (1.06–2.82) F1; OR = 1.96 (1.12–3.42) F2	F0 PCB mixture predicted obesity in two subsequent generations, even after accounting for F0 and F1 obesity status.

Reference (Year)	Design (Population)	N	Exposure Metric	Adiposity Effect	p / 95% CI	Notes
				obesity in daughters (F1) at 30 and granddaughters (F2) at 26		

ANIMAL RESULTS

Table 2: Animal Studies on PCB180 Exposure and Adiposity Outcomes

Reference (Year)	Species	Design (Exposure Duration)	Dose(s)	Adiposity Effect	p / Notes	Additional Notes
Viluksela et al. (2014)	Rat (adult)	28-day oral gavage (with loading + maintenance dosing)	0, 3, 10, 30, 100, 300, 1000, 1700 mg/kg total	**No obesity increase:** no significant body weight or fat gain; highest dose (1700 mg/kg) transiently **reduced** weight gain during dosing	(BMD modeling used; no p given)	Body weight catch-up occurred after initial suppression at the extreme dose; PCB180 showed toxicity (e.g. hepatic, endocrine effects) but lacked dioxin-like wasting or obesogenic effect.
Loiola et al. (2016)	Rat (adult)	15-day intranasal exposure (metabolic study)	0.1, 1, 10 μg/kg/day (PCB126 or PCB180)	**No obesity increase:** PCB 180 exposure did **not** induce body weight gain compared to controls (no difference in weight gain)	n.s. (no significant change)	Under the same protocol, dioxin-like PCB126 caused increased weight gain and metabolic effects, whereas non-dioxin PCB180 showed no such weight effect.

CONCLUSION

In humans, evidence linking PCB180 exposure to increased adiposity is **limited**. Some longitudinal studies (including prenatal and multigenerational cohorts) suggest that PCB180 exposure is associated with higher body mass index or obesity risk. However, cross-sectional data have shown inverse relationships between PCB180 levels and body size (attributed to toxicokinetic dilution in obese individuals). Overall, the human studies hint at obesogenic potential of PCB180, but findings are not uniformly consistent. In laboratory animals, there

is **no compelling evidence** of PCB180-induced obesity. Direct exposure experiments in adult rats have not shown increased body weight or adipose tissue gain from PCB180; if anything, very high doses suppressed weight gain rather than promoting it. Thus, the current strength of evidence is **limited in humans** and **none in animals** for PCB180 as an obesogen.

References:

- Dirinck, E., Jorens, P.G., Covaci, A., Geens, T., Roosens, L., Neels, H., Mertens, I., & Van Gaal, L. (2011). *Obesity and persistent organic pollutants: possible obesogenic effect of organochlorine pesticides and polychlorinated biphenyls*. **Obesity (Silver Spring)**, 19(4), 709–714[1][2].

- Lee, D.H., Steffes, M.W., Sjödin, A., Jones, R.S., Needham, L.L., & Jacobs, D.R. Jr. (2011). *Low dose organochlorine pesticides and polychlorinated biphenyls predict obesity, dyslipidemia, and insulin resistance among people free of diabetes*. **PLoS ONE**, 6(1), e15977[3].

- Agay-Shay, K., Martínez, D., Valvi, D., Garcia-Esteban, R., Basagaña, X., Robinson, O., Casas, M., Sunyer, J., & Vrijheid, M. (2015). *Exposure to endocrine-disrupting chemicals during pregnancy and weight at 7 years of age: A multi-pollutant approach*. **Environ. Health Perspect.**, 123(10), 1030–1037[4].

- Cohn, B.A., Cirillo, P.M., La Merrill, M.A., Murphy, C.C., Hu, X., & Krigbaum, N.Y. (2025). *Grandmaternal perinatal serum PCBs and prevalence of obesity in adult daughters and granddaughters*. **Obesity**, 33(??), ePub ahead of print[5][6].

- Viluksela, M., Heikkinen, P., van der Ven, L.T.M., Rendel, F., Roos, R., Esteban, J., Korkalainen, M., Lensu, S., Miettinen, H.M., Savolainen, K., & Håkansson, H. (2014). *Toxicological profile of ultrapure 2,2′,3,4,4′,5,5′-heptachlorobiphenyl (PCB 180) in adult rats*. **PLoS ONE**, 9(8), e104639[7].

- Loiola, R.A., Dos Anjos, F.M., Shimada, A.L., Cruz, W.S., Drewes, C.C., Rodrigues, S.F., Cardozo, K.H.M., Carvalho, V.M., Pinto, E., & Farsky, S.H. (2016). *Long-term in vivo polychlorinated biphenyl 126 exposure induces oxidative stress and alters the proteomic profile of islets of Langerhans* (includes PCB180 comparison). **Scientific Reports**, 6, 27882[9].

Rosiglitazone Exposure and Adiposity Outcomes

HUMAN RESULTS

Multiple human studies indicate that rosiglitazone treatment is associated with increased adiposity (weight gain and fat mass). Key findings are summarized in Table 1.

Table 1:

Table 1: Reference	Design	N	Exposure metric	Adiposity effect	p/CI	Notes
**Kahn et al., 2006** (ADOPT trial)	RCT (double-blind, 4-year) vs. metformin	4,360	Rosiglitazone 4–8 mg/day	+5.6 kg body weight (mean at 4 years)	$p < 0.0001$ (vs comparators)	Significantly greater weight gain than metformin or glyburide.

Table 1: Reference	Design	N	Exposure metric	Adiposity effect	p/CI	Notes
	& glyburide in T2DM					
**Punthakee et al., 2014** (DREAM sub-study)	RCT (3.5-year) in pre-diabetes (CT & DEXA scans)	190	Rosiglitazone 8 mg/day	+4.1 kg total body fat vs placebo	$p < 0.0001$	Increased subcutaneous fat (+31 cm² abdominal SAT), with a relative decrease in visceral fat after adjustment.
**Ratziu et al., 2008** (FLIRT trial)	RCT (placebo-controlled, 1-year) in NASH patients	63	Rosiglitazone 4→8 mg/day	+1.5 kg body weight (vs −1.0 kg placebo)	$p < 0.01$	Significant weight gain compared to placebo.

In all above human trials, rosiglitazone-treated groups showed greater gains in body weight or fat mass than controls.

ANIMAL RESULTS

In laboratory animal models, direct exposure to rosiglitazone consistently leads to increased adiposity. Table 2 highlights results from animal studies.

Table 2:

Table 2: Reference	Species	Design	Dose(s)	Adiposity effect	p/CI	Notes
**Pickavance et al., 1999**	Rat (diet-induced obese)	21-day feeding experiment (obese vs lean rats)	0.3–30 mg/kg/day	↑ Body weight (+50 g at highest dose) and +20–24% fat pad mass	$p < 0.0001$ (weight); $p < 0.05$ (fat)	Rosiglitazone caused hyperphagia and increased adiposity in obese rats.
**Pini et al., 2012**	Mouse (C57BL/6)	Dietary exposure (rosiglitazone 0.01% in diet) vs control diet (8 weeks)	~0.01% in chow (~high dose)	↑ Body weight (+9–11%) and fat mass (+23–73%) vs control diet	$p < 0.05$ (for both)	Significant gains in fat mass in both lean and high-fat-diet–obese mice.

CONCLUSION

Strength of evidence: There is **strong evidence** in humans (multiple RCTs and clinical studies) that rosiglitazone exposure increases adiposity (body weight and fat mass). Similarly, in animal models, **strong evidence** from direct exposure experiments shows rosiglitazone causes significant increases in body weight and adipose tissue mass.

References:

42. Kahn, S. E., Haffner, S. M., Heise, M. A., et al. (2006). Glycemic durability of rosiglitazone, metformin, or glyburide monotherapy. *New England Journal of Medicine, 355*(23), 2427–2443.
43. Pickavance, L. C., Tadayyon, M., Widdowson, P. S., Buckingham, R. E., & Wilding, J. P. H. (1999). Therapeutic index for rosiglitazone in dietary obese rats: separation of efficacy and haemodilution. *British Journal of Pharmacology, 128*(7), 1570–1576.
44. Pini, M., Rhodes, D. H., Castellanos, K. J., Cabay, R. J., Grady, E. F., & Fantuzzi, G. (2012). Rosiglitazone improves survival and hastens recovery from pancreatic inflammation in obese mice. *PLoS ONE, 7*(7), e40944.
45. Punthakee, Z., Alméras, N., Després, J.-P., et al. (2014). Impact of rosiglitazone on body composition, hepatic fat, fatty acids, adipokines and glucose in people with impaired glucose tolerance: a sub-study of the DREAM trial. *Diabetic Medicine, 31*(9), 1086–1092.
46. Ratziu, V., Giral, P., Jacqueminet, S., et al. (2008). Rosiglitazone for nonalcoholic steatohepatitis: one-year results of the randomized placebo-controlled FLIRT trial. *Gastroenterology, 135*(1), 100–110.

Sertraline

Human Results

Table 1: Effects of sertraline on adiposity outcomes in human studies.

Reference	Design	N	Exposure metric	Adiposity effect	p/CI	Notes
Gafoor et al. (2018)	Population cohort (UK)	294,719	Antidepressant (AD) use vs none	Higher incidence of ≥5% weight gain episodes vs non-use (11.2 vs 8.1 per 100 person-yrs)	RR = 1.21 (1.19–1.22), $p < 0.001$	10-year follow-up; risk persisted ≥6 years. AD use increased odds of transitioning from normal weight to overweight/obesity (RR ≈ 1.29).
Mwinyi et al. (2024)	Prospective cohort (CH)	2,479	SSRI (sertraline) use during 5.5-year follow-up	Steeper BMI increase in sertraline users vs non-users (no significant waist gain)	β≈+0.3 (adjusted); $p < 0.05$	SSRI users had higher odds of ≥5% BMI gain (43% vs 29%; OR ~1.63). Sertraline use linked to BMI rise but not waist circumference change.
Petimar et al. (2024)	EHR cohort (USA)	183,118	Initiation of antidepressant (comparative)	Modest weight gain on sertraline (~0.2–0.3 kg	–	24-month follow-up. Weight gain with sertraline was intermediate: less than with escitalopram or

Reference	Design	N	Exposure metric	Adiposity effect	p/CI	Notes
				at 6 months, est.)		paroxetine, but more than with bupropion. E.g., escitalopram users gained +0.41 kg and bupropion users −0.22 kg relative to sertraline at 6 months.

Animal Results

Table 2: Effects of sertraline on adiposity outcomes in animal studies.

Reference	Species	Design	Dose(s)	Adiposity effect	p/CI	Notes
Silverstein-Metzler et al. (2016)	Cynomolgus monkey (♀)	18-month RCT (placebo vs sertraline)	20 mg/kg/day (oral)	Prevented diet-induced increases in body weight and fat mass (no weight gain in treated vs ↑ weight in controls)	$p < 0.05$	Western diet feeding; sertraline group had no significant weight/fat gain, plus lower insulin and HOMA-IR vs placebo. Slight reduction in adiponectin observed in depressed subgroup.
Nielsen et al. (1992)	Rat (♂), ob/ob mouse (♂)	Short-term dosing experiment	10–32 mg/kg (rat), 44 mg/kg (mouse), i.p. BID	Inhibited weight gain; dose-dependent weight loss at higher doses	$p < 0.05$	5-day treatment in rats: high-dose sertraline caused weight *loss* (−34 g vs +37 g in controls). 12-day treatment in obese mice: sertraline lowered body weight vs controls with no tolerance to effect.

Conclusion

In humans, **moderate evidence** indicates that sertraline (an SSRI antidepressant) is associated with increased adiposity (weight gain and higher BMI) during long-term treatment. In contrast, in animals, there is **no evidence** of sertraline exposure increasing adiposity; available studies actually show *decreased* weight/fat gain with sertraline treatment. Thus, sertraline demonstrates a moderate weight-gain effect in humans, whereas in animal models evidence for obesity endpoints is **absent or inconsistent**.

References:

47. Mwinyi, J., Strippoli, M.-P. F., Kanders, S. H., Schiöth, H. B., Eap, C. B., Lasserre, A. M., Marques-Vidal, P., Vandeleur, C. L., & Preisig, M. (2024). *Long-term changes in*

adiposity markers during and after antidepressant therapy in a community cohort. Translational Psychiatry, 14(1), 330. DOI: 10.1038/s41398-024-03032-5.
48. Gafoor, R., Booth, H. P., & Gulliford, M. C. (2018). *Antidepressant utilisation and incidence of weight gain during 10 years' follow-up: population based cohort study*. BMJ, 361, k1951. DOI: 10.1136/bmj.k1951.
49. Petimar, J., Young, J. G., Yu, H., Rifas-Shiman, S. L., Daley, M. F., Heerman, W. J., Janicke, D. M., Jones, W. S., Lewis, K. H., Lin, P. I. D., Prentice, C., Merriman, J. W., Toh, S., & Block, J. P. (2024). *Medication-induced weight change across common antidepressant treatments: a target trial emulation study*. Annals of Internal Medicine, 177(8), 993–1003. DOI: 10.7326/M23-2742.
50. Silverstein-Metzler, M. G., Shively, C. A., Clarkson, T. B., Appt, S. E., & Kaplan, J. R. (2016). *Sertraline inhibits increases in body fat and carbohydrate dysregulation in adult female cynomolgus monkeys*. Psychoneuroendocrinology, 68, 29–38. DOI: 10.1016/j.psyneuen.2016.02.021.
51. Nielsen, J. A., Chapin, D. S., Johnson, J. L., Jr., & Torgersen, L. K. (1992). *Sertraline, a serotonin-uptake inhibitor, reduces food intake and body weight in lean rats and genetically obese mice*. American Journal of Clinical Nutrition, 55(1 Suppl), 185S–189S. DOI: 10.1093/ajcn/55.1.185s.

Tetrabromobisphenol A (TBBPA)

Human Results

Table 1. Summary of human observational studies on TBBPA exposure and adiposity outcomes.

Reference	Design	N	Exposure metric	Adiposity effect	p/CI	Notes
Liang et al., 2020	Prospective birth cohort (China)	2023 mother–infant pairs	Maternal serum TBBPA (pregnancy)	↓ Birth weight in male infants (inverse association)	$P<0.01$ (males)(trend p=0.007)	Higher prenatal TBBPA associated with lower birth weight (and length) in **male** newborns; no significant association in females. No human studies reported positive (obesogenic) associations.

Animal Results

Table 2. Summary of animal studies of TBBPA exposure and adiposity outcomes.

Reference	Species	Design (exposure duration)	Dose(s)	Adiposity effect	p/CI	Notes
**Ding et al., 2025**	Mouse (C57BL/6J, male)	Diet-induced obesity model (120 d)	20 & 50 nmol/kg/day in high-fat diet	↑ Body weight (+12%) and ↑ fat mass at 50	$p<0.05$ (50 nmol)	120-day chronic low-dose TBBPA exposure

Reference	Species	Design (exposure duration)	Dose(s)	Adiposity effect	p/CI	Notes
				nmol/kg; no lean mass change		(environmentally relevant doses) in HFD-fed mice significantly increased adiposity and weight gain at the high dose. Also induced glucose intolerance and insulin resistance. Effects were observed under excess calorie (HFD) conditions.
**Tian et al., 2021**	Zebrafish (adult male)	Adult exposure experiment (8 weeks)	20, 100, 500 µg/L in water	↑ Food intake (hyperphagia) and ↑ adiposity (obesity with hepatic fat accumulation)	p<0.05	Environmentally relevant concentrations led to overeating and obesity in male zebrafish. Mechanistic analysis showed activation of cannabinoid receptor 1 (endocannabinoid pathway) driving increased appetite and fat deposition.
**Cope et al., 2015**	Rat (Sprague–Dawley)	90-day oral gavage (plus multigeneration study)	0, 100, 300, 1000 mg/kg/day	No increase in body or fat weight (no obesogenic effect at any dose)	n.s.	High-dose TBBPA showed no clear adverse effects on growth or adiposity in standard diet conditions (NOAEL = 1000 mg/kg·day). Consistent with other subchronic rat studies reporting low toxicity and no weight gain at high doses.

Conclusion

In humans, **no evidence** currently links TBBPA exposure to increased obesity or adiposity (in fact, the only human cohort found an inverse association with neonatal birth size). Therefore, the human evidence for TBBPA as an obesogen is **none to very limited**. In animals, there is **limited evidence** that TBBPA can act as an obesogen under certain conditions. Adult male zebrafish and high-fat-fed mice showed significant increases in adiposity and weight gain with TBBPA exposure. However, traditional rodent toxicology studies (e.g. 90-day rat studies) observed no weight or fat increase at even very high doses, indicating inconsistent results. Overall, the animal data suggest **limited/inconsistent evidence** of TBBPA's obesogenic effects (manifesting in specific models such as diet-induced obesity or zebrafish, but not universally across all studies), and **no compelling epidemiological evidence in humans** to date.

References:

- Ding, Y., Zhang, T., Ma, H.-B., Han, J., Zhu, W., Zhao, X., Lu, X., Zhou, B., & Shi, X.-J. (2025). *Chronic Exposure to Environmental Concentrations of Tetrabromobisphenol A Disrupts Insulin and Lipid Homeostasis in Diet-Induced Obese Mice*. Environmental Science & Technology, 59(9), 4330–4343[5][6].

- Liang, J., Liu, S., Liu, T., Yang, C., Wu, Y., Tan, H. J. J., Wei, B., Ma, X., Feng, B., Jiang, Q., Huang, D., & Qiu, X. (2020). *Association of prenatal exposure to bisphenols and birth size in Zhuang ethnic newborns*. Chemosphere, 252, 126422[2].

- Tian, S., Yan, S., Meng, Z., Huang, S., Sun, W., Jia, M., Teng, M., Zhou, Z., & Zhu, W. (2021). *New insights into bisphenols-induced obesity in zebrafish (Danio rerio): Activation of cannabinoid receptor CB1*. Journal of Hazardous Materials, 418, 126100[7][8].

- Cope, R. B., Kacew, S., & Dourson, M. (2015). *A reproductive, developmental and neurobehavioral study following oral exposure of tetrabromobisphenol A in Sprague–Dawley rats*. Toxicology, 329, 49–59[9].

Thiacloprid

HUMAN RESULTS:

Epidemiological studies report mixed findings on thiacloprid exposure and adiposity. Some cross-sectional studies in children note positive associations with adiposity measures, while others find inverse or null associations. Table 1 summarizes human evidence.

Table 1: Human Studies on Thiacloprid Exposure and Adiposity

Reference	Design	N	Exposure Metric	Adiposity Effect	p/CI	Notes
Yang et al. (2024)	Cross-sectional (children, China)	442	Urinary NEOs (8 insecticides incl. thiacloprid,	*Mixed:* Detection of certain NEOs (e.g. clothianidin) linked to slightly higher BMI z-score; high	OR=2.16 (1.28–3.63) for central obesity at medium	Thiacloprid measured but not individually associated with BMI; combined pesticide burden showed an

Reference	Design	N	Exposure Metric	Adiposity Effect	p/CI	Notes
			+4 metabolites)	acetamiprid levels linked to lower BMI z-score. Medium-level combined NEO exposure (incl. thiacloprid) associated with increased central obesity (WC≥90th percentile).	exposure vs. none.	effect on waist circumference.
Lu et al. (2023)	Cross-sectional (7-year-olds, China)	380	Urinary NEOs (7 insecticides + 2 metabolites)	*Positive:* Thiacloprid exposure (77.6% detected) associated with higher waist-to-height ratio (WHtR, indicator of abdominal obesity). Association largely mediated by oxidative stress (8-iso-$PGF_{2}\alpha$ biomarker).	Mediation by 8-iso-$PGF_{2}\alpha$ explained ~70% of THD–WHtR link (p for mediation <0.05).	Higher imidacloprid levels also correlated with increased odds of overweight/obesity. Thiacloprid's effect on WHtR suggests potential obesogenic influence via oxidative stress.
Wu et al. (2024)	Cross-sectional (adolescents, China)	524	Urinary NEOs (11 insecticides + metabolites)	*Inverse:* Thiacloprid concentration associated with **lower** prevalence of general obesity. Higher urinary thiacloprid was linked to reduced odds of being obese.	OR=0.28 (95% CI: 0.08–0.99) for general obesity (high vs. low THD).	Other NEOs (e.g. nitenpyram, clothianidin) showed positive associations with BMI or abdominal obesity. Indicates possible confounding or age-specific effects for thiacloprid.

ANIMAL RESULTS

Experimental animal studies have not shown thiacloprid to increase adiposity; if anything, high-dose exposures led to weight loss or no change in fat endpoints. Table 2 summarizes key animal findings.

Table 2: Animal Studies on Thiacloprid and Adiposity

Reference	Species	Design	Dose(s)	Adiposity Effect	p/CI	Notes
Smith et al. (2020)	Mouse (C57BL/6J)	Perinatal exposure to pesticide *cocktail* (boscalid, captan, chlorpyrifos, thiacloprid, ziram) during gestation; offspring challenged with high-fat diet in adulthood (metabolic outcomes measured at 6 and 14 weeks)	0.25 mg/kg/day each (gestational days 0–18)	*No increase:* Perinatal thiacloprid (within mixture) did **not** affect offspring body weight, fat mass or energy homeostasis in adulthood. No heightened susceptibility to high-fat diet-induced obesity observed.	n.s. (no significant differences vs. controls)	Mixture contained thiacloprid; even combined low-dose exposures showed **no obesogenic effect** in male or female offspring. Metabolic profiles (glucose, lipids) were unaltered by the pesticide mixture.
Mahmoud et al. (2024)	Rat (Wistar, male)	8-week oral exposure (subchronic reproductive toxicity study)	22.5 and 62.1 mg/kg/day (gavage, 56 days)	*Weight loss:* Thiacloprid caused significant **decrease** in body weight (dose-dependent). Treated rats had lower final body weights and reduced adipose (testicular fat) weights vs. controls.	$p < 0.05$ (body weight reduction)	High-dose thiacloprid induced systemic toxicity (e.g. reduced food intake, oxidative stress). No indications of increased fat accumulation; instead, overall growth was suppressed. Weight loss accompanied reproductive impairment in males.

CONCLUSION: In **humans**, the evidence linking thiacloprid to obesity is **inconsistent**. Some studies in children report that higher thiacloprid exposure correlates with greater adiposity (especially abdominal adiposity), but other data show null or even inverse associations. These mixed findings (and potential confounding factors) yield only limited and inconclusive support for thiacloprid as an obesogen in human populations. In **animals**, there is **no evidence** that thiacloprid increases adiposity. To the contrary, available rodent studies show either **no effect** on weight/fat gain or **decreased** body weights at high doses due to toxicity. Overall, the current evidence does **not** strongly implicate thiacloprid in promoting obesity-

related endpoints in either humans or experimental animals (strength of evidence: **inconsistent in humans; none in animals**).

References:

52. **Yang Z. et al. (2024).** Urinary neonicotinoids and metabolites are associated with obesity risk in Chinese school children. *Environment International*, 183:108366[3][4].

53. **Lu Z. et al. (2023).** Urinary neonicotinoid insecticides and adiposity measures among 7-year-old children in northern China: A cross-sectional study. *International Journal of Hygiene and Environmental Health*, 251:114188[5][6].

54. **Wu X. et al. (2024).** Urinary neonicotinoid concentrations and obesity: A cross-sectional study among Chinese adolescents. *Environmental Pollution*, 345:123516[7][8].

55. **Smith L. et al. (2020).** Perinatal exposure to a dietary pesticide cocktail does not increase susceptibility to high-fat diet–induced metabolic perturbations at adulthood but modifies urinary and fecal metabolic fingerprints in C57Bl/6J mice. *Environment International*, 144:106010[10].

56. **Mahmoud A.A.N. et al. (2024).** Thiacloprid impairs reproductive functions of male Wistar rats. *Naunyn Schmiedeberg's Archives of Pharmacology*, 397(8):6197–6211[11].

Tributyltin (TBT) Exposure and Adiposity Outcomes

Human Results

Table 1: Epidemiological Evidence in Humans (TBT Exposure and Adiposity)

Reference (year)	Study Design	N	Exposure Metric	Adiposity Outcome	p/CI	Notes
Rantakokko *et al.* (2014)	Prospective cohort (Finland)	110	Placental TBT (ng/g)	↑ Infant weight gain 0–3 months; no effect on length or later growth	p = 0.024 (0–3 mos)	TBT detected in 99% of placentas; association with early postnatal weight gain only (no sustained effect beyond 3 months). No other human studies to date have linked TBT with obesity.

Animal Results

Table 2: Experimental Animal Studies (TBT Exposure and Adiposity Outcomes)

Reference (year)	Species (Sex)	Design (Exposure Period)	Dose(s)	Adiposity Effect	p/CI	Notes
Grün *et al.* (2006)	Mouse (M/F)	Prenatal (gestational	0.1 mg/kg (gavage to	↑ Neonatal adipose lipid accumulation;	$p < 0.05$ (significant)	First evidence of "obesogen" effect

Reference (year)	Species (Sex)	Design (Exposure Period)	Dose(s)	Adiposity Effect	p/CI	Notes
		day 16, single dose)	pregnant dam)	↑ adult epididymal fat mass in offspring vs. controls		of TBT in vivo; in utero TBT exposure led to excess fat at birth and increased fat depot weight in young adult offspring. No increase in overall body weight was observed in adulthood despite higher fat mass.
Penza *et al.* (2011)	Mouse (F)	Post-weaning to adult (peripubertal exposure, 60 days)	0.5 µg/kg/day (oral)	↑ Body weight; ↑ white fat mass (vs. vehicle)	$p < 0.05$	Low-dose TBT (comparable to human intake) caused significant weight and fat gain in female mice. Adipogenic effects observed in both sexes at doses as low as 0.5 µg/kg.
Zuo *et al.* (2011)	Mouse (M)	Young adult exposure (45 days)	0.5, 5, 50 µg/kg/day (gavage)	↑ Body weight gain (dose-dependent); ↑ gonadal (testicular) fat pad weight; hepatic steatosis; hyperinsulinemia	$p < 0.05$	Significant increases in adiposity at ≥5 µg/kg; highest dose induced fatty liver and elevated insulin/leptin levels, indicative of obesity and metabolic dysfunction.
He *et al.* (2014)	Rat (M & F)	Adult exposure (54 days)	0.5 µg/kg/day (oral)	Males: ↑ final body weight and ↑ fat mass; Females: no fat gain but ↑ food intake	$p < 0.01$ (M vs F)	Both sexes had ↑ hepatic triglycerides & cholesterol. TBT caused weight/fat increase only in males, while females showed hyperphagia without increased adiposity, suggesting sex-

Reference (year)	Species (Sex)	Design (Exposure Period)	Dose(s)	Adiposity Effect	p/CI	Notes
						specific effects on energy balance.
Li *et al.* (2017)	Mouse (M)	Adult exposure (chronic + recovery)	50 µg/kg q3d, 45 days (i.p.), then withdrawal	↑ Body weight, ↑ serum insulin, glucose, adiponectin; ↓ glucagon during exposure	$p < 0.05$	Metabolic syndrome-like changes induced by TBT were largely reversible after exposure cessation (body weight and glucose normalized), but some endocrine effects (low adiponectin, low glucagon) persisted post-exposure.
Ceotto Freitas-Lima *et al.* (2018)	Rat (F)	Adult exposure (subacute)	100–500 ng/kg/day (oral, 2 weeks)	↑ Body weight; insulin resistance; adipose tissue inflammation (vs. control)	$p < 0.05$	Low-dose TBT in adult female rats led to abnormal white adipose tissue expansion with inflammatory changes and impaired glucose homeostasis. Consistent with other studies linking TBT to metabolic syndrome features in females.

Conclusion

In **human studies**, evidence that tributyltin increases adiposity is **limited to none**. Only a small cohort study found a transient increase in early infant weight gain associated with prenatal TBT exposure, and no long-term or adult obesity associations have been demonstrated in humans to date. In contrast, **animal evidence** is **strong**, with numerous rodent experiments consistently showing that TBT exposure (at environmentally relevant doses) causes increased adipose tissue mass and weight gain, alongside metabolic disturbances. These findings – observed across multiple studies in mice and rats of both sexes – provide robust support that TBT acts as an obesogenic chemical in animals. The concordance of outcomes (greater fat deposition, altered metabolic hormones, etc.) across independent studies strengthens the conclusion that TBT exposure can induce adiposity in

animal models. However, the translation of these effects to humans remains uncertain given the paucity of epidemiological data.

References:

57. Rantakokko, P., Main, K. M., Wohlfart-Veje, C., *et al.* (2014). *Association of placenta organotin concentrations with growth and ponderal index in 110 newborn boys from Finland during the first 18 months of life: a cohort study*. **Environmental Health, 13**(1), 45. DOI: 10.1186/1476-069X-13-45.

58. Grün, F., Watanabe, H., Zamanian, Z., *et al.* (2006). *Endocrine-disrupting organotin compounds are potent inducers of adipogenesis in vertebrates*. **Molecular Endocrinology, 20**(9), 2141–2155. DOI: 10.1210/me.2005-0367.

59. Penza, M., Jeremic, M., Marrazzo, E., *et al.* (2011). *The environmental chemical tributyltin chloride (TBT) shows both estrogenic and adipogenic activities in mice which might depend on the exposure dose*. **Toxicology and Applied Pharmacology, 255**(1), 65–75. DOI: 10.1016/j.taap.2011.05.017.

60. Zuo, Z., Chen, S., Wu, T., *et al.* (2011). *Tributyltin causes obesity and hepatic steatosis in male mice*. **Environmental Toxicology, 26**(1), 79–85. DOI: 10.1002/tox.20531.

61. He, K., Zhang, J., & Chen, Z. (2014). *Effect of tributyltin on the food intake and brain neuropeptide expression in rats*. **Endokrynologia Polska, 65**(6), 485–490. DOI: 10.5603/EP.2014.0068.

62. Li, B., Guo, J., Xi, Z., Xu, J., Zuo, Z., & Wang, C. (2017). *Tributyltin in male mice disrupts glucose homeostasis as well as recovery after exposure*. **Archives of Toxicology, 91**(10), 3261–3269. DOI: 10.1007/s00204-017-1961-6.

63. Freitas-Lima, L. C., Merlo, E., Zicker, M. C., *et al.* (2018). *Tributyltin impacts metabolic syndrome development through disruption of angiotensin II receptor signaling pathways in white adipose tissue of adult female rats*. **Toxicology Letters, 299**, 21–31. DOI: 10.1016/j.toxlet.2018.08.018.

Triphenyl phosphate

Human Results

Table 1: Epidemiological studies on TPP exposure and adiposity outcomes in humans

Reference (APA)	Design	N	Exposure Metric	Adiposity Effect	p / 95% CI	Notes
Boyle *et al.*, 2019	Cross-sectional (NHANES 2013–14)	784 children; 1672 adults	Urinary DPHP (TPhP metabolite)	**No significant association** with BMI or obesity in either children or adults (null findings).	– ($p > 0.05$)	Other OPE metabolites showed some associations, but **DPHP was not consistently associated** with

Reference (APA)	Design	N	Exposure Metric	Adiposity Effect	p / 95% CI	Notes
						any adiposity measure.
Li *et al.*, 2024	Cross-sectional (NHANES 2017–18)	1,334 adults	Urinary DPHP (spot, log_2-transformed)	**Minimal association:** Each doubling of DPHP was associated with 9% higher odds of obesity in minimally adjusted model, but **no significant association after full adjustment** (effect attenuated).	OR 1.09 (1.01–1.17), p = 0.02 (minimally adjusted); n.s. after full adjustment.	Average age ~45; controlled for demographics and lifestyle. Only other metabolites (BCEP, BDCPP) retained significance in fully adjusted models.
Chen *et al.*, 2023	Prospective birth cohort (Shanghai)	733 mother–child pairs	Maternal urinary DPHP (12–16 wk gestation)	**No clear association for DPHP**; higher maternal levels of other OPE metabolites (BCIPP, BCEP, BDCPP, DCP) were linked to **greater child adiposity** (weight/BMI z-scores) up to 6 years, but only among children with <4 months breastfeeding. DPHP was not significantly associated with child adiposity.	– (effect estimates for DPHP not significant; p > 0.05)	Gestational exposure measured in early pregnancy. **Breastfeeding >4 months mitigated effects** of OPE exposures on child adiposity. No significant impact observed for TPhP's metabolite (DPHP) in this cohort.
Peterson *et al.*, 2024	Prospective multi-cohort (ECHO study)	5,087 mother–child pairs	Maternal urinary OPEs during pregnancy (including DPHP)	**No association for TPhP**: Higher prenatal DPHP exposure was *not* significantly associated with obesity risk in children (age 5–10) – i.e., children of high TPhP-exposed mothers had similar obesity	OR ~1 (n.s., exact CI not reported)	Large US consortium of 14 cohorts (2006–2020 births). Assessed child BMI status through age 10. **Mixed results across OPEs:** TPhP showed *no effect* on childhood obesity

Reference (APA)	Design	N	Exposure Metric	Adiposity Effect	p / 95% CI	Notes
				odds as low-exposed. (By contrast, DBP metabolites were linked to higher obesity risk, and BDCPP to lower risk.)		risk, while other OPE biomarkers had divergent associations.

Animal Results

Table 2: In vivo studies on TPP exposure and adiposity outcomes in animals

Reference (APA)	Species	Design	Dose(s)	Adiposity Effect	p / 95% CI	Notes
Green *et al.*, 2017	Rat (UCD-T2DM model)	**Perinatal exposure** (maternal feed from GD 8.5 to weaning; offspring evaluated at 3.5 months)	170 µg TPhP per dam per day (≈0.4–0.6 mg/kg/day)	**Increased body weight and fat mass** in adult offspring of both sexes. Developmental TPhP exposure led to heavier body weight and larger fat depots at 3.5 months. Also elevated leptin (males) and higher energy intake (females) were observed.	$p < 0.05$ (for increased fat/weight)	UCD-T2DM rats are predisposed to metabolic disease. **Perinatal TPhP exacerbated obesity development** in male *and* female offspring, and accelerated onset of diabetes in males.
Wang *et al.*, 2019	Mouse (C57BL/6)	**Developmental exposure** (in utero + lactational via dam from gestation through nursing); offspring fed low-fat or high-fat diet until adulthood	10, 100, 1000 µg/kg BW per day (gestational gavage)	**Increased adiposity and weight** in adult male offspring. Prenatal+postnatal TPhP exposure led to **higher body weight, increased fat mass, enlarged liver, and hepatic steatosis** in offspring, along with impaired	$p < 0.05$ (significant increases in weight, fat mass, etc.)	Effects observed by adulthood (with or without high-fat diet). **Fetal TPhP exposure promoted obesity** and metabolic dysfunction in offspring mice. Altered lipid metabolism gene expression and gut microbiome

Reference (APA)	Species	Design	Dose(s)	Adiposity Effect	p / 95% CI	Notes
				glucose tolerance and insulin resistance.		changes were also noted.
Tachachartvanich *et al.*, 2024	Mouse (C57BL/6)	**Perinatal exposure** (gestational through lactational exposure; offspring examined in adulthood; mechanistic intervention with inhibitor)	*Not reported* (low-dose TPhP via maternal route; plus subset given EGFR inhibitor)	**Increased adiposity in male offspring:** Male mice perinatally exposed to TPhP showed a **significant increase in adiposity** (greater fat accumulation), elevated hepatic triglycerides, and insulin resistance in adulthood. (Female outcomes not highlighted in abstract.) Co-treatment with an EGFR blocker reversed the increased adiposity and metabolic disturbances.	$p < 0.05$ (for adiposity and metabolic effects in males)	Reinforces that **developmental TPhP exposure induces obesity and metabolic dysfunction** in male mice. Identified an EGFR/ERK/AKT signaling mechanism for TPhP's obesogenic effect, as pharmacologically blocking EGFR normalized the offspring's metabolic profile.
Li *et al.*, 2025	Mouse (BALB/c)	**Adult exposure** (direct dosing of adult mice for 60 days; plus parallel in vitro adipocyte tests)	0, 1, 10, 150 mg/kg/day (oral, 60 days)	**Dose-dependent adiposity gain in males:** In adult male mice, TPhP caused **dose-related increases in fat depots (inguinal fat weight)** and adipocyte hypertrophy. Adipogenesis-related genes were	$p < 0.05$ (significant trend in males); n.s. in females	Demonstrates sex-specific effects of TPhP in adults: **males were susceptible to TPhP-induced adiposity**, whereas females were resistant. Multi-omics indicated TPhP activates PPARγ and PI3K/AKT pathways in adipose tissue,

Reference (APA)	Species	Design	Dose(s)	Adiposity Effect	p / 95% CI	Notes
				upregulated in male adipose tissue. **Female mice did not show significant fat/mass changes** under the same treatment.		consistent with an "environmental obesogen" mode of action.

Conclusion

In **humans**, the evidence linking Triphenyl phosphate exposure to increased adiposity is **inconsistent/limited**. Epidemiological studies show mostly null or weak associations between TPP (or its metabolite DPHP) and obesity-related outcomes. A few reports suggest slight increases in BMI or child weight with higher exposure, but these findings are not robust and often attenuate after adjusting for confounders or differ by subgroup. Overall, current human data do not consistently support a causal relationship between TPP and obesity endpoints.

In **animals**, there is **strong evidence** that TPP exposure can induce adiposity and weight gain. Multiple controlled studies in rodents (mice and rats) have demonstrated that developmental or adult TPP exposures lead to increased body weight, fat mass, and related metabolic disorders. The obesogenic effects are reproducible across different laboratories and exposure scenarios – notably observed in perinatal exposure models (sometimes with male-specific sensitivity) as well as in adult males. Taken together, the animal studies provide a coherent indication that TPP acts as a metabolism-disrupting chemical promoting obesity, whereas human evidence is presently limited and not conclusive.

References:

- Boyle, M., Buckley, J. P., & Quirós-Alcalá, L. (2019). *Associations between urinary organophosphate ester metabolites and measures of adiposity among U.S. children and adults: NHANES 2013–2014*. **Environment International, 127**, 754–763.[1]

- Li, H., Li, F., Zhou, C., Bu, J., Yang, H., Zhong, L., *et al.* (2024). *Exposure to organophosphate flame retardants is associated with obesity and dysregulated serum lipid profiles: Data from 2017–2018 NHANES*. **Metabolites, 14**(2), 124.[2]

- Chen, Y., Zhang, X., Wang, Z., Yuan, Z., Luan, M., Yuan, W., *et al.* (2023). *Gestational exposure to organophosphate esters and adiposity measures of children up to 6 years: Effect modification by breastfeeding*. **International Journal of Hygiene and Environmental Health, 248**, 114089.[3]

- Peterson, A. K., Alexeeff, S. E., Ames, J. L., et al. (2024). *Gestational exposure to organophosphate ester flame retardants and risk of childhood obesity in the ECHO cohort*. **Environment International, 193**, 109071.[4]

- Wang, D., Yan, S., Yan, J., Teng, M., Meng, Z., Li, R., *et al.* (2019). *Effects of triphenyl phosphate exposure during fetal development on obesity and metabolic dysfunctions in adult mice: Impaired lipid metabolism and intestinal dysbiosis*. **Environmental Pollution, 246**, 630–638.[8]
- Li, T., Liu, Y., Cao, J., Lu, X., Lu, Y., Wang, Y., *et al.* (2025). *Triphenyl phosphate induces lipid metabolism disorder and promotes obesity through PI3K/AKT signaling pathway*. **Environment International, 198**, 109428.[10]
- Green, A. J., Graham, J. L., Gonzalez, E. A., La Frano, M. R., Petropoulou, S. S. E., Park, J. S., *et al.* (2017). *Perinatal triphenyl phosphate exposure accelerates type 2 diabetes onset and increases adipose accumulation in UCD-Type 2 Diabetes Mellitus rats*. **Reproductive Toxicology, 68**, 119–129.[6]
- Tachachartvanich, P., Rusit, X., Tong, J., Mann, C., & La Merrill, M. A. (2024). *Perinatal triphenyl phosphate exposure induces metabolic dysfunctions through the EGFR/ERK/AKT signaling pathway: Mechanistic in vitro and in vivo studies*. **Ecotoxicology and Environmental Safety, 269**, 115756.[9]

Triphenyltin (639-58-7) and Adiposity Outcomes

Human Results

No epidemiological or clinical study has directly evaluated the effects of triphenyltin (TPT) exposure on adiposity in humans. One small birth cohort (Finland, n=110 boys) measured organotin levels in placentas and infant growth. In that study, higher placental tributyltin (TBT) concentrations were significantly associated with greater weight gain from birth to 3 months of age (p=0.024), but no associations were observed beyond 3 months. Triphenyltin was detected in only ~43% of placentas (levels an order of magnitude lower than TBT) and showed no significant relationship with infant weight or length gain. Thus, human evidence that TPT increases adiposity or obesity endpoints is absent.

Table 1: Human studies on TPT exposure and adiposity

Reference	Design	N	Exposure Metric	Adiposity Effect	p-value / CI	Notes
Rantakokko et al., 2014	Nested case–control analysis within prospective birth cohort (Finland)	110 infants (55 cryptorchid cases, 55 controls)	Organotin levels in placenta (TBT, TPhT, etc.)	Higher TBT in placenta associated with greater infant weight gain 0–3 months; no effect on weight 3–18 months; TPhT showed no association	TBT: p=0.024 (3rd vs 1st tertile weight gain); TPhT: n.s.	TPhT was <LOQ in 57% of samples and ~10× lower concentration than TBT; study limited to early-life weight gain (no long-term obesity assessed)

Animal Results

In laboratory animal studies, there is no evidence that TPT exposure increases adiposity; in contrast, high-dose TPT often led to reduced weight gain or developmental delays. **Hamsters:** In a 180-day feeding study, hamsters given triphenyltin chloride in diet (up to ~55 ppm) showed significantly **less** body-weight gain at mid and high doses compared to controls, despite no other overt toxicity. **Rats:** Developmental exposure to TPT caused growth suppression rather than obesity. For example, maternal TPT (triphenyltin chloride) at 2 mg/kg/day (gestation through lactation) resulted in male offspring with significantly decreased postnatal weight gain and delayed sexual maturation, while female offspring's body weights were unaffected. A higher dose (6 mg/kg) was perinatally lethal, precluding evaluation. **Mice:** In a perinatal study, pregnant/lactating mice received TPT (fentin hydroxide) up to 7.5 mg/kg/day. Offspring were born at significantly lower weight across all TPT doses, but they exhibited normal weight gain by postnatal day 5 onward (no lasting differences in body weight or adiposity). No increases in fat mass or obesity-related endpoints were observed in these offspring into adulthood. Overall, across animal models (rodents), TPT has not been shown to promote adipose tissue gain or obesity; if anything, higher exposures impaired growth rather than inducing excess adiposity.

Table 2: Animal studies on TPT exposure and adiposity

Reference	Species	Design	Dose(s)	Adiposity Effect	p-value / CI	Notes
Ohhira et al., 1996	Golden hamster	180-day subchronic feeding study (dietary TPT chloride)	1.28, 28.82, 54.77 ppm in feed	**Reduced** weight gain at mid and high doses vs control (no adiposity increase)	p<0.05 for weight gain suppression at ≥28.8 ppm (significant vs control)	No clinical toxicity signs aside from growth attenuation; TPT rapidly metabolized in vivo
Grote et al., 2009	Rat (offspring)	Prenatal + lactational exposure (gavage to dams GD6–PND21; offspring evaluated at ~PND60)	0, 2, 6 mg/kg/day TPTCl	**Decreased** postnatal body weight gain in male pups at 2 mg/kg; female pup weights unchanged (no adiposity increase)	p<0.05 for lower male weight vs control; 6 mg/kg group not fully evaluated due to high neonatal mortality	Male offspring also had reduced reproductive organ weights and delayed puberty; females showed only minor effects (earlier vaginal opening)
Mello et al., 2010	Mouse (offspring)	Maternal exposure during gestation and lactation (gavage) with offspring	0, 1.875, 3.75, 7.5 mg/kg/day TPT (fentin) hydroxide	**Initial growth retardation**: offspring birth weight significantly lower at all doses, but no differences in weight or fat by	p<0.05 for reduced birth weight at all doses; postnatal weight trajectories not significantly different from	Maternal toxicity minimal (↑liver weight at 7.5 mg/kg; some litters lost at high dose). Offspring had normal development

Reference	Species	Design	Dose(s)	Adiposity Effect	p-value / CI	Notes
		followed to adulthood		weaning and adulthood (no obesity observed)	controls after neonatal period	and fertility in adulthood

Conclusion

Human evidence: *None.* There is no human study demonstrating that triphenyltin exposure increases adiposity or obesity; the only available human data (infant growth in a small cohort) did not find any association for TPT specifically. **Animal evidence:** *None.* The animal studies to date provide no evidence that TPT induces adiposity; in fact, at the doses tested, TPT exposure tended to impede weight gain or had no long-term effect on body fat. Thus, the overall evidence does not support an obesogenic effect of triphenyltin in either humans or animals.

References:

- Rantakokko, P., Main, K. M., Wohlfart-Veje, C., Kiviranta, H., Airaksinen, R., Vartiainen, T., … & Virtanen, H. E. (2014). *Association of placenta organotin concentrations with growth and ponderal index in 110 newborn boys from Finland during the first 18 months of life: a cohort study*. Environmental Health, 13:45. DOI: 10.1186/1476-069X-13-45. PMID: 25141990.
- Ohhira, S., Matsui, H., & Nitta, K. (1996). *Subchronic study of the metabolism of triphenyltin in hamsters*. Veterinary and Human Toxicology, 38(3), 206–209. PMID: 8727222.
- Grote, K., Hobler, C., Andrade, A. J. M., Grande, S. W., Gericke, C., Talsness, C. E., … & Chahoud, I. (2009). *Sex differences in effects on sexual development in rat offspring after pre- and postnatal exposure to triphenyltin chloride*. Toxicology, 260(1–3), 53–59. DOI: 10.1016/j.tox.2009.03.006. PMID: 19464569.
- Mello, M. S. de C., Lopes, C. M. T., Delgado, I. F., & Paumgartten, F. J. R. (2010). *Postnatal development and fertility of offspring from mice exposed to triphenyltin (fentin) hydroxide during pregnancy and lactation*. Journal of Toxicology and Environmental Health, Part A, 73(13–14), 965–971. DOI: 10.1080/15287391003751752. PMID: 20574909.
